# A trial of intra-pleural bacterial immunotherapy in malignant pleural mesothelioma (TILT) — a randomised feasibility study using the trial within a cohort (TwiC) methodology

**DOI:** 10.1186/s40814-022-01156-3

**Published:** 2022-09-03

**Authors:** Anna C. Bibby, Natalie Zahan-Evans, Emma Keenan, Charles Comins, John E. Harvey, Helen Day, Najib M. Rahman, Janet E. Fallon, Rachael Gooberman-Hill, Nick A. Maskell

**Affiliations:** 1 Academic Respiratory Unit, University of Bristol Medical School, Bristol, UK; 2 North Bristol Lung Centre, North Bristol NHS Trust, Bristol, UK; 3 Bristol Haematology & Oncology Centre, University Hospitals Bristol NHS Foundation Trust, Bristol, UK; 4 Oxford Respiratory Trials Unit, Nuffield Department of Experimental Medicine, University of Oxford, Oxford, UK; 5 Respiratory Department, Musgrove Park Hospital, Somerset NHS Foundation Trust, Taunton, UK; 6 NIHR Biomedical Research Centre at University Hospitals Bristol and Weston NHS Foundation Trust and the University of Bristol, Bristol, UK

**Keywords:** Mesothelioma, Immunotherapy, Intra-pleural therapy, Feasibility, Trials within cohorts

## Abstract

**Background:**

Malignant pleural mesothelioma (MPM) is an aggressive thoracic malignancy with a poor prognosis. Systemic immunotherapy is an effective frontline treatment for MPM, and there is a scientific rationale supporting the possible efficacy of local, i.e. intra-pleural immune modulators. Trial of intra-pleural bacterial immunotherapy (TILT) investigated the feasibility of performing a randomised trial of intra-pleural bacterial immunotherapy in people with MPM, using the trials within cohorts (TwiC) methodology.

**Methods:**

TILT was a multicentre, three-armed, randomised, feasibility TwiC of intra-pleural OK432, BCG, or usual care in people with MPM. Eligible participants were identified from within the ASSESS-meso study, a prospective, longitudinal, observational cohort study, and were randomly selected to be offered a single dose of OK432 or BCG, via an indwelling pleural catheter. The primary outcome was feasibility, evaluated against prespecified recruitment, attrition and data completeness targets. The acceptability of trial processes and interventions was assessed during qualitative interviews with participants and family members at the end of the trial. TILT was registered prospectively on the European Clinical Trials Registry (EudraCT number 2016–004,727-23) and the ISRCTN Register on 04 December 2017.

**Results:**

Seven participants were randomised from a planned sample size of 12; thus, the 66% recruitment rate target was not met. Two participants withdrew after randomisation, breaching the pre-stated attrition threshold of 10%. It was not possible to maintain blinding of control participants, which negated a fundamental tenet of the TwiC design. The trial processes and methodology were generally acceptable to participants and relatives, despite several recipients of intra-pleural bacterial agents experiencing significant local and systemic inflammatory responses.

**Conclusion:**

It was possible to design a clinical trial of an investigational medicinal product based on the TwiC design and to obtain the necessary regulatory approvals. However, whilst acceptable to participants and relatives, the TwiC design was not a feasible method of investigating intra-pleural bacterial immunotherapy in people with MPM. Future trials investigating this topic should consider the eligibility constraints and recruitment difficulties encountered.

**Trial registration:**

TILT was registered prospectively on the European Clinical Trials Registry (EudraCT number 2016-004727-23) and the ISRCTN Register (10432197) on 04 December 2017.

**Supplementary Information:**

The online version contains supplementary material available at 10.1186/s40814-022-01156-3.

## Strengths and limitations of the study

- This is the first reported Clinical Trial of an Investigational Medicinal Product (CTIMP) that uses the “trials within cohorts” design.
- As a feasibility study, TILT highlighted potential challenges and limitations to conducting a full-scale trial on intra-pleural bacterial immunotherapy in mesothelioma, with significantly less resource expenditure than a full-scale trial.
- Using a mixed-methods design, TILT combined quantitative and qualitative data to evaluate the feasibility of the trial and its acceptability to participants and relatives.
- TILT was at risk of selection and survivorship bias, two factors that have affected previous mesothelioma clinical trials.

## Introduction

Malignant pleural mesothelioma (MPM) is an aggressive thoracic malignancy caused by prior asbestos exposure. It carries a poor prognosis, with median survival less than 1 year from diagnosis [1–5]. Treatment options are limited, although there have been several important breakthroughs in the therapeutic landscape in recent years [6, 7].

One such breakthrough is related to immune checkpoint inhibitors (ICI), agents which block activation of inhibitory receptors on effector T cells [8, 9]. Combination ICI regimens extended survival in a recent randomised trial in treatment-naïve MPM patients and have demonstrated early promise in non-randomised post-frontline trials [7, 10, 11]. However, the toxicity profiles of combination ICI are significant, and this may reduce the appeal of these agents to patients, many of whom are reluctant to receive systemic anticancer treatment due to concern about side effects [12–14].

Topical administration of therapeutic agents into the pleural space could limit systemic absorption, leading to fewer side effects, whilst maximising drug concentration in the immediate tumour environment [15, 16]. Bacterial products have been administered into the pleural space for decades with the aim of stimulating local immune responses to generate pleurodesis [17–19]. However, our recent systematic review demonstrated a lack of high-quality evidence regarding the relationship between intra-pleural bacterial products and survival among people with pleural malignancy [20]. This area of research was highlighted as important in the James Lind Alliance priority setting partnership in 2015 [21].

The trial of intra-pleural bacterial immunotherapy (TILT) was designed to address this question, focussing on two bacterial products: OK432 and BCG. OK432 consists of heat-treated, penicillin-killed, freeze-dried *Streptococcus pyogenes* group A2 (Picibanil, Chugai Pharmaceutical Ltd., Japan), whilst BCG is a live-attenuated, low-virulence strain of *Mycobacterium bovis* prepared from a culture of Bacillus Calmette-Guérin (OncoTice, Merck Sharp & Dohme Ltd., the Netherlands). These bacterial products were chosen based on in vitro and in vivo evidence of pro-inflammatory activity and cytotoxic effects [22–25].

The trial was based on the pragmatic trials within cohorts (TwiC) methodology (also known as the cohort multiple randomised controlled trials, cmRCT) [26]. Patients are screened for trial eligibility from within a longitudinal, observational cohort study, with eligible participants selected at random to be offered the trial intervention. Nonselected eligible participants act as controls from the cohort. A key tenet of the TwiC design is that participants are only informed about the trial intervention once they have been selected to receive it, whilst controls are blinded to the existence of the trial.

The TwiC design has several potential benefits, including efficient recruitment, reduced cross over between arms and reduced attrition. TwiCs can reduce disappointment if patients enrol in a trial in the hope of receiving a treatment that is not otherwise available but are allocated to the control arm [26–28]. As a pragmatic design, TwiCs replicate real-life clinical care more faithfully than standard RCTs and thus provide useful information on the effectiveness of an intervention [26, 27, 29]. Before TILT, the TwiC design had not been applied to trials in MPM, nor had it been used for a drug trial (known in the UK as a clinical trial of an investigational medicinal product or CTIMP). Consequently, a feasibility trial was planned. Specific areas of uncertainty related to whether a CTIMP TwiC could secure the necessary approvals from Research Ethics Committees (REC), the NHS Health Research Authority (HRA) and the UK Medicines and Healthcare Products Regulatory Authority (MHRA), whether participants would consent to join the trial and receive the investigational medicinal product (IMP) after randomisation and whether the design would be acceptable to participants in both the active treatment and the control arms.

The aim of TILT was to determine whether it was feasible to perform a TwiC of intra-pleural OK432 or BCG compared with standard care in people with MPM and whether it was acceptable to participants and their partners, relatives and carers. During setup, it became clear that certain considerations were necessary to maintain the core concept of TwiCs whilst ensuring compliance with Good Clinical Practice and international clinical trials regulations. These considerations have been published elsewhere [30].

## Methods

### Cohort overview

TILT was embedded within a prospective, multicentre cohort study of people with mesothelioma, called ASSESS-meso (ISRCTN 61861764). Patients were eligible to participate in ASSESS-meso if they had a multidisciplinary team-confirmed diagnosis of mesothelioma and were willing and able to comply with study follow up assessments.

On enrolment to ASSESS-meso, patients were asked for their consent to be screened for future trials, to be randomly selected to join those trials and to provide comparative data for trials even if not selected to join them. Participants who did not wish to be considered for future trials were welcome to enrol in ASSESS-meso but were not eligible for TILT.

### Trial overview

TILT was a multicentre, three-arm (1:1:1), randomised feasibility trial of intra-pleural OK432 vs intra-pleural BCG vs usual care in people with MPM, using the TwiC methodology. TILT was registered on the European Clinical Trials Registry (EudraCT number 2016–004,727-23) and the ISRCTN Register (10432197) and approved by the Research Ethic Committee (ref 17/SW/0080), MHRA (18524/0228/001–0002) and NHS HRA (IRAS ID 215394).

### Participants and setting

Patients undergoing follow-up in ASSESS-meso at one of three hospitals in England were screened, between 27/01/2018 and 31/11/2019. To be suitable for TILT, patients were required to have a pathologically confirmed diagnosis of MPM, have a functioning indwelling pleural catheter (IPC) in situ (or be suitable for IPC insertion), not be receiving chemotherapy and have a performance status of ≤ 3 with a predicted life expectancy of at least 12 weeks (see Appendix A for trial protocol, including full eligibility criteria). Exclusion criteria included non-expandable lung, moderate or heavily loculated effusion, active infection (pleural or elsewhere), recent (< 2 weeks) thoracic surgery, known immunosuppression or immune modulating medication, brain metastases or allergy to either IMP or penicillin.

### Randomisation and enrolment

Eligibility was screened at every cohort study visit, including enrolment. Randomisation took place at the first time point that the eligibility criteria were met. Participants were not informed when randomisation occurred.

Randomisation was undertaken by a member of the trial team, using a centralised, concealed randomisation module within the online study database (REDCap, Vanderbilt, USA). Randomisation occurred on a 1:1:1 basis, using a block randomisation sequence, with blocks of varying and random sizes, stratified by performance status (assessed on the day of randomisation, after drainage of fluid, and graded as 0 or ≥ 1) and tumour subtype (classified as epithelioid versus non-epithelioid). The randomisation sequence was generated by an independent database administrator using STATA (StataCorp LP) version 15 and was concealed from the trial team.

Participants were allocated to receive intra-pleural OK432, intra-pleural BCG, or usual care, where usual care consisted of active surveillance and supportive management (other treatments, including thoracic surgery, current chemotherapy, or immunotherapy, were contraindications to trial enrolment for safety reasons). The clinical trial team was unblinded to the outcome of randomisation, as were participants allocated to receive an IMP. On allocation to one of the intervention arms, participants were informed about the trial and invited to give written informed consent to participate. Participants allocated to the control arm remained blinded to both the fact of randomisation and its outcome and continued follow up in ASSESS-meso on an altered visit schedule that matched TILT follow-up. Participants who declined to receive an IMP after being randomised to receive it also returned to follow-up in ASSESS-meso.

### IMP administration

The IMP was delivered as a single dose, via an indwelling pleural catheter, within 14 days of randomisation, in accordance with the Trial Specific Procedure (Appendix B). The original dose of OK432 was 10 KE and of BCG 0.4–1.6 × 10^7^ CFU, based on previous dose finding and clinical efficacy trials [31–33]. However, after the first three participants had been enrolled, an urgent safety measure (USM) was passed by the data monitoring committee (DMC) in response to early adverse reactions, advising half dose (i.e. 5KE of OK432, 0.2–0.8 × 10^7^ CFU of BCG) be used in participants considered high risk of adverse events, specifically elderly patients, patients with performance status of 2–3 and patients with multiple cardiac or renal co-morbidities.

### Trial outcomes and data collection

The primary outcome was feasibility. The Trial Steering Committee determined that the trail would be considered feasible if the following criteria were met:

- Recruitment rate of ≥ 66% to time and target
- Attrition rate of < 10% after randomisation, where attrition was defined as participants who declined to receive an IMP following randomisation or who declined or failed to complete follow up in the cohort if allocated to control
- Data completeness rates > 90%

Additionally, specific features of the TwiC design were evaluated, including the number of control participants who were unblinded, the number and characteristics of cohort participants who declined to be considered for trials and the acceptability of trial processes to participants and family members, evaluated during qualitative interviews.

Secondary outcomes included adverse events (AE), exploratory efficacy data and PROMs. AEs were assessed for severity (based on the Common Terminology Criteria for Adverse Events v5.0), expectedness and relatedness to IMP. AE data was reviewed by the Data Monitoring Committee (DMC) who had the capacity to close the trial early or suggest modifications to the protocol if significant safety concerns arose.

Exploratory efficacy measurements included survival, radiological tumour response rates, pleural fluid drainage volumes and pleurodesis rates. Survival was calculated as date of diagnosis with MPM to date of death, as recorded on the death certificate. Surviving participants were censored on 02 June 2020. Radiological response rates were assessed on computed tomography (CT) scans at baseline and week 12 by an independent thoracic radiologist, blinded to trial allocation, using mRECIST criteria [34]. Pleural fluid drainage volumes were recorded by community nursing staff who was not involved in the trial and were unaware of patients’ participation status. Pleurodesis was defined as pleural fluid drainage of less than 50 ml on 3 consecutive occasions, with < 25% opacification on CXR or < 250 ml pleural fluid on thoracic ultrasound scanning (TUS) and was evaluated by blinded clinicians, independent of the trial.

Symptom scores for breathlessness, chest pain and sweats were completed by the patients at each visit using a 10 cm VAS, where 0 represented no symptom at all and 100 represented the worst severity of that symptom. Quality of life (QoL) was evaluated using the EQ-5D-5L questionnaire, completed by participants at every study visit.

**Fig. 1:**
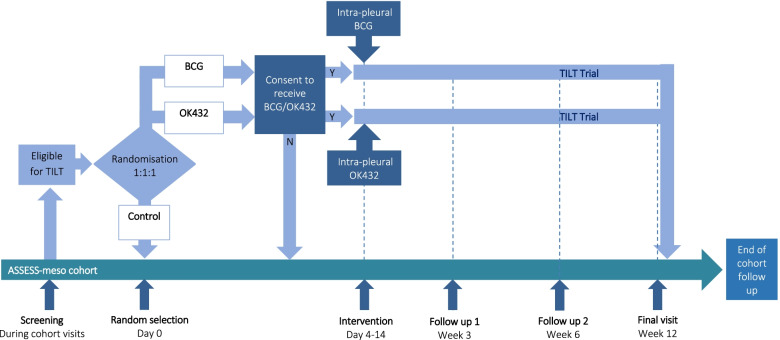
TILT trial schema

The trial schema is shown in Fig. 1. Data were collected at baseline and weeks 3, 6 and 12. On completion of the trial, participants returned to follow up within ASSESS-meso. Data collection for ASSESS-meso continued until death, loss to follow-up or study withdrawal.

### Sample size

As a feasibility trial, the sample size needed to be “adequate to estimate the critical [feasibility] parameters to the necessary degree of precision” [35]. The “critical feasibility parameter” for TILT was determined to be post-randomisation attrition, as this TwiC-specific element was untested in mesothelioma patients. An attrition threshold of ≥ 20% was deemed to be unfeasible; as for a full-scale trial requiring an estimated 300 participants (100 per arm), this would result in loss of approximately 60 people. Based on this, an attrition rate of 10% with 95% CI of ± 10% was used in the sample size calculation. Initial sample size was 45 participants, which was sufficient to detect a 10% attrition rate with 95% CI of ± 9%. The target sample size was reduced after 18 months in response to slower than anticipated recruitment but (at that point) zero attrition. The new estimated attrition rate was 5%, which could be detected with a sample size of 12 people with 95% CI of ± 12%.

### Statistical analysis

Descriptive statistics were used to summarise recruitment, attrition and data completeness rates, which were compared with the prespecified feasibility criteria.

Participant characteristics were tabulated according to allocation at randomisation, i.e. intention to treat (ITT). Secondary outcomes were summarised for each arm, based on allocation at randomisation. Because of the small number of participants, people randomised to receive OK432 or BCG were combined, post hoc, to form one IMP group.

Survival data were analysed using unadjusted and adjusted Cox proportional hazards modelling. Pleurodesis rates and radiological response rates were compared between groups using Fisher’s exact test. Outcomes with repeat measurements were analysed at each trial visit using two-way analysis of variance (ANOVA) with multiple regression, based on ITT. Statistical analysis was undertaken using Stata (StataCorp LP) version 15.

### Qualitative interviews

To assess acceptability, face-to-face interviews were carried out with participants and relatives after completion of the 12-week trial period. Interviews were conducted by the first author (ACB) who used a topic guide to focus interviews on the experience of participating in TILT and the acceptability of the trial processes and TwiC design. Interviews were audio recorded, transcribed verbatim and analysed thematically [36]. The full qualitative methodology and results have been published elsewhere [37].

## Results

### Participant characteristics

Between the recruitment dates of 27 January 2018 and 31 November 2019, seven participants were randomised. The trial was stopped on a prespecified date, based on IMP expiry dates. Three were allocated to receive BCG, one to receive OK432 and three were designated as controls. Six participants were male; all seven participants had epithelioid MPM. One participant had received four cycles of palliative cisplatin and pemetrexed chemotherapy; the remaining patients were all treatment naive (Table 1).

**Table 1:** Baseline characteristics of TILT participants. All values given are *n* (%) unless otherwise stated

	**All participants**	**OK432**	**BCG**	**Control**
**Total**	7	1	3	3
**Male**	6 (85.7)	-	3 (100)	3 (100)
**Age**, median (range)	73 (60–83)	64	71 (60–73)	80 (73–83)
**Performance status**				
0	3 (42.9)	-	2 (66.7)	1 (33.3)
1	2 (28.5)	1 (100)	-	1 (33.3)
2	1 (14.3)	-	-	1 (33.3)
3	1 (14.3)	-	1 (33.3)	-
**Asbestos exposure**				
None recalled	1 (14.3)	1 (100)	-	-
Transient	1 (14.3)	-	-	1 (33.3)
Light/passive	1 (14.3)	-	1 (33.3)	-
Heavy/active	4 (57.1)	-	2 (66.7)	2 (66.7)
**Presenting symptoms**				
Breathlessness	5 (71.4)	1 (100)	2 (66.7)	2 (66.7)
Chest pain	1 (14.3)	-	1 (33.3)	-
Cough	3 (42.9)	-	2 (66.7)	1 (33.3)
Sweats	-	-	-	-
Lethargy	1 (14.3)	-	1 (33.3)	-
Anorexia	1 (14.3)	-	-	1 (33.3)
Weight loss	1 (14.3)	-	-	1 (33.3)
Asymptomatic	1 (14.3)	-	-	1 (33.3)
**Duration of symptoms**				
< 1 month	3 (42.9)	1 (100)	1 (33.3)	1 (33.3)
1–3 months	1 (14.3)	-	-	1 (33.3)
> 3 months	2 (28.6)	-	2 (66.7)	-
Not recorded	1 (14.3)	-	-	1 (33.3)
**Method of diagnosis**				
CT-guided biopsy	1 (14.3)	1 (100)	-	-
Medical thoracoscopy	5 (71.4)	-	3 (100)	2 (66.7)
VATS	1 (4.3)	-	-	1 (33.3)
**Laterality**				
Left	2 (28.6)	-	1 (33.3)	1 (33.3)
Right	5 (71.4)	1 (100)	2 (66.7)	2 (66.7)
**Tumour histology**				
Epithelioid	7 (100)	1 (100)	3 (100)	3 (100)
**Previous treatment**				
Chemotherapy	1 (100)	1 (100)	-	-
Radiotherapy	-	-	-	-
Surgery	-	-	-	-
Bevacizumab	-	-	-	-
Immunotherapy	-	-	-	-
**Brims prognostic score**				
1 (best prognosis)	1 (14.3)	-	-	1 (33.3)
2	5 (71.4)	1 (100)	3 (100)	1 (33.3)
3	-	-	-	-
4 (worst prognosis)	1 (14.3)	-	-	1 (33.3)

### Primary outcome — feasibility

The pre-stated feasibility goal of recruiting > 66% of the target population of 12 was not met. During the 22-month recruitment period, seven participants were randomised, yielding an overall recruitment rate of 58.3% of target (7/12). Furthermore, of those seven participants, two withdrew after randomisation: one from the BCG arm and one from the control arm. This created an attrition rate of 28.6%, breaching the prespecified criteria of < 10%. Data completeness was high, with only 60 missing values over 8750 data points (data completeness rate 99.3%), exceeding that of > 90% data completeness feasibility criteria. Most of the missing data is related to control participants at visits 2 and 3.

**Fig. 2:**
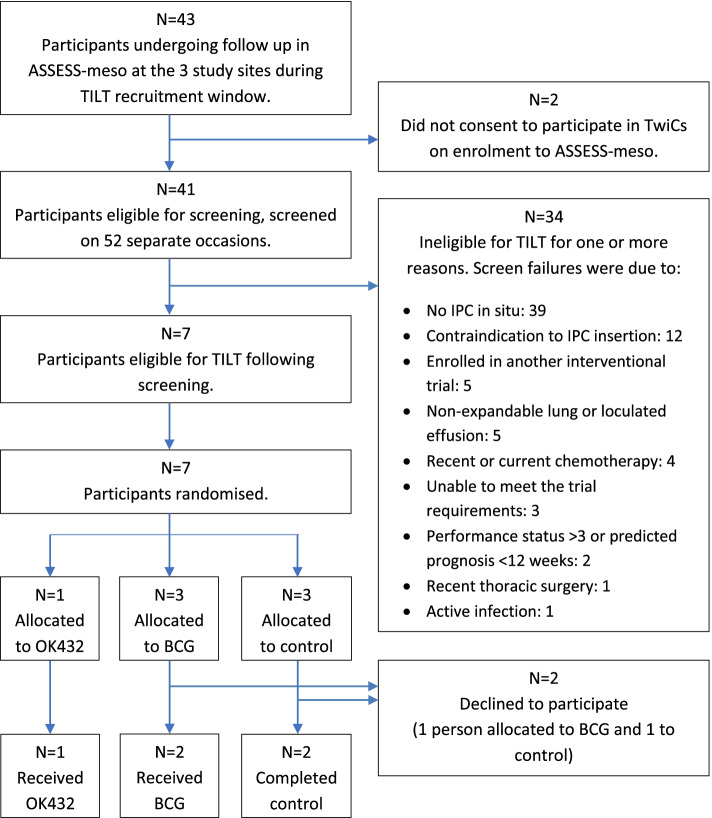
Flow chart demonstrating participant screening, eligibility and enrolment in TILT

Review of screening logs yielded further information around recruitment. Forty-three participants were undergoing follow-up in ASSESS-meso at the recruiting centres during the enrolment period. Of these, two people had chosen not to participate in TwiCs. The remaining 41 patients were screened on 52 occasions. The seven participants randomised for TILT were the only people to meet the eligibility criteria at any point (Fig. 2).

The most frequent reason that patients were ineligible was absence of a functioning IPC (39 screen failures), which often co-existed with a contra-indication to IPC insertion (12 screen failures). The presence of non-expandable lung and/or a loculated effusion was the cause of five screen failures. Five participants were enrolled in an alternative interventional trial, whilst four participants were ineligible due to recent or concurrent chemotherapy.

Qualitative interviews with the two participants who withdrew from the trial provided insight into their decision-making. Both patients wished to prioritise quality of life and were concerned that participating in the trial may compromise this. One patient, allocated to BCG, was reluctant to receive a trial medication that carried a risk of side effects, whilst the other patient, allocated to control, did not wish to return to hospital as frequently as the trial schedule required.

### TwiC-specific features — blinding of controls

Qualitative interviews revealed that all seven TILT participants had been unblinded to the existence of the trial prior to undergoing randomisation. Three participants had been involved in patient and public involvement groups at which TILT was discussed, months before the trial design was chosen. Two participants became aware of TILT after hearing other patients discussing it at local mesothelioma support groups. Two participants had been told about TILT by clinicians at non-trial centres who knew about the trial but were unaware of the specific requirement for blinding.

### TwiC-specific features — consent to be randomised for TwiCs

Only four out of 91 (4.4%) ASSESS-meso participants did not wish to be screened or randomised for future trials. Patient numbers were too small to perform statistical comparisons; however, patients who chose not to be considered for future TwiCs appeared similar to the wider ASSESS-meso study population (Table 2).

**Table 2:** Characteristics of ASSESS-meso participants who chose not to be considered for future TwiCs. All values given are *n* (%) unless otherwise stated

	**Participants who did not wish to be considered for TwiCs**	**All ASSESS-meso participants**
**Total**	4	91
**Male**	3 (75)	77 (84.6)
**Age**, median (range)	79 (64–93)	74 (33–93)
**Performance status**		
0	2 (50)	30 (33.0)
1	2 (50)	42 (46.2)
2	-	17 (18.7)
3	-	2 (2.2)
**Asbestos exposure**		
None recalled	-	14 (15.4)
Transient	-	11 (12.1)
Light/passive	3 (75)	20 (22.0)
Heavy/active	1 (25)	46 (50.5)
**Presenting symptoms**		
Breathlessness	2 (50)	72 (79.1)
Chest pain	1 (25)	32 (35.1)
Cough	3 (75)	38 (41.8)
Sweats	1 (25)	12 (13.2)
Lethargy	-	20 (22.0)
Anorexia	-	11 (12.1)
Weight loss	1 (25)	25 (27.5)
Asymptomatic	1 (25)	3 (3.3)
**Duration of symptoms**		
< 1 month	2 (50)	21 (23.1)
1–3 months	1 (25)	39 (42.9)
> 3 months	1 (25)	28 (30.8)
Asymptomatic	-	3 (3.3)
**Method of diagnosis**		
US-guided biopsy	1 (25)	10 (11.0)
CT-guided biopsy	-	8 (8.8)
Medical thoracoscopy	2 (50)	46 (50.6)
VATS	-	16 (17.6)
Other biopsy (e.g. laparoscopic)	-	5 (5.5)
Cytological	1 (25)	4 (4.4)
Clinico-radiological	-	2 (2.2)
**Disease site**		
Pleural	4 (100)	88 (96.7)
Peritoneal	-	3 (3.3)
**Laterality**		
Left	2 (50)	38 (41.8)
Right	2 (50)	50 (55.0
Peritoneal	-	3 (3.3)
**Tumour histology**		
Epithelioid	3 (75)	72 (79.1)
Sarcomatoid	-	10 (11.0)
Biphasic	-	2 (2.2)
Deciduoid	-	1 (1.1)
No histology obtained	1 (25)	6 (6.6)
**Brims prognostic score**		
1 (best prognosis)	2 (50)	11 (12.1)
2	1 (25)	33 (36.3)
3	1 (25)	16 (17.6)
4 (worst prognosis)	-	31 (34.1)

### Acceptability of TwiC design

The TwiC methodology was considered acceptable by TILT participants and their family members when it was described to them during qualitative interviews. Participants’ and relatives’ views varied as to whether the TwiC methodology was preferable to a blinded, placebo-controlled trial, but overall, it was considered an acceptable approach. Participants were happy with the frequency of trial visits:

"I thought the more I see you, the better I am going to be, was my sort of idea." Participant 104-1T, 61-year-old male.

Participants did not describe any problems with completing the trial paperwork, including the patient-reported outcome measures:

"Yeah, you know, those little tick sheets take half a minute, don’t they? It is not like you are having to write a ten-page essay every day, no, it is nothing." Participant 32-6T, 71-year-old male.

Overall, participants were positive about their experience of participating in TILT and did not suggest any changes or modifications if the trial was to be replicated on a larger scale:

"I have no suggestions [for improvement], no, I think it was all good stuff" Participant 33-9C, 84-year-old male.

### Secondary outcomes — adverse events

There were three serious adverse events (SAE), affecting one person in each study arm. There were no adverse events of grades 4 or 5 severity and no deaths during the trial (Table 3). All three participants who received an IMP experienced a systemic inflammatory response syndrome, consisting of pyrexia, malaise, increased breathlessness and fatigue, with corresponding increases in serum CRP and platelets. Symptoms began within 72 h of IMP administration and resulted in admission to hospital for two people. Simple analgesia and anti-inflammatory medication resolved symptoms in two patients, but one required a course of oral steroids due to symptom persistence. In response to the first two adverse reactions, the DMC passed an USM recommending a lower dose of BCG and OK432.

**Table 3:** Adverse events according to treatment allocation

	**OK432**	**BCG**	**Control**
**Number of participants**	1	3	3
**Any adverse event**	1	3	4
Grade 1	-	-	2
Grade 2	-	1	1
Grade 3	1	2	1
Grade 4/5	-	-	-
**Serious adverse event**	1	1	1
**Specific events**			
Systemic inflammatory response	1	3	-
Pleural infection	-	-	2
Chest wall pain	-	-	1
Upper respiratory tract infection	-	-	1

One control participant developed pleural infection and required admission to hospital for intravenous antibiotics.

### Exploratory efficacy outcomes

No partial or complete radiological responses were seen. Three participants (42.9%) had progressive disease and four stable disease (57.1%), with no difference in radiological outcomes between groups (3 people with stable disease in IMP group vs 1 in control).

When survival status was reviewed, four patients were alive, and three had died (median follow-up for living patients 25.0 months, range 8.9–45.0). Median survival was 21.0 months (*IQR* 8.9–29.0) with a 1-year survival rate of 71.4% (5/7). There was no difference in survival between participants randomised to receive OK432 or BCG (18.1 months; *IQR* 12.1–23.3) and control participants (29.0 months; *IQR* 5.2–45.0) with an unadjusted HR of 2.1 (95% CI 0.2-24.5; *p*=0.56) and an adjusted HR of 1.7 (95% CI 0.1-31.0; *p*=0.73).

IPC drainage volumes ranged from 0 to 1500 ml (mean 436.7 ml per drainage). Participants randomised to receive OK432 or BCG experienced a steady decline in IPC drainage volume over the trial period, whilst control participants’ drainage volumes remained stable (see Appendix C).

Six participants (85.7%) achieved pleurodesis, and all had their IPCs removed. Median time from randomisation to pleurodesis was 42 days (*IQR* 30–132 days). People randomised to receive an IMP were no more likely to achieve pleurodesis than control participants (*IMP*: 3/4; 75% vs control: 3/3; 100%), and there was no difference in time to pleurodesis between groups (HR 0.35; 95% CI 0.06-2.13; *p*=0.26) (*IMP*: median 87 days, *IQR* 36–262 vs controls: median 41 days, *IQR* 5–92).

### Patient-reported outcomes

Overall, TILT participants rated their symptoms as low severity and their QoL reasonably high. Breathlessness was the most troublesome symptom, with a median VAS score of 18.3 (range 0–36, *IQR* 8.3–25). Chest pain (median 4.7, range 0–11.2, IQR 1.5–11.2) and sweats (median 2.2, range 0–14.5, *IQR* 0.3–7.9) were less severe. Median QoL score was 80 (range 66.7–90, *IQR* 76.9–81.7), where 0 was the worst health imaginable and 100 the best.

There was no difference between the trial arms in patient-reported symptom scores at each visit, nor was there any difference in change in symptom scores over time between groups Appendix D).

## Discussion

As the first CTIMP to use the TwiC methodology, TILT demonstrated that the requisite approvals could be obtained from the Research Ethics Committee, the HRA and the MHRA. Additionally, the trial design and processes were acceptable to participants and their relatives. However, the TwiC methodology was not feasible for this trial of intra-pleural immunotherapy in people with MPM, as TILT failed to meet prespecified recruitment and retention targets. In part, this was due to specific elements of the TwiC design, including attempts to maintain blinding of control participants, although fewer eligible patients than anticipated were another contributing factor. Importantly for future CTIMP TwiCs, post-randomisation attrition was an issue, which could cause bias if it occurred in a full-scale trial.

Theoretically, screening and recruiting to a TwiC from an existing cohort of research-active patients should expedite recruitment, and previous TwiCs have certainly benefited from this aspect of the trial design [38]. However, the recruitment challenges experienced during TILT outweighed the potential recruitment benefits usually associated with the TwiC design [26]. This can be explained by the uneven geographic distribution of mesothelioma around the country and the fact that clinical trials tend to be run from regional tertiary referral centres. It is common practice for patients to be referred to these centres specifically for consideration of clinical trials, with trials often publicised at clinical and academic meetings around the UK, as well as directly to patients via resources such as the MesotheliomaUK clinical trials spreadsheet and Cancer Research UK’s clinical trials webpage. Unfortunately, this approach was not possible with the TwiC design; as before referring a patient to a tertiary centre, clinicians must discuss the trial with their patients and enquire whether they are willing to travel to the trial centre. This would have undermined blinding of potential control participants and negated one of the key elements of the TwiC design. The inability to advertise the trial and invite participants from other centres impacted on recruitment to TILT and would likely affect future mesothelioma TwiCs similarly.

Recruitment was also affected by the small number of mesothelioma patients who met the eligibility criteria — just seven people of 43 screened. This was mainly due to fewer patients with IPCs in situ and with non-loculated effusions and expandable lung than originally anticipated. When TILT was designed, the prevalence of NEL in MPM was not known; however, a paper was published subsequently that reported NEL in 64 of 192 people with pleural effusions, with just 55% (128/229) of all newly diagnosed mesothelioma patients having a pleural effusion with expandable lung at presentation [39]. The prevalence of loculated effusions remains unknown. These factors are likely to limit future trials of intra-pleural immunotherapy as, in the absence of expandable lung, the pro-inflammatory effects of intra-pleural bacteria would lead to the formation of multiple septations within the fluid, creating a complicated, multiloculated effusion. This phenomenon was observed in one TILT participant and resulted in a persistent pleural collection that was challenging to drain and caused ongoing respiratory symptoms.

A functioning IPC was required to enable out-patient delivery of intra-pleural immunotherapy. However, another constraint to recruitment was the high number of people whose effusion was too small or had auto-pleurodesed prior to IPC insertion. IPCs can be inserted in patients with minimal or no effusion via surgical techniques and this approach that has been employed previously in intra-pleural therapy trials [40–42]. However, a significant proportion of people with mesothelioma will be unsuitable to undergo an invasive procedure under general anaesthetic, so in reality, this is unlikely to increase the number of eligible patients substantially. The emergence of IPC management methods that prioritise pleurodesis, e.g. daily drainage regimens and talc slurry delivery via IPCs, will further reduce the number of people with a functioning IPC in situ and create additional recruitment limitations for a full-scale trial of intra-pleural bacterial immunotherapy in MPM [43, 44]. Future intra-pleural therapy trials are likely to require a large number of sites, with large mesothelioma case loads and high numbers of IPC insertions.

A stated benefit of the TwiC design is that participants in the cohort study can provide control data for the trial. However, to be eligible to undergo randomisation for TILT (whether to IMP or control arm), cohort participants were required to meet the trial inclusion criteria, which limited the eligible population to seven. In the absence of specific trial eligibility criteria, participants may have been randomly selected to receive an IMP without having the required access or underlying effusion characteristics to allow the drug to be given safely. An alternative approach could have been to randomise all seven eligible participants to either BCG or OK432 and to select matched control participants from the cohort. However, this could not be considered a truly randomised trial, and there would have been a high risk of bias due to fundamental differences in participant and disease characteristics at baseline.

Despite efforts to maintain blinding (at the expense of recruitment), all participants in TILT were (unbeknownst to the research team) aware of the trial before they were randomly assigned in the trial. Universally, this was due to engagement in patient and public involvement activities or patient support groups. People with mesothelioma have previously been shown to be highly interested in clinical research and motivated to find out about trials [45–47], and this is a characteristic that should be supported and encouraged by clinicians and other professionals working in the field. The blinding required to run a successful TwiC in people with mesothelioma is at odds with this and renders the methodology unfeasible in this patient population. Studies planning to use the TwiC methodology in similarly engaged patient groups should be aware of this potential issue. Feasibility studies with embedded qualitative research exploring the extent and impact of unblinding, as well possible mechanisms to reduce or mitigate its effects, should be undertaken prior to full-scale trial development.

TILT also breached the threshold for attrition that was prespecified as part of the feasibility evaluation, with two participants withdrawing from the trial after randomisation. Post-randomisation attrition is more likely with a TwiC than a standard RCT, as in TwiCs participants are told about the intervention only after being randomly allocated to that arm. For patients with mesothelioma, many of whom express a desire to prioritise quality of life over possible side effects of pharmacological therapies [12]; it seems inevitable that a proportion will decline the trial intervention after having been assigned to receive it. Post-randomisation attrition could have important impact on a full-scale TwiC in mesothelioma, either rendering the trial underpowered or introducing bias if attrition was unequal between arms [48].

### Strengths and limitations

This is the first CTIMP to use the TwiC methodology, and several important methodological considerations were noted during trial set-up [30]. By conducting an initial feasibility study that replicated the intended processes for a full-scale TwiC of intra-pleural immunotherapy in MPM, TILT enabled identification of potential challenges and limitations using a fraction of the resource required by a full-scale trial. Additionally, by using a mixed methods approach with combined quantitative and qualitative data, TILT provided a comprehensive picture of the feasibility and acceptability of the trial to participants and relatives. Based on the results of TILT, a full-scale TwiC of intra-pleural bacterial immunotherapy is not advised, and any trial of intra-pleural therapies in MPM should be mindful of the eligibility issues here and adjust recruitment window and number of sites accordingly.

Although not designed to evaluate efficacy or other clinical outcomes, these measurements were likely to have been affected by the open-label nature of both trial arms. This could have introduced assessment bias, particularly in the completion of subjective assessments such as patient-reported outcome measures. To avoid this, future full-scale efficacy trials of intra-pleural bacterial immunotherapy should elect for an objective primary outcome measure, such as survival, which is less likely to be subject to this bias. Alternatively, if a patient-focussed outcome is desired, a standard double-blind approach may be preferred.

TILT participants in both trial arms had significantly longer survival times compared with national survival data for MPM [5, 49]. This highlights an issue frequently affecting MPM trials, which of selection bias, and emphasises the importance of randomised data in evaluating the effect of an intervention. The fact that control participants and people who received an IMP lived longer than expected meant that the positive outcome could not be credited to IMP efficacy, nor was it probable that the act of participating in the trial conferred a survival benefit. Instead, the people who participated in the trial likely had a better prognosis at the outset, hence their extended survival. This may have been a result of survivorship bias, as TILT participants were not required to be recently diagnosed to participate. Indeed, only two people were commenced in the trial within a month of receiving their diagnosis, the remaining participants enrolled between 3 and 30 months after diagnosis. Since several participants had already outlived the predicted 12-month life expectancy for MPM, it was not surprising, therefore, that they remained alive many months later at the end of the trial. Survivorship bias is a particular risk in post-frontline therapy trials and may explain the recent pattern of positive single-arm phase 2 trials being followed by negative phase 3 RCTs in MPM [10, 50].

As well as affecting outcomes, the selection and survivorship bias that affected TILT would limit the external validity of a full-scale trial using this methodology. The trial population was clearly not representative of the UK MPM population, and this would also be the case for a full-scale trial. This would limit the generalisability of the data and undermine one of the fundamental aims of TILT, which was to be a pragmatic trial that closely resembled real-world care, with high external validity.

In summary, TILT demonstrated that it was possible to design a CTIMP using the TwiC design and to obtain ethical and regulatory approval. However, the TwiC approach was not feasible in people with mesothelioma. Additionally, eligibility constraints rendered recruitment challenging, and this should be an important future consideration if further trials of intra-pleural immunotherapy are planned in MPM.

## Supplementary Information

**Additional file 1: Appendix A.** TILT protocol.

**Additional file 2: Appendix B.** Trial Specific Procedure for IMP administration.

**Additional file 3: Appendix C.** Average IPC drainage volumes (mean & 95% confidence intervals) at each trial visit for participants randomised to receive OK432 or BCG (IMP group) and controls.

**Additional file 4: Appendix D.** Results for patient-reported outcome measures. **Figure C1.** Patient reported symptom scores (mean and 95% confidence intervals) for breathlessness in people randomised to receive an IMP compared with controls at each study visit. **Figure C2.** Patient reported symptom scores (mean and 95% confidence intervals) for chest pain for people randomised to receive an IMP compared with controls at each study visit. **Figure C3.** Patient reported symptom scores (mean and 95% confidence intervals) for sweats in people randomised to receive an IMP compared with controls at each study visit. **Figure C4.** 1 Patient reported symptom scores (mean and 95% confidence intervals) for quality of life in people randomised to receive OK432 or BCG compared with controls at each study visit.

**Additional file 5.** PPI statement.

## Data Availability

The TILT protocol is provided as Supplementary Material, alongside the IMP administration procedure. Raw data and additional trial material are available on request from the corresponding author.

## References

[1] Beckett P, Edwards J, Fennell D, Hubbard R, Woolhouse I, Peake MD (2015). Demographics, management and survival of patients with malignant pleural mesothelioma in the National Lung Cancer Audit in England and Wales. Lung Cancer.

[2] Hodgson J, McElvenny D, Darnton A, Price M, Peto J (2005). The expected burden of mesothelioma mortality in Great Britain from 2002 to 2050. Br J Cancer.

[3] Yates D, Corrin B, Stidolph P, Browne K (1997). Malignant mesothelioma in south east England: clinicopathological experience of 272 cases. Thorax.

[4] Ribak J, Selikoff IJ (1992). Survival of asbestos insulation workers with mesothelioma. Br J Ind Med.

[5] Woolhouse I, Bishop L, Darlison L, De Fonseka D, Edey A, Edwards J (2018). British Thoracic Society guideline for the investigation and management of malignant pleural mesothelioma. Thorax.

[6] Zalcman G, Mazieres J, Margery J, Greillier L, Audigier-Valette C, Moro-Sibilot D (2016). Bevacizumab for newly diagnosed pleural mesothelioma in the Mesothelioma Avastin Cisplatin Pemetrexed Study (MAPS): a randomised, controlled, open-label, phase 3 trial. Lancet.

[7] Baas P, Scherpereel A, Nowak A, Fujimoto N, Peters S, Tsao A (2020). First-line nivolumab + ipilimumab vs chemotherapy in unresectable malignant pleural mesothelioma: CheckMate 743. J Thorac Oncol.

[8] Mansfield AS, Roden AC, Peikert T, Sheinin YM, Harrington SM, Krco CJ (2014). B7–H1 expression in malignant pleural mesothelioma is associated with sarcomatoid histology and poor prognosis. J Thorac Oncol.

[9] Cedrés S, Ponce-Aix S, Zugazagoitia J, Sansano I, Enguita A, Navarro-Mendivil A (2015). Analysis of expression of programmed cell death 1 ligand 1 (PD-L1) in malignant pleural mesothelioma (MPM). PLoS ONE.

[10] Maio M, Scherpereel A, Calabrò L, Aerts J, Perez SC, Bearz A, et al. Tremelimumab as second-line or third-line treatment in relapsed malignant mesothelioma (DETERMINE): a multicentre, international, randomised, double-blind, placebo-controlled phase 2b trial. Lancet Oncol. 2017;18:1261–73. 10.1016/S1470-2045(17)30446-1 28729154

[11] Popat S, Curioni-Fontecedro A, Dafni U, Shah R, O'Brien M, Pope A, Fisher P, et al. A multicentre randomised phase III trial comparing pembrolizumab versus single-agent chemotherapy for advanced pre-treated malignant pleural mesothelioma: the European Thoracic Oncology Platform (ETOP 9-15) PROMISE-meso trial. Ann Oncol. 2020;31(12):1734-45. 10.1016/j.annonc.2020.09.009 32976938

[12] Bibby AC, De Fonseka D, Morley AJ, Keenan E, Addeo A, Smith S (2017). Exploring the characteristics of patients with mesothelioma who chose active symptom control over chemotherapy as first-line treatment: a prospective, observational, single centre study. BMC Palliat Care.

[13] Vogelzang NJ, Rusthoven JJ, Symanowski J, Denham C, Kaukel E, Ruffie P (2003). Phase III study of pemetrexed in combination with cisplatin versus cisplatin alone in patients with malignant pleural mesothelioma. J Clin Oncol.

[14] Scherpereel A, Mazieres J, Greillier L, Lantuejoul S, Dô P, Bylicki O (2019). Nivolumab or nivolumab plus ipilimumab in patients with relapsed malignant pleural mesothelioma (IFCT-1501 MAPS2): a multicentre, open-label, randomised, non-comparative, phase 2 trial. Lancet Oncol.

[15] Watanabe M, Boyer JL, Crystal RG (2010). AAVrh. 10-mediated genetic delivery of bevacizumab to the pleura to provide local anti-VEGF to suppress growth of metastatic lung tumors. Gene Ther.

[16] Davies CWHLSDR (1998). The systemic fibrinolytic activity of intrapleural streptokinase. Am J Respir Crit Care Med.

[17] Felletti R, Ravazzoni C (1983). Intrapleural Corynebacterium parvum for malignant pleural effusions. Thorax.

[18] Millar J, Hunter A, Horne N (1980). Intrapleural immunotherapy with Corynebacterium parvum in recurrent malignant pleural effusions. Thorax.

[19] Ostrowski M, Priestman T, Houston R, Martin W (1989). A randomized trial of intracavitary bleomycin and Corynebacterium parvum in the control of malignant pleural effusions. Radiother Oncol.

[20] Bibby AC, Walker S, Maskell NA (2018). Are intra-pleural bacterial products associated with longer survival in adults with malignant pleural effusions? A systematic review. Lung Cancer.

[21] Stephens RJ, Whiting C, Cowan K (2015). Research priorities in mesothelioma: a James Lind Alliance Priority Setting Partnership. Lung Cancer.

[22] Uchida A, Micksche M (1983). Lysis of fresh human tumor cells by autologous peripheral blood lymphocytes and pleural effusion lymphocytes activated by OK432. J Natl Cancer Inst.

[23] Sakamoto J, Teramukai S, Watanabe Y, Hayata Y, Okayasu T, Nakazato H (2001). Meta-analysis of adjuvant immunochemotherapy using OK-432 in patients with resected non-small-cell lung cancer. J Immunother.

[24] Prescott S, James K, Hargreave TB, Chisholm GD, Smyth JF (1992). Intravesical Evans strain BCG therapy: quantitative immunohistochemical analysis of the immune response within the bladder wall. J Urol.

[25] Sylvester RJ, van der Meijden APM, Witjes JA, Kurth K (2005). Bacillus Calmette-Guerin versus chemotherapy for the intravesical treatment of patients with carcinoma in situ of the bladder: a meta-analysis of the published results of randomized clinical trials. J Urol.

[26] Relton C, Torgerson D, O’Cathain A, Nicholl J (2010). Rethinking pragmatic randomised controlled trials: introducing the “cohort multiple randomised controlled trial” design. BMJ.

[27] van der Velden JM, Verkooijen HM, Young-Afat DA, Burbach JP, van Vulpen M, Relton C, et al. The cohort multiple randomized controlled trial design: a valid and efficient alternative to pragmatic trials? Int J Epidemiol. 2017;46(1):96-102 10.1093/ije/dyw050 27118559

[28] Verkooijen H, Roes K, van Gils C (2013). Cohort multiple randomized controlled trial: a solution for the evaluation of multiple interventions. Ned Tijdschr Voor Geneeskd.

[29] Loudon K, Treweek S, Sullivan F, Donnan P, Thorpe KE, Zwarenstein M (2015). The PRECIS-2 tool: designing trials that are fit for purpose. British Med J..

[30] Bibby AC, Torgerson DJ, Leach S, Lewis-White H, Maskell NA (2018). Commentary: considerations for using the ‘trials within cohorts‘ design in a clinical trial of an investigational medicinal product. Trials.

[31] Kasahara K, Shibata K, Shintani H, Iwasa K-I, Sone T, Kimura H (2006). Randomized phase II trial of OK-432 in patients with malignant pleural effusion due to non-small cell lung cancer. Anticancer Res.

[32] Bakker W, Nijhuis-Heddes J, van der Velde E (1986). Post-operative intrapleural BCG in lung cancer: a 5-year follow-up report. Cancer Immunol Immunother.

[33] Mckneally M, Maver C, Kausel H (1976). Regional immunotherapy of lung cancer with intrapleural BCG. Lancet.

[34] Byrne MJ, Nowak AK (2004). Modified RECIST criteria for assessment of response in malignant pleural mesothelioma. Ann Oncol.

[35] NIHR. Pilot and feasibility studies. https://www.journalslibrarynihrac.uk/information-for-authors/pilot-and-feasibility-studies/ Accessed 29 June 2020.

[36] Clarke V, Braun V (2014). Thematic analysis. Encyclopedia of Critical Psychology: Springer.

[37] Bibby AC, Morley AJ, Keenan E, Maskell NA, Gooberman-Hill R (2022). The priorities of people with mesothelioma and their carers: a qualitative interview study of trial participation and treatment decisions. Eur J Oncol Nurs.

[38] Gal R, Monninkhof EM, van Gils CH, Groenwold RHH, van den Bongard DHJG, Peeters PHM (2019). The trials within cohorts design faced methodological advantages and disadvantages in the exercise oncology setting. J Clin Epidemiol.

[39] Bibby AC, Halford P, De Fonseka D, Morley AJ, Smith S, Maskell NA (2019). The prevalence and clinical relevance of non-expandable lung in malignant pleural mesothelioma: a prospective, single-center cohort study of 229 patients. Ann Am Thorac Soc.

[40] Sterman DH, Alley E, Stevenson JP, Friedberg J, Metzger S, Recio A (2016). Pilot and feasibility trial of immuno-gene therapy of malignant mesothelioma using intrapleural delivery of adenovirus- interferon-alpha combined with chemotherapy. Clin Cancer Res.

[41] Sterman DH, Molnar-Kimber K, Iyengar T, Chang M, Lanuti M, Amin KM (2000). A pilot study of systemic corticosteroid administration in conjunction with intrapleural adenoviral vector administration in patients with malignant pleural mesothelioma. Cancer Gene Ther.

[42] Sterman DH, Recio A, Vachani A, Sun J, Cheung L, DeLong P (2005). Long-term follow-up of patients with malignant pleural mesothelioma receiving high-dose adenovirus herpes simplex thymidine kinase/ganciclovir suicide gene therapy. Clin Cancer Res.

[43] Bhatnagar R, Keenan EK, Morley AJ, Kahan BC, Stanton AE, Haris M (2018). Outpatient talc administration by indwelling pleural catheter for malignant effusion. N Engl J Med.

[44] Wahidi MM, Reddy C, Yarmus L, Feller-Kopman D, Musani A, Shepherd RW (2017). Randomized trial of pleural fluid drainage frequency in patients with malignant pleural effusions. The ASAP trial. Am J Respir Crit Care Med.

[45] Warnock C, Lord K, Taylor B, Tod A (2019). Patient experiences of participation in a radical thoracic surgical trial: findings from the Mesothelioma and Radical Surgery Trial 2 (MARS 2). Trials.

[46] British Lung Foundation. The British Lung Foundation Survey of people affected by mesothelioma 2013. Available at https://patientperspective.org/wp-content/uploads/2014/10/BLF-mesothelioma-survey-report.pdf, Accessed 20 July 2020.

[47] Darlison L, Mckinley D, Moore S. Findings from the National mesothelioma experience survey. Cancer Nurs Prac. 2014;13(3).

[48] Pate A, Candlish J, Sperrin M, Van Staa TP, 2 oboGWP (2016). Cohort Multiple Randomised Controlled Trials (cmRCT) design: efficient but biased? A simulation study to evaluate the feasibility of the Cluster cmRCT design. BMC Med Res Methodol.

[49] Royal College of Physicians (2018). National Mesothelioma Audit report 2018 (for the audit period 2014–16).

[50] Calabro L, Morra A, Fonsatti E, Cutaia O, Amato G, Giannarelli D, et al. Tremelimumab for patients with chemotherapy-resistant advanced malignant mesothelioma: an open-label, single-arm, phase 2 trial. Lancet Oncol. 2013;14(11):1104–11. 10.1016/S1470-2045(13)70381-4 24035405

