# Supplementary material for: A trial of intra-pleural bacterial immunotherapy in malignant pleural mesothelioma (TILT) — a randomised feasibility study using the trial within a cohort (TwiC) methodology
Source: Pilot Feasibility Stud. 2022 Sep 3;8:196. doi: 10.1186/s40814-022-01156-3 (PMC9440504; doi:10.1186/s40814-022-01156-3)
Supplement: Supplementary file 1 — Additional file 1: Appendix A. TILT protocol. [file 40814_2022_1156_MOESM1_ESM.docx]

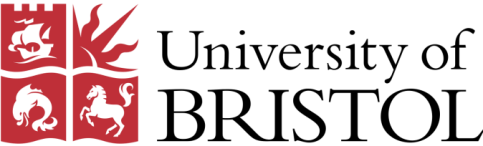


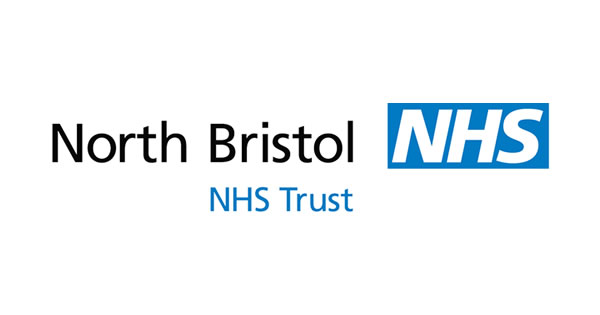


A **T**rial of **I**ntra-pleura**L** bacterial immuno-**T**herapy in mesothelioma

A feasibility study using the ‘trial within a cohort’ methodology

**Chief Investigator: Professor Nick Maskell**

**Principal Investigator (Bristol): Dr Anna Bibby**

**Principal Investigator (Oxford): Professor Najib Rahman**

**Principal Investigator (Taunton): Dr Janet Fallon**

Protocol Version 3.11

Version date 07/03/19

R&I number 3850

IRAS project ID 215394

EudraCT Number 2016-004727-23

ISRCTN 10432197

Authorised by:


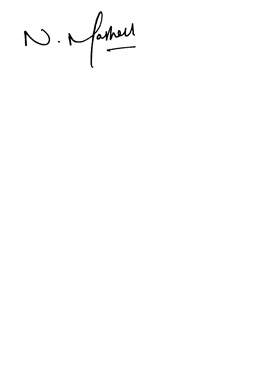


Dr Nick Maskell

07/03/19

**GENERAL INFORMATION**

This document describes the TILT trial and provides information about procedures for entering patients into it. The protocol should not be used as an aide-memoire or guide for the treatment of other patients. Every care was taken in the creation of this document, but corrections or amendments may be necessary.

**COMPLIANCE**

The trial will be conducted in compliance with the protocol, Research Governance Framework, Data Protection Act and other guidelines as appropriate.

**FUNDING SOURCE**

This trial has been funded by a National Institute of Health Research (NIHR) Doctoral Research Fellowship (DRF) awarded to Dr Anna Bibby (reference DRF-2016-09-065).

**RESEARCH TEAM CONTACTS**

Clinical queries should be directed to the local principal investigator, or to the trial co-ordinator or chief investigator

For general queries, supply of trial documentation and collection of data, please contact the trial co-ordinator.

**Sponsor: North Bristol NHS Trust**

**Research and Innovation**

2^nd^ Floor L&R Building

Southmead Hospital

Bristol Tel: 0117 414 9330

BS10 5NB Email:

[Researchsponsor@nbt.nhs.uk](mailto:Researchsponsor@nbt.nhs.uk)

**Chief Investigator:** **Prof. Nick Maskell**

Professor of Respiratory Medicine

Academic Respiratory Unit

University of Bristol

2^nd^ Floor L&R Building

Southmead Hospital

Bristol Tel: 0117 414 6337

BS10 5NB Email: [nick.maskell@bristol.ac.uk](mailto:nick.maskell@bristol.ac.uk)

**Principal Investigator: Dr Anna Bibby**

**(Bristol)** NIHR Doctoral Research Fellow

Academic Respiratory Unit

University of Bristol

2^nd^ Floor L&R Building

Southmead Hospital

Bristol Tel: 0117 414 8049

BS10 5NB Email: [anna.bibby@bristol.ac.uk](mailto:anna.bibby@bristol.ac.uk)

**Principal Investigator: Prof. Najib Rahman**

**(Oxford)** Associate Professor and Respiratory Consultant

Oxford Centre for Respiratory Medicine

Churchill Hospital

Oxford Tel: 01865 225205

OX3 7LJ Email: [najib.rahman@ndm.ox.ac.uk](mailto:najib.rahman@ndm.ox.ac.uk)

**Trial Coordinator:** **Dr Emma Keenan**

Respiratory Research Unit

Clinical Research Centre

Southmead Hospital

Bristol Tel: 0117 414 8114

BS10 5NB Email: [emma.keenan@nbt.nhs.uk](mailto:emma.keenan@nbt.nhs.uk)

Emergency contact **Dr Anna Bibby**

NIHR Doctoral Research Fellow

Academic Respiratory Unit

University of Bristol

2^nd^ Floor L&R Building

Southmead Hospital

Bristol Tel: 07813077353

BS10 5NB

**RESEARCH COLLABORATORS**

**Qualitative collaborator: Prof. Rachael Gooberman-Hill**

Professor of Health and Anthropology

Musculoskeletal Research Unit

University of Bristol

Level 1 L&R Building

Southmead Hospital

Bristol Tel: 0117 414 7845

BS10 5NB Email: [r.gooberman-hill@bristol.ac.uk](mailto:r.gooberman-hill@bristol.ac.uk)

**York Trials Unit Collaborator: Prof. David Torgerson**

Director, York Clinical Trials Unit

Department of Health Sciences

University of York

Seebohm Rowntree Building

Heslington

York Tel: 01904 321340

YO10 5DD Email: [david.torgerson@york.ac.uk](mailto:david.torgerson@york.ac.uk)

Contents

[ABBREVIATIONS 8](#_Toc508023852)

[1. TRIAL OVERVIEW 9](#_Toc508023853)

[1.1 Plain English summary 9](#_Toc508023854)

[1.2 Abstract 10](#_Toc508023855)

[1.3 Study design 11](#_Toc508023856)

[1.4 Trial flowchart 12](#_Toc508023857)

[2. BACKGROUND 14](#_Toc508023858)

[2.1 Mesothelioma 14](#_Toc508023859)

[2.2 Bacterial immunotherapy 14](#_Toc508023860)

[2.3 OK432 14](#_Toc508023861)

[2.4 BCG 15](#_Toc508023862)

[2.5 The trial within a cohort (TwiC) methodology 16](#_Toc508023863)

[3. AIMS & OBJECTIVES 17](#_Toc508023864)

[5. MESOTHELIOMA COHORT STUDY (ASSESS-meso) 17](#_Toc508023865)

[5.1 Setting 17](#_Toc508023866)

[5.2 Inclusion criteria (ASSESS-meso) 17](#_Toc508023867)

[5.3 Exclusion criteria (ASSESS_meso) 18](#_Toc508023868)

[5.4 TwiC specific consent within ASSESS-meso 18](#_Toc508023869)

[5.5 TILT and ASSESS-meso related activities 18](#_Toc508023870)

[6. TRIAL METHODOLOGY 19](#_Toc508023871)

[6.1 Trial setting 19](#_Toc508023872)

[6.2 Inclusion criteria 19](#_Toc508023873)

[6.3 Exclusion criteria 19](#_Toc508023874)

[6.4 Random selection 19](#_Toc508023875)

[6.5 Consent 20](#_Toc508023876)

[6.6 Sample size & recruitment targets 21](#_Toc508023877)

[6.7 Co-enrolment guidelines 21](#_Toc508023878)

[7. TRIAL TREATMENTS 22](#_Toc508023879)

[7.1 Arm 1 -OK432 22](#_Toc508023880)

[7.2 Arm 2 – BCG 22](#_Toc508023881)

[7.3 Intervention visit 23](#_Toc508023882)

[7.4 Storage, dispensing and packaging of OK432 23](#_Toc508023883)

[7.5 Storage, dispensing and packaging of BCG 23](#_Toc508023884)

[7.6 Accountability & unused drugs 24](#_Toc508023885)

[8. NON-TRIAL TREATMENTS 24](#_Toc508023886)

[8.1 Usual care 24](#_Toc508023887)

[8.2 Indwelling pleural catheters 24](#_Toc508023888)

[8.2.1 IPC insertion 24](#_Toc508023889)

[8.2.2 IPC drainage 24](#_Toc508023890)

[8.2.3 IPC blockage 25](#_Toc508023891)

[8.2.4 Assessment of pleurodesis 25](#_Toc508023892)

[8.2.5 IPC removal 25](#_Toc508023893)

[8.3 Permitted medications 25](#_Toc508023894)

[8.4 Non-permitted medications 26](#_Toc508023895)

[8.5 Permitted interventions 26](#_Toc508023896)

[8.6 Non-permitted interventions 26](#_Toc508023897)

[8.7 Data on concomitant medications and interventions 26](#_Toc508023898)

[9. TRIAL FOLLOW UP ASSESSMENTS 27](#_Toc508023899)

[9.1 Trial visits 27](#_Toc508023900)

[9.2 Feasibility assessments 27](#_Toc508023901)

[9.3 Efficacy assessments 27](#_Toc508023902)

[9.4 Safety assessments 28](#_Toc508023903)

[9.5 Source data 28](#_Toc508023904)

[9.6 End of trial (EoT) 28](#_Toc508023905)

[9.7 Schedule of visits 29](#_Toc508023906)

[10. QUALITATIVE RESEARCH 30](#_Toc508023907)

[10.1 Existing qualitative literature in MPM 30](#_Toc508023908)

[10.2 Aim of qualitative research 30](#_Toc508023909)

[10.3 Methodology for in-depth interviews 30](#_Toc508023910)

[10.4 Methodology for focus group sessions 32](#_Toc508023911)

[10.5 Qualitative interviews with bereaved relatives 33](#_Toc508023912)

[10.6 Sample size for qualitative research 33](#_Toc508023913)

[11. PARTICIPANT WITHDRAWAL/NON-CONSENT 34](#_Toc508023914)

[11.1 Withdrawal of consent to all trial involvement 34](#_Toc508023915)

[11.2 Withdrawal of consent for further data collection 34](#_Toc508023916)

[11.3 Withdrawal of consent for further data collection and use of existing data 34](#_Toc508023917)

[11.4 Withdrawal of consent for sample analysis 34](#_Toc508023918)

[11.5 Loss to follow up 34](#_Toc508023919)

[12. ADVERSE EVENT MONITORING 35](#_Toc508023920)

[12.1 Definitions 35](#_Toc508023921)

[12.2 Expectedness 35](#_Toc508023922)

[12.3 Causality 36](#_Toc508023923)

[12.4 Adverse events in TILT and ASSESS-meso participants 36](#_Toc508023924)

[12.5 Reporting procedures 37](#_Toc508023925)

[12.6 Follow-up after adverse events 38](#_Toc508023926)

[12.7 Outcome grading of adverse events 38](#_Toc508023927)

[12.8 Contact details for adverse event reporting 38](#_Toc508023928)

[12.9 Reference Safety Information 39](#_Toc508023929)

[12.10 Development Safety Update Report (DSUR) 39](#_Toc508023930)

[13. STATISTICAL ANALYSIS 39](#_Toc508023931)

[13.1 Primary outcome measure 39](#_Toc508023932)

[13.2 Secondary outcome measures 39](#_Toc508023933)

[13.3 Analysis plan 40](#_Toc508023934)

[14. ETHICAL CONSIDERATIONS 41](#_Toc508023935)

[14.1 Indwelling pleural catheters 41](#_Toc508023936)

[14.2 OK432 41](#_Toc508023937)

[14.3 BCG 41](#_Toc508023938)

[14.4 Qualitative interviews 41](#_Toc508023939)

[14.5 Focus group sessions 42](#_Toc508023940)

[14.6 Qualitative interviews with bereaved relatives 42](#_Toc508023941)

[14.7 Consent and withdrawal 43](#_Toc508023942)

[14.8 Data Management 43](#_Toc508023943)

[14.9 Confidentiality 44](#_Toc508023944)

[14.10 Data security 44](#_Toc508023945)

[14.11 Data sharing 44](#_Toc508023946)

[14.12 Ethical approval 45](#_Toc508023947)

[15. REGULATORY APPROVAL 45](#_Toc508023948)

[15.1 MHRA approval 45](#_Toc508023949)

[15.2 NIHR portfolio registration 45](#_Toc508023950)

[15.3 EudraCT registration 45](#_Toc508023951)

[15.4 Research Governance Statement 45](#_Toc508023952)

[This study will be conducted in accordance with: 45](#_Toc508023953)

[• The Medicines for Human Use (Clinical Trials) Regulations 2004 and subsequent amendments 45](#_Toc508023954)

[• International Conference for Harmonisation of Good Clinical Practice (ICH GCP) guidelines. 45](#_Toc508023955)

[16. TRIAL INFRASTRUCTURE 46](#_Toc508023956)

[16.1 Monitoring and Quality control 46](#_Toc508023957)

[16.2 Trial management group 46](#_Toc508023958)

[16.3 Trial steering committee (TSC) 46](#_Toc508023959)

[16.4 Data monitoring committee 47](#_Toc508023960)

[17. FUNDING & INDEMNITY 47](#_Toc508023961)

[17.1 Funding 47](#_Toc508023962)

[17.2 Indemnity 47](#_Toc508023963)

[18. REFERENCES 48](#_Toc508023964)

# ABBREVIATIONS

°C Degrees centigrade

AE Adverse event

AR Adverse reaction

BCG Bacille Calmette Guerin

CI Chief Investigator

CRP C reactive protein

CT Computed tomography

CXR Chest radiograph

DMP Data Management Plan

ECOG Eastern Co-operative Oncology Group

EoT End of trial

EQ-5D-5L EuroQol 5D health questionnaire

eCRF Electronic case report form

FBC Full blood count

GCP Good Clinical Practice

GMC General Medical Council

GP General Practitioner

IMP Investigational medicinal product

IPC Indwelling pleural catheter

LFT Liver function tests

MDT Multidisciplinary team

MHRA Medicines and Healthcare Products Regulatory Agency

Ml Millilitres

MPE Malignant pleural effusion

MPM Malignant pleural mesothelioma

NBT North Bristol NHS Trust

NHS National Health Service

OS Overall survival

PI Principal Investigator

PFSR Progression-free survival rates

PROMs Patient-reported outcome measures

PS Performance status

QoL Quality of life

R&I North Bristol NHS Trust Research & Innovation Department

RCT Randomised controlled trial

REC Research Ethics Committee

RSI Reference Safety Information

SAE Serious adverse event

SAR Serious adverse reaction

SmPC Summary of Product Characteristics

SUSAR Suspected unexpected serious adverse reaction

TSC Trial steering committee

TSP Trial specific procedure

TUS Thoracic ultrasound scan

TWIC Trial within a cohort

U&E Urea and electrolytes

VAS Visual analogue scale

WHO World Health Organisation

YTU York Trials Unit, a UKCRC-registered, NIHR Clinical Trial Unit

# TRIAL OVERVIEW

## Plain English summary

Mesothelioma is an aggressive cancer that affects the outside lining of the lung, called the pleura. It is incurable, and there is only one effective chemotherapy treatment available. This chemotherapy extends life by just three months on average. It is crucial that we find new treatment options that can be offered to people with this disease.

There are lots of new treatments being developed for other cancers that focus on the immune system. A healthy immune system is able to identify and attack cancer cells within the body. However, mesothelioma hides from the immune system by reducing the number of protective immune cells in and around the pleura. People with mesothelioma who can overcome this and maintain lots of immune cells in the lung lining seem to live longer.

We have noticed that patients with mesothelioma live longer after having an infection in the pleura. We think this is because the infection “wakes up” the immune system that was suppressed by the mesothelioma. Immune cells move to the lung lining to attack the infection, and are able to attack the mesothelioma at the same time.

Our research aims to use two different bacterial agents to mimic infection and stimulate the immune system to attack the mesothelioma. The two bacteria are OK432, an inactivated, “dead” bacteria and BCG, a weakened version of the tuberculosis bacteria, currently used as a vaccine. We want to investigate whether either of these bacterial agents could help people live longer with mesothelioma. We will use a research design that aims to copy real life clinical care (called the ‘trial within a cohort’ design), to see whether it can be used in larger mesothelioma trials.

We are currently running a study called ASSESS-meso in which patients with mesothelioma undergo regular assessments, blood tests and CT scans. Everyone in ASSESS-meso has agreed for their information to be used to identify whether they are suitable for other research trials, and to be chosen on a random basis to be invited to join those trials. From ASSESS-meso we will identify 24 people who are suitable to join TILT, and will randomly choose 16 of those people to receive either OK432 or BCG. These people will receive a single dose of OK432 or BCG, delivered directly into the pleura via an indwelling catheter. The rest of the participants in ASSESS-meso won’t be told about the trial or the bacterial agents, because in real-life, doctors only tell patients about treatments if and when they are going to receive them, and not if they are not going to receive them. This prevents people becoming disappointed or disheartened with their treatment.

All participants will be followed up with the same tests. Participants who are not selected to receive OK432 or BCG will act as a comparison group for the people who do, having given their consent for their information to be used in this way when they first joined ASSESS-meso. At the end of the TILT trial we will assess various practical issues relating to the trial, e.g. how long it took to recruit participants, how many people agreed to receive OK432 or BCG and how many people completed the trial. At the end of the trial we will also interview some of the participants and their relatives to ask what it was like to participate in the trial, whether they had any problems with it, and what could be done differently in future. We hope to gain insight into people’s experiences of the trial that will help us make future trials acceptable and attractive to participants.

## Abstract

*Background*

Malignant pleural mesothelioma (MPM) is an aggressive cancer of the lung lining. It is incurable, with median survival less than 1 year from diagnosis^1-7^. Chemotherapy extends survival by 2.8 months, but is not suitable for everyone^1 2 4^. The UK has the highest mortality rates for MPM worldwide, with 2535 people dying from it in 2012^2^. Incidence is rising, and is predicted to peak in the next decade^1-3^.

MPM is an attractive target for immunotherapy as the tumour evades the immune response that usually inhibits tumour growth^8-11^. MPM suppresses populations of protective T cells in the pleura^10-13^. The ability to overcome this is associated with longer survival^12-15^, and drugs that stimulate T cell activity are currently undergoing clinical trials^16-18^.

T cells are stimulated by bacteria. Observational studies report enhanced survival in patients with lung cancer and pleural infection^19-22^. A similar phenomenon has been reported in a series of patients with pleural infection and malignant effusions, in which patients with MPM and pleural infection lived over a year longer than non-infected patients^23^. Historically, intra-pleural bacteria have been used as pleurodesis agents, with some demonstrating concomitant, anti-tumour activity^24-34^. Attenuated bacterial agents have been shown to control pleural fluid and prolong survival in malignant pleural effusions secondary to lung cancer^35-39^. In MPM, mice injected with staphylococcus enterotoxin demonstrated florid T cell responses in association with reduced tumour growth^40^.

This research will investigate two bacterial immunotherapy agents, OK432 and BCG. OK432 is a penicillin and heat-treated, lyophilized mixture of group A Streptococcus pyogenes that induced lymphocyte-mediated tumour killing in pleural fluid in vitro, controlled malignant pleural effusions in vivo and improved survival in lung cancer^35-37^. BCG, an attenuated, low-virulence mycobacterial strain has been used intra-thoracically following resection of lung tumours and is used intravesically in non-invasive bladder cancer [refs]

*Study design*

This project will investigate intra-pleural bacterial immunotherapy in MPM using an innovative trial design, the Trial within a Cohort (TwiC)^41^. Participants will be recruited from an existing observational cohort, consisting of regular clinical and radiological monitoring (the ASSESS-meso study). From ASSESS-meso, 8 eligible participants will be randomly selected for either the OK432 or BCG arm of the trial. OK432 or BCG will be delivered intra-pleurally as a single dose via an indwelling catheter. Outcome data will be compared with control participants from ASSESS-meso. Qualitative interview will be undertaken to assess acceptability of the methodology to participants.

*Aims*

The aims of this research are:

- To assess the feasibility and acceptability of the TwiC methodology
- To explore the role of intra-pleural bacterial immunotherapy in people with MPM
- To answer the question “Is it feasible to undertake a three-armed TwiC of intra-pleural immunotherapy in MPM and is it acceptable to participants and relatives?”

If feasibility is demonstrated, results will inform the design of a full-scale TwiC of intra-pleural bacterial immunotherapy using one or both agents from this study, depending on results.

## Study design

This is a multi-centre feasibility study for a single-blind, randomised, three-armed trial of intra-pleural bacterial immunotherapy in people with mesothelioma, based on the trial within a cohort design.

Patients with mesothelioma will be invited to participate in a longitudinal observational cohort study, consisting of pleural fluid monitoring, patient-reported outcome measurements, chest radiography (CXR) and blood tests (the ASSESS-meso study). Eligible patients will be identified from the cohort and randomly allocated to receive a single dose of either intra-pleural OK432 or intra-pleural BCG, or to remain in the cohort as a control. Identification of eligible participants will continue until 8 participants have been randomly selected to receive OK432 or BCG. Randomisation will be undertaken by a centralised, concealed computerised system with minimisation by tumour sub-type and WHO/ECOG performance status (PS). Participants selected to receive bacterial immunotherapy will receive it via an indwelling pleural catheter (IPC), on an unblinded basis. Participants who decline to receive OK432 or BCG following random selection will continue to undergo observational follow up, and their data will be collected as part of the feasibility outcomes.

Participants who are not selected to receive bacterial immunotherapy will continue longitudinal assessments as part of ASSESS-meso, blinded to the existence of the OK432 and BCG arms. This is consistent with real-life clinical care, wherein patients are told about treatments as and when they are going to receive them.

The primary outcome measure is feasibility. The trial will be deemed feasible if the following criteria are met:

- Recruitment rates to time & target >66%, i.e. 8 participants randomised in 12 month recruitment window, or 12 participants in 18 months.
- Attrition rate of <20% (nb this relates solely to attrition due to loss to follow up or participant withdrawal, it does not include attrition due to participant death)
- Data completeness rates >90%

In addition, data will be collected on specific elements of the TwiC methodology to explore the acceptability of the trial design to participants. These will include:

- Acceptance rates for intra-pleural OK432/BCG following random selection
- Number of control participants who were aware of the intervention arm
- Collection of data on participants who decline any element of the trial
- Qualitative interviews with participants and their relatives to explore the acceptability of trial processes.

Secondary outcome measures will collect exploratory data on the clinical efficacy of OK432 and BCG. They will include:

- Tumour response, based on CT appearances at baseline and 3 months, assessed using modified RECIST^42^
- Overall survival (OS), defined as time between date of diagnosis with MPM to date of death, censored at the end of the trial (EoT)
- Progression-free survival rates (PFSR) at 3 months
- Patient-reported chest pain and breathlessness, measured on visual analogue scales (VAS)
- Patient-reported quality of life, measured using the EQ-5D-5L health questionnaire
- Pleurodesis rates, defined as pleural fluid drainage of less than 50ml on 3 consecutive occasions, with <25% opacification on CXR or <250ml pleural fluid on thoracic ultrasound scanning (TUS)
- Adverse events
- Biomarker response assessed using serial serum mesothelin levels.

Following completion of the trial, participants will return to follow up within ASSESS-meso cohort study until death or withdrawal from the cohort. This will yield additional data over time, and consequently a priori analyses are planned at 1 year and 2 years post-completion of TILT (i.e. following the final visit of the final participant) to evaluate overall survival, progression-free survival, adverse events and other long-term clinical outcomes.

## Trial flowchart

See page 10 for the trial flowchart.

**BCG**

**N=4**

Intra-pleural BCG

-

**OK432**

**N=4**

**TILT Trial**

Y

Consent to receive BCG/OK432

**TILT Trial**

-

Y

Intra-pleural OK432

Randomisation

1:1:1

N

**Follow up 2**

Week 6

**Random selection**

Week 0

Week 1

**Intervention**

Week 0-2

Becomes eligible for TILT

N

**Screening**

During cohort visits

**Final visit**

Week 12

**ASSESS-meso cohort**

End of cohort follow up

**Follow up 1**

3 days post intervention (or week 3 for controls)

**Control**

**N=4**

**ASSESS-meso cohort**

# BACKGROUND

## Mesothelioma

Malignant pleural mesothelioma (MPM) is an aggressive tumour of the pleura with an increasing incidence globally and in the UK.^1-3^ The UK mortality rate for MPM is the highest in the world, with one person dying from the disease every 4 hours.^1-3^

The average survival with MPM is between 9 and 14 months from diagnosis.^1-7^ Treatment options are limited, with only one chemotherapy regimen available, offering a median survival benefit of 12 weeks.^4^ Recently, the addition of Bevacizumab to this regimen extended median survival to 18 months.^43^ Unfortunately, only 30% of patients with MPM receive chemotherapy, as co-morbidities and patient choice preclude its use in others.^2^

One of the reasons that MPM is so aggressive is that it evades immunological defences that usually inhibit tumour growth. Cytotoxic T Lymphocytes and Natural Killer cells, which usually respond to tumour antigens and limit tumour progression, are depleted in the pleura of patients with MPM.^8-11^ The ability to maintain intra-pleural T cell activity is associated with longer survival.^12-15^ Drugs that stimulate T cells have shown promise in MPM, with phase 3 trials underway.^16 17 44^

## Bacterial immunotherapy

Pleural infection potently stimulates intra-pleural T cells. Anecdotal evidence suggests enhanced survival in patients with malignancy and pleural infection.^19-22^ This phenomenon has also been observed in patients with indwelling catheters for pleural malignancy.^23^

Intra-pleural bacterial agents, such as BCG and Corynebacterium parvum, have been used to treat malignant pleural effusions since the 1950s.^24-34 45^ Historically the field was controversial, but survival benefits were reported by some.^24 25 33^ Recently, in a randomised controlled trial, participants with malignant pleural effusions secondary to lung cancer demonstrated a 3 month survival benefit when treated with intra-pleural LC9018 (heat-treated Lactobacillus casei) and doxyrubicin compared with patients who received intra-pleural doxyrubicin alone.^38^ Similarly, intra-pleural Staphylococcus enterotoxin improved median survival by 5 months in 14 patients with non-small cell lung cancer.^39^

In MPM, mice injected with Staphylococcus enterotoxin showed reduced tumour growth, higher numbers of activated T cells and more extensive tumour necrosis compared with saline-injected controls.^40^ Our team has observed enhanced survival in patients with MPM and pleural infection. These early studies suggest intra-pleural bacterial agents may be beneficial in treating MPM.^23^

## OK432

One arm of this trial will use the bacterial agent OK432, a heat- and penicillin-killed lyophilised preparation of streptococcus pyogenes that has been shown to activate anti-tumour cytokines, induce dendritic cell maturation and activate T cells via Toll-like receptor-4.^46^ OK432 will be infused intra-pleurally via an indwelling pleural catheter.

In vitro incubation of lymphocytes from cancer patients with OK432 induced lymphocyte-mediated tumour killing and augmented tumour-specific cytotoxicity.^35^ In vivo, intra-pleural OK432 controlled malignant pleural effusions and is used routinely in the management of malignant pleural effusions in Japan.^36^ A systematic review of intra-venous OK432 in patients who have undergone surgical resection of lung cancer demonstrated an overall survival benefit with this treatment.^37^

Adverse events from OK432 are usually mild. Adverse reaction reports received prior to 1982 documented fever and inflammation at the injection site as the most common side effects from OK432 treatment. These occurred in 23% and 15% respectively, of 26,027 sequential patients treated with OK432.

## BCG

The other arm of the trial will use BCG, a live attenuated, low-virulence strain of mycobacterium Bovis, currently used as a vaccine against tuberculosis. BCG is known to stimulate CD4+ and CD8+ cytotoxic T lymphocytes via toll-like receptors 2 and 4, as well as eliciting the production of inflammatory (and anti-cancer) cytokines such as interferon-gamma and tumour necrosis factor.^47 48^ In addition, BCG produces a delayed type IV hypersensitivity reaction, mediated by T helper cells, which allows sustained anti-tumour activity alongside the acute inflammatory response.^49^ Finally, BCG also exerts an effect via the innate immune system, with animal models showing that natural killer cells are implicated in tumour cell killing.^50 51^

BCG has been shown to have anti-tumour activity in skin cancer and bladder cancer, and has been used as an intra-cavity anti-cancer agent in bladder cancer since 1976.^52^ Meta-analysis of data from randomised clinical trials has shown that intra-vesical BCG reduced the risk of progression in non-muscle invasive bladder cancer compared with no treatment or other intra-vesical therapies, and as a result it is now standard of care in these patients.^53-57^ Immunohistological analyses of bladder biopsies taken from patients treated with intra-vesical BCG demonstrated universal induction of major histocompatibility complexes (MHC) on antigen presenting cells and macrophages, with associated T cell-predominant inflammation of the bladder up to 6 months after treatment.^58^

In malignant melanoma, BCG injected directly into the tumour lesion was associated with regression of up to 90% of the injected tumour, and an abscopal effect of over 15% tumour reduction in non-treated lesions.^59^ This occurred in conjunction with significant prolongation of disease-free periods, and longer overall survival, even in patients with metastatic disease. The use of BCG in melanoma has been superseded in recent years by the development of designer immunotherapy drugs such as checkpoint inhibitors.^60-62^ These agents have revolutionised the treatment of melanoma, and there is growing interest in their potential as therapeutic agents for mesothelioma.^18 63^

Intra-pleural BCG was investigated as an adjuvant therapy to surgery and chemo-radiotherapy in the 1970s. Early studies demonstrated that it was feasible and safe to deliver BCG intra-pleurally.^24 26 64^ Furthermore, intra-pleural BCG was associated with a reduction in recurrence rate and an overall survival benefit compared with placebo.^24 25 64-68^ Results were conflicting however, with a similar number of trials that demonstrated no difference in survival or recurrence following intra-pleural BCG.^27 28 69-72^ A small number of studies suggested intra-pleural BCG was associated with worse outcomes,^26 73^ although in one of these BCG (without chemotherapy) was compared to standard chemotherapy – a design that would be deemed unethical in the current day, as it entailed withholding a known-to-be-efficacious treatment from a proportion of participants.^73^ Additionally several of the negative studies were non-randomised^27 28^, and many consisted of small participant numbers, with consequent high risk of being underpowered to detect differences in survival.^26 70 71^

Another explanation for the different outcomes observed in BCG trials may lie in the different BCG strains used. It has been established that TICE strain is more effective than Connaught, and of the lyophilised vaccines, Pasteur is more effective than Glaxo.^74 75^ It may be relevant, therefore, that three of the six trials that demonstrated a positive effect of BCG used the TICE strain,^24 25^and it is for this reason that TICE has been chosen for TILT.

Whether or not intra-pleural BCG is effective in lung cancer, there remains a strong case for potential efficacy in mesothelioma. Mesothelioma is similar to melanoma, both in terms of histological appearance and some of the genetic mutations underlying the tumours.^76 77^ Their mutual sensitivity to immune stimulation is further cause to suspect mesothelioma may respond to local BCG treatment as melanoma has done. In addition, as a localised tumour affecting a discreet body cavity, mesothelioma resembles early-stage bladder cancer and offers a similar opportunity for targeted delivery of anticancer drugs directly into the tumour environment. Animal studies have shown that proximity of BCG to tumour was an important factor in producing an effective response.^78^ Unlike lung cancer, where intra-pleural administration was anatomically distant from the original site of disease, intra-pleural administration in mesothelioma delivers the drug into direct proximity with the tumour, thus creating immune stimulation in the very area it will be most effective.

## The trial within a cohort (TwiC) methodology

To investigate intra-pleural bacterial immunotherapy in MPM, this research will use the trial within a cohort (TwiC) design.^41^ This is an innovative, highly pragmatic methodology that aims to replicate real-life clinical practice. The methodology uses an existing observational cohort to identify eligible participants for a trial. Eligible participants are randomly selected to receive the trial intervention, and subsequently recruited to the trial. Non-selected participants remain in the cohort as controls and are not informed about the intervention. This methodology has received ethical approval in trials in patients with breast cancer, rectal cancer and metastatic bone disease (<https://clinicaltrials.gov/show/NCT02070146>).^79^

The TwiC methodology offers certain benefits over traditional randomised controlled trial (RCT) design.^41^ The presence of a cohort allows simultaneous collection of observational data that can provide information on the natural history of the disease. Cohort studies are associated with faster recruitment and more diverse participant characteristics, which is a benefit in MPM research, where recruitment can be slow and participant diversity narrow.^16 80^

TwiCs also offer the following advantages for MPM research:

- Chemotherapy has limited efficacy in MPM, and most patients don’t receive it.^2^ Consequently, many people participate in trials in the hope of gaining access to a treatment that is not otherwise available. However, if a trial includes a standard care arm, participants may decline randomisation or withdraw from the trial if allocated to this arm. Alternatively, they may feel disappointed, which could affect patient-reported outcomes. The TwiC design removes this issue, and may therefore reduce attrition bias and reporting bias.^41^
- In an RCT, participants are given information about all trial interventions and then randomly allocated to one. This differs from real-life clinical practice where patients are provided with information about treatment as and when they are going to receive it, and not if they are not going to receive it. TwiCs replicate this by providing information about the intervention solely to participants selected to receive it. This has 2 benefits; firstly it removes the ethical issue of informing a participant about an intervention that they only have a 50% chance of receiving, and secondly it increases the generalisability of the trial results by replicating real-life clinical care. It also creates a patient-centred consent process, whereby each participant provides consent for the exact treatment they will receive and none that they will not. This aligns well with the culture of open communication that exists between clinicians and patients with MPM.
- The TwiC methodology respects patients’ choices to decline treatment. These participants would be excluded from an RCT, but can participate in a TwiC and contribute observational data to the cohort.
- Using the TwiC design, participants in a cohort can be sampled repeatedly, allowing multiple trials to be undertaken, with shorter recruitment times. If this research demonstrates feasibility, further MPM trials can be undertaken from the same cohort. This could reduce recruitment times, reduce research costs and improve overall efficiency.

# AIMS & OBJECTIVES

The aim of this study is to answer the question “Is it feasible to undertake a TwiC of intra-pleural bacterial immunotherapy in MPM and is it acceptable to patients and relatives?”

The outputs from this research will inform the design of a subsequent full-scale trial. The long-term goal is to determine whether bacterial immunotherapy, in the form of either OK432 or BCG, is an effective treatment for MPM, and whether the TwiC design is appropriate for MPM trials.

# 5. MESOTHELIOMA COHORT STUDY (ASSESS-meso)

## Setting

The TILT trial will be embedded within an existing MPM cohort (ASSESS-meso, IRAS project ID 220360). ASSESS-meso is a multi-centre, prospective, pragmatic cohort study with recruiting centres across England.

ASSESS-meso aims to mimic routine clinical follow up, with a flexible schedule of assessments ranging from weekly to three monthly. Assessments occur at every clinical visit, and include clinical review, patient-reported symptom scores (on visual analogue scales, VAS), quality of life questionnaires (EQ-5D-5L), blood tests including serum mesothelin, pleural fluid drainage and analysis and imaging including CXR and TUS. CT scans will be done 4 monthly.

Participants will continue follow up within ASSESS-meso until death, withdrawal from the study or loss to follow up.

## Inclusion criteria (ASSESS-meso)

Participants who have enrolled in ASSESS-meso have met all of the following criteria:

- Histological, cytological or clinico-radiological diagnosis of MPM, confirmed at mesothelioma multidisciplinary meeting (MDT)
- Willing and able to comply with study follow up assessments

## Exclusion criteria (ASSESS_meso)

Participants in ASSESS-meso have none of the following criteria:

- Age <18 years old
- Unable to give written informed consent

## TwiC specific consent within ASSESS-meso

Participants who have enrolled in ASSESS-meso will have provided written informed consent for all study-related assessments at the time of enrolment. In addition the possibility of being identified as eligible for future trial will have been explained to them. They will have been asked to sign a specific consent statement that states “I am willing for my information to be used to identify other research trials that I am eligible for. I am willing to be chosen, on a random basis, to be invited to discuss these trials. I am aware that the decision to participate in future trials will be made at the time of discussion, and does not need to be made now. My decision to participate in future research trials will not affect my medical care, legal rights or ability to participate in the current study.”

Therefore, participants who have consented to be randomly selected for future TwiCs will be potentially eligible for TILT (providing they meet the TILT eligibility criteria outlined in sections 6.2 and 6.3). Participants who have enrolled in ASSESS-meso, but declined to be considered for future TwiCs will not be eligible for TILT.

## TILT and ASSESS-meso related activities

Screening for TILT eligibility and subsequent randomisation of eligible participants are designated ASSESS-meso activities. They are included in the TILT protocol and flow diagram for clarity.

Participants who are selected to participate in TILT will be asked to provide further written informed consent for TILT. If they consent to participate, the first TILT-related activity will be the intervention visit. Participants who decline to join TILT and participants who are not randomly selected will continue to undergo follow up assessments ASSESS-meso. Follow up of these participants will be organised to match the TILT follow up schedule, in accordance with the flexible follow-up schedule stated in the ASSESS-meso protocol.

Participants will only be considered to be participating in the TILT trial if and when they have signed the TILT consent form. All activities that are undertaken prior to TILT consent are considered ASSESS-meso related activities and are included in the ASSESS-meso protocol.

For ease of statistical analysis, TILT follow up assessments will be timed from the point of randomisation, i.e. Week 0 will be the Screening Visit even though this is not an official TILT-related assessment. This is preferable to using TILT consent date as Week 0, as there is no equivalent visit for the ‘control’ participants in ASSESS-meso (as they are not asked to give further consent). No TILT-related activities will take place between randomisation and the participant providing consent for TILT. The time between the two visits is expected to be a few days, to allow the participant sufficient time to consider the TILT Participant Information Sheet, and decide whether they wish to enrol in the trial.

# TRIAL METHODOLOGY

## Trial setting

The trial will be conducted at 3 hospitals with specialised pleural disease services ; North Bristol NHS Trust (NBT), Oxford Centre for Respiratory Medicine (OCRM) and Musgrove Park Hospital, Taunton.

## Inclusion criteria

To participate in TILT patients must meet all of the following inclusion criteria:

- Histological or cytological diagnosis of MPM
- Enrolled in ASSESS-meso and has given consent to be considered for and be randomly selected for future trials
- Functioning IPC in situ or IPC that has ceased to drain, but can be successfully flushed with 20mls of normal saline.

OR willing to have an IPC and has a pleural effusion suitable for IPC insertion

- No chemotherapy in preceding 4 weeks and none planned in subsequent 4 weeks
- Performance status ≤2, or PS 3 and felt clinically suitable for trial
- Predicted survival ≥12 weeks from enrolment
- Able to give written informed consent & meet trial requirements

## Exclusion criteria

To be eligible for TILT, participants must have none of the following exclusion criteria:

- No IPC in situ, and has contra-indication to IPC insertion
- Trapped lung with <50% pleural apposition on x-ray
- Moderately heavy or heavily loculated pleural effusion
- Known immunodeficiency or immuno-suppressive medication
- Intercurrent infection (pleural or elsewhere) or clinical signs of sepsis (treatment should be deferred for 7-14 days)
- Active tuberculosis
- Known sensitivity or allergy to OK432, BCG or penicillin
- Previous treatment with immunotherapy
- Recent (<2 weeks) thoracic surgery or invasive pleural intervention (excluding diagnostic/ therapeutic pleural aspiration)
- Currently enrolled in any other interventional clinical trial
- Brain metastases or CNS involvement of MPM
- Pregnancy or lactation, current or planned during the study period
- Age <18
- Any other factor that, in the opinion of the Chief Investigator, would mean participation in the study would be contraindicated

## Random selection

Participants who meet the eligibility criteria will be identified at scheduled assessment visits during their follow up in ASSESS-meso (TILT screening visit). By capturing data on all potentially eligible participants, the ASSESS-meso database will also function as a screening log, recording data on patients who decline to join TILT, and documenting their reasons for non-participation.

12 eligible participants will be identified and randomly allocated to receive either OK432 or BCG or to remain in ASSESS-meso as a control. Random selection will occur at Week 0. Randomisation will be undertaken by a centralised, concealed, computerised randomisation service, stratified by WHO/ECOG performance status on the day of randomisation, following drainage of (0 vs ≥1) and tumour sub-type (epithelioid/cytological diagnosis versus non-epithelioid). Screening and randomisation are designated ASSESS-meso related activities.

Participants will be unaware that random selection is taking place. Participants who are selected to receive either OK432 or BCG will be informed, provided with a participant information sheet and given time to consider participation, before being invited to join the trial. Participants who are not selected will continue observational follow up in ASSESS-meso. This process is acceptable to ethics committees, as to be eligible for TILT, participants in ASSESS-meso must have already given their consent to undergo randomisation for future trials.

## Consent

Participants who are randomly selected to receive either OK432 or BCG will be informed that they have been chosen to participate in a trial of a potential new treatment, and if they agree to participate they will definitely receive their allocated treatment. They will be provided with an information sheet about the trial, which will contain information about OK432 and BCG including potential risks and side effects. They will be allowed sufficient time, as determined by the patient, to consider trial entry, and will be offered the opportunity to discuss the trial with a member of the research team, and ask questions.

Once the participant is satisfied with the information they have been given they will be asked to provide written, informed consent to participate in TILT. A copy of the consent form will be sent to the coordinating trial centre (North Bristol NHS Trust) for documentation.

Patients who choose not to participate will be asked to provide brief reasons for non-participation, and will then return to follow up within ASSESS-meso. Their schedule of visits will be organised to match the TILT assessment schedule, in accordance with the flexible follow up stated in the ASSESS-meso protocol. These patients’ data will be collected as part of the feasibility outcomes for TILT.

Participants who are eligible for TILT but are allocated to the ‘control’ arm will continue follow up in ASSESS-meso, with a schedule of visits that matches the TILT assessment schedule. This is in accordance with the ASSESS-meso protocol, which includes the option for flexible follow-up visits, from weekly to 3 monthly. They will form a control group who will provide comparison data for the OK432 and BCG arms. If possible, **they will be blinded to the existence of the OK432 and BCG arms, and the TILT trial***.* This design replicates real-life clinical care where patients are informed about treatments as and when they are going to receive them, and not if they are not going to receive them.

Having provided full, informed, written consent for ASSESS-meso, participants in the control arm will not be asked to provide additional consent for TILT. In this way, every participant within ASSESS-meso and TILT will provide specific consent solely for the procedures they will undergo. Given that many patients join trials in the hope of receiving a treatment that is not otherwise available, this individualised consent process prevents disappointment in patients allocated to the ‘standard care’ or control arm.

Participants will only be considered to be participating in the TILT trial if and when they have signed the TILT consent form. All activities that are undertaken prior to TILT consent are considered ASSESS-meso related activities and are included in the ASSESS-meso protocol.

## Sample size & recruitment targets

The target sample size is 12 participants, of whom 8 will be randomly selected to be offered OK432 or BCG and 4 will continue in ASSESS-meso as controls. This sample size is based on previous feasibility studies in mesothelioma.

It is anticipated that the majority of participants who are offered bacterial immunotherapy will consent to receive it, as the burden of treatment is low (a single dose on one occasion), the treatment intervention is well established in other clinical scenarios and trial follow up assessments are the same as they would be within ASSESS-meso. Non-acceptance of bacterial immunotherapy will be collected as part of the feasibility outcomes for the trial, and qualitative interviews will explore these participants’ reasons for non-acceptance of bacterial immunotherapy.

## Co-enrolment guidelines

For the duration of a patient’s involvement in TILT, they will not be permitted to enter into any other clinical trial of an investigational medicinal product or interventional clinical trial. Should a participant be considered for co-enrolment in another trial then liaison with the TILT research team is essential. This includes participants in the control arm and the bacterial immunotherapy arms. Previous participation in an interventional trial is not an exclusion criteria, as long as any interventional treatment was received at least 4 weeks prior to enrolment in TILT.

Once a participant has completed the 12 week data collection period for TILT, they will remain in ASSESS-meso, and continue observational follow up. If they become eligible for further trials they will be free to participate in them. However, patients may only be randomised into TILT once.

If TILT demonstrates feasibility of the TwiC design, further trials will be designed using the same methodology and embedded within the same MPM cohort. This offers the potential to reduce recruitment times for trial, by drawing on an existing “research-ready” population and allows participants in ASSESS-meso the opportunity to be considered for multiple trials during their participation in the study.

# TRIAL TREATMENTS

## Arm 1 -OK432

OK432 is a heat-treated, penicillin-killed lyophilised streptococcal preparation derived from the SU strain of Streptococcus pyogenes group A2 (Picibanil, Chugai Pharmaceutical Ltd, Tokyo, Japan). It is composed of dried streptococcal cells containing penicillin G potassium at a dose of 26,900 units/mg of dried cocci. A dose of 1 (KE) is equivalent to 0.1 mg of dried streptococci. OK432 is supplied as a dry white powder in vials of 1KE. For intra-pleural delivery, it will be reconstituted in 50ml of sterile 0.9% saline prior to instillation into the pleural cavity.

OK432 is an unlicensed product in the UK. It is licensed in Japan, South Korea and Taiwan for the topical treatment of lymphangiomas and head and neck cancers, and for intra-cavitatory treatment of malignant pleural effusions and ascites.^81-84^ It has been used in routine clinical care as a pleurodesis agent for malignant pleural effusions and refractory pneumothoraces since 1975 in these countries.

Intra-pleural OK-423 is associated with a few specific side effects, including a mild, transient fever and pain following injection. These adverse events are commonly seen with many pleurodesis agents, and are likely to represent pleural inflammation. Other side effects seen with OK432 include malaise and anorexia, but these have always been reported as mild and self-limiting. There have been no reported deaths related to OK432 treatment.

The dose of OK432 used in TILT is 10KE. However, it is permissible to use a reduced dose of 5KE in patients who are a high risk of adverse events, or who would find it difficult to manage an adverse event. For example, elderly patients, patients with performance status of 2-3, patients with multiple medical co-morbidities, especially cardiac or renal, and patients who live alone or have high care needs at home. The decision to use the reduced dose should be made by the treating clinician prior to IMP administration, and can be discussed with the Chief Investigator if desired.

## Arm 2 – BCG

BCG is a live attenuated, low virulence strain of mycobacterium Bovis prepared from a culture of Bacillus Calmette-Guérin (OncoTice, Merck Sharp & Dohme Ltd, The Netherlands). It comprises a freeze-dried preparation of bacilli, with each 12.5mg vial containing 2-8 x 10^8^ colony forming units (CFU). It is supplied as a dry powder, which will be reconstituted in 50ml of sterile 0.9% saline prior to instillation into the pleural cavity. 1ml of the reconstituted solution, containing 0.4-1.6 x 10^7^ CFU, will be delivered intra-pleurally. A reduced dose of 0.5ml (0.2-0.8 x 10^7^ CFU) can be used in patients who are a high risk of adverse events, or who would find it difficult to manage an adverse event. For example, elderly patients , patients with performance status of 2-3, patients with multiple medical co-morbidities, especially cardiac or renal, and patients who live alone or have high care needs at home. The decision to use the reduced dose should be made by the treating clinician, and can be discussed with the Chief Investigator, or responsible delegate (Dr Anna Bibby) if desired.

BCG does not have marketing authorisation for intra-pleural use in the UK. However it has been used as an intra-cavitory treatment for bladder cancer since the 1970s. Common adverse events following intra-vesical delivery include local inflammatory effects e.g. cystitis, dysuria, haematuria and systemic inflammatory symptoms including fever, malaise, fatigue and flu-like symptoms. It is anticipated that similar effects will be seen with intra-pleural delivery, with pleuritis, pain and bloody effusions as manifestations of local inflammation. Other common effects from intra-vesical delivery include local infection, nausea and vomiting, and arthralgia & myalgias. Uncommon adverse events include disseminated BCG infection, granulomatous hepatitis and rashes.

BCG has previously been used intra-pleurally in trials of patients following thoracic surgery for lung cancer. Transient fevers, rigors and malaise were common, as with intra-vesical delivery. Additional common adverse events were empyema, infected surgical wounds causing fistulation and late BCG pleural infection (>6 months after delivery).^70 72^ This is likely to reflect the recent major thoracic surgical intervention undergone by participants in these studies. To minimise the risk of infection, either BCG-related or bacterial, recent thoracic surgery or pleural intervention is an exclusion criteria for TILT.

Some studies gave participants prophylactic isoniazid following intra-pleural BCG, however there was no difference in adverse event rates between the trials that did and did not use prophylaxis, and it was concluded that isoniazid had no effect in reducing adverse events.^70^ For this reason, and because isoniazid toxicity is increased in people over the age of 50, prophylactic isoniazid will not be given in TILT.

## Intervention visit

Participants who are randomised to bacterial immunotherapy will be invited to receive a single administration of OK432 or BCG, depending on which arm they are allocated to. The dose of OK432 is 10KE and the dose of BCG is 0.4-1.6x10^7^ CFU.. However, reduced dose options (5KE of OK432 and 0.2-0.8 x 10^7^ CFU of BCG) can be used (based on the discretion of the treating clinician) in older patients, those with higher performance status, people with multiple medical co-morbidities, or those at higher risk of experiencing or not coping with an adverse event. The immunotherapy agent will be delivered intra-pleurally via an indwelling pleural catheter within 14 days of random selection (i.e. days 0-14).

For the intervention visit, participants will attend hospital and undergo medical assessment to ensure they remain eligible and suitable to receive OK432/BCG. This will include complete drainage of the effusion, to ensure IPC patency, followed by a CXR to exclude trapped lung. If they remain eligible, up to 3mg/kg of 1% lignocaine (to a maximum of 250mg) will be instilled intra-pleurally followed by the appropriate agent, in adherence with the Trial Specific Protocol 4 (TSP 04). The IPC will then be disconnected and OK432/BCG left within the pleural cavity for 1 hour. After 1 hour, further drainage will be undertaken and the participant will be observed for another hour before returning home.

Following the intervention, patients will be advised to take regular prophylactic paracetamol and ibuprofen for the subsequent 3 days to reduce the risk of fevers, sweats and pain.

## Storage, dispensing and packaging of OK432

OK432 will be imported from Japan by Pharmaceutical Solutions Ltd, a UK-based pharmaceutical import agency that accesses unlicensed medications for clinical and research use. Batch assurance, and QP release will be undertaken prior to use in the trial. Pharmaceutical Solutions Ltd will provide full batch traceability in accordance with Good Manufacturing Practice (GMP) and Good Distribution Practice (GDP) guidelines. Batch labelling of IMP is not required.

OK432 will be delivered directly to pharmacies at the collaborating centres. OK432 requires refrigerated storage at a temperature between 2°C and 8°C. In this environment it has a shelf-life of 12 months.

Each course of trial treatment will be pre-prepared in a trial pack (consisting of the appropriate dose of IMP and 50ml of normal saline to enable administration) and dispensed by pharmacy staff to the clinic on the date of intervention visit. Labelling of trial packs will be undertaken at site pharmacies by a clinical trial pharmacist or appropriate delegate, in accordance with TSP05 ‘pharmacy dispensing’. OK432 will be dispensed in sealed boxes (1 box for 5KE dose, 2 boxes for 10KE dose) as produced by the manufacturer, and certified by the QP. Labelling of individual vials will not be undertaken as to do so would obscure the original labelling, and prevent safe reconstitution.

OK432 will be reconstituted in clinic by the trial investigator. The product will be dispensed open-label in its original packaging, with an additional label stating it is an investigational medicinal product (IMP). Pharmacy storage and dispensing of the IMP will adhere to Trial Specific Procedure 5 (TSP05).

## Storage, dispensing and packaging of BCG

BCG will be procured through the usual pharmacy procurement pathways at each centre. BCG requires refrigerated storage at a temperature between 2°C and 8°C, protected from light. In this environment it has a shelf-life of 12 months.

Each course of trial treatment will be pre-prepared in a trial numbered pack and dispensed by pharmacy staff to the clinic on the date of intervention visit. BCG will be dispensed as produced by the manufacturer to be reconstituted in clinic by the trial investigator. The product will be dispensed open-label in its original packaging, with an additional label stating it is an IMP. Pharmacy storage and dispensing of the IMP will adhere to Trial Specific Procedure 5 (TSP05).

## Accountability & unused drugs

Compliance will be audited by completion of electronic case report forms (eCRF) for every administration of OK432/BCG. All used, partially used or unused treatments will be destroyed at study site. The IMP inventory form will be used by the pharmacists to document destroyed IMP.

# NON-TRIAL TREATMENTS

## Usual care

All patients should have been discussed, and had their diagnosis ratified at MDT. Patients should have been referred to their local oncologist for discussion and consideration of chemotherapy and other oncological treatments in the usual manner. Treatment decisions regarding the ongoing clinical care of all participants remain with the primary physician or treating team.

Routine clinical review will take place in the respiratory clinic. The frequency of clinical review will depend on patient choice, severity of symptoms and clinical discretion. Participants who require clinical review more frequently than specified in the TILT schedule of visits will have an eCRF completed for every additional visit.

All attempts should be made to co-ordinate trial follow-up and routine follow-up appointments. Patients should be given the contact details for a mesothelioma specialist nurse at the earliest opportunity.

## Indwelling pleural catheters

### 8.2.1 IPC insertion

An IPC can be inserted by any appropriately trained clinician, including respiratory physicians, thoracic surgeons or member of the research team. The decision to insert an IPC is down to the discretion of the treating clinician.

To be eligible for TILT, participants who do not have an IPC in situ must be willing to have one inserted and must have a pleural effusion of sufficient size and no contra-indications to insertion. These participants should have an IPC inserted as soon after baseline assessment as possible. Participants with a recently inserted IPC should wait at least 14 days before receiving OK432/BCG, if applicable.

### 8.2.2 IPC drainage

IPC drainage will take place in the community, either by district nurses or a trained family member or next of kin. If there are no community resources available to perform drainage, the participant may be drained at their local trial centre.

The drainage regimen will be dictated by clinical need and will be decided by the treating clinician in conjunction with the patient and community team. It is expected that most patients will require drainage approximately 2-3 times per week. Drainage diaries will be kept by all participants.

Participants in the bacterial immunotherapy arms will undergo complete drainage prior to IMP delivery to ensure the drain is patent, and to exclude underlying trapped lung.

### 8.2.3 IPC blockage

All care should be taken to ensure IPCs do not become blocked, beginning with an adequate flush at the end of sclerosant administration. If there is a suspicion that blockage has occurred, perhaps due to cessation of drainage with persistent changes on CXR or TUS, then standard local unblocking procedures should be followed. This may involve a short hospital admission for administration of intrapleural urokinase. Such events should be documented on the appropriate eCRF, on an adverse event form (or SAE form if appropriate) and, as per normal, in the patient’s notes. TSP02 provides a suggested protocol for drain unblocking.

### 8.2.4 Assessment of pleurodesis

Successful pleurodesis is defined as reduction in fluid drainage to ≤50ml on 3 consecutive drainage attempts, with opacification of ≤25% of the ipsilateral hemithorax on CXR. Where there is uncertainty about the presence of fluid on CXR, TUS should be undertaken to assess the volume of residual fluid. If successful pleurodesis is confirmed, the IPC should be removed as soon as possible.

### 8.2.5 IPC removal

Once inserted, drains may be removed at any time at the discretion of the patient’s primary physician, at the request of the patient, or at the discretion of the trial team. Potential reasons for removal include subcutaneous or pleural infection, intolerable pain, significant fluid loculation, or successful pleurodesis. Reason for removal will be documented on eCRFs.

If a drain is to be removed, patients should be given an appointment to have this done within 14 days of the clinical assessment at which this decision was taken. Removal of indwelling pleural catheters should be performed by trained staff under aseptic conditions, and should be followed by a chest x-ray.

Any participant who has their drain removed during their trial period will continue to undergo planned follow-up for the full trial period. Drain removal will be documented on eCRFs.

## Permitted medications

Participants are expected to continue the use of prescribed medications during the course of the study, as identified at baseline assessment, and consistent with study inclusion and exclusion criteria. In addition, all treatments that are considered necessary for a participant’s welfare may be administered at the discretion of the treating clinician or trial investigator in keeping with standard medical practice.

Participants in both arms will receive best supportive care alongside the investigational products, with the exception of oral steroids, which should be avoided, if possible, in the 14 days before and after OK432/BCG delivery. Analgesia, anti-emetics and antipyretics can be given as required, in accordance with local prescribing guidelines.

The following medications will be delivered to all participants who receive OK-423 or BCG, as stated in the trial manual:

- Up to 200mg 1% Lignocaine administered intra-pleurally contemporaneously with OK432/BCG delivery.
- Paracetamol, oral or intra-venous to a maximum dose of 4g per 24 hours, delivered immediately prior to OK432/BCG delivery and as required subsequently
- Opioid analgesia, including oral codeine phosphate 30-60mg to a maximum of 240mg in 24 hours, or oral morphine sulphate at a dose that is appropriate to the participant’s current and previous use of morphine, delivered immediately prior to OK432/BCG delivery and as required subsequently
- Medications to treat any treatment-emergent AEs

Medications to treat treatment-emergent AEs should not interfere with the study and can be used at the investigator’s discretion. Antipyretics and anti-inflammatories may be used to treat fever or to prevent recurrence of fever.

Subjects with clinical symptoms or signs of infection should have a clinically relevant evaluation, including appropriate bacterial cultures. If pleural infection is suspected, a sample of pleural fluid should be taken and sent for biochemical and microbiogical assessment. If antibiotics are required, in the opinion of the treating clinician, local guidance should be followed, to include gram positive and anaerobic cover. The preferred regimen would include either co-amoxiclav or Tazocin. The decision regarding intra-venous or oral treatment should be made by the treating clinician.

Participants in the control arm are permitted to receive chemotherapy or immunotherapy at the discretion of the treating clinician. Wherever possible, however, the commencement of disease-modifying treatments should be delayed until the end of the 12 week TILT trial period.

## Non-permitted medications

The following therapies are not permitted during TILT:

- Any chemotherapy or immunotherapy (participants who receive the intervention only)
- Granulocyte colony stimulating factor (GCSF) or Granulocyte macrophage colony-stimulating factor
- Other known immunosuppressive or immunomodulatory medication

## Permitted interventions

Interventions to control pleural fluid and procedures to control pain (e.g. cordotomy) can be employed as necessary, but ideally should be avoided within 14 days of OK432/BCG delivery. Thoracoscopy to the ipsilateral pleural space should be avoided in the 14 days after OK432/BCG delivery. Insertion of in-dwelling medical devices, e.g. Hickman or PICC lines, portacaths, IPCs, should be avoided within 14 days of OK432/BCG delivery but can be undertaken outside this period. Participants who have a medical device in situ at the start of the trial can participate in the trial without limitation.

## Non-permitted interventions

Thoracic surgery to the ipsilateral pleural space or chest wall should be avoided unless absolutely necessary for life-saving reasons. Where thoracic surgery is undertaken, prophylactic antibiotics should be considered to prevent post-operative complications.

## Data on concomitant medications and interventions

All concomitant medication taken during the trial will be recorded on eCRFs including all prescription, over-the-counter (OTC), herbal supplements, and IV medications and fluids. Documentation of concomitant medication should include generic name, dose, frequency, route, date of any changes occurring during the trial period, and indication. All interventions undertaken during the trial period will be recorded on eCRFs.

# TRIAL FOLLOW UP ASSESSMENTS

## Trial visits

As well as an initial screening visit at Week 0, participants will attend 3 trial assessment visits within the subsequent 12 weeks, with participants in the intervention arm returning for two additional visits; one to receive OK432/BCG within 14 days of randomisation (i.e. days 0-14) and a follow-up safety visit on day 3 post-IMP administration. Trial visits will take place at 3 weeks, 6 weeks and 12 weeks post-randomisation.

Participants who receive an IMP will also be contacted by telephone by a member of the trial team, daily for up to 5 days after IMP administration, to check on their well-being. Trial visits must occur as scheduled +/- 3 days, with the exception of the 3 day post intervention visit which must occur on day 3. Trial procedures and assessments for each visit are detailed in section 9.7. The trial flowchart is shown in section 2.4.

Having completed the 12 week trial period, participants will return to clinical follow up as part of the ASSESS-meso. They will participate in ongoing data collection in accordance with the ASSESS-meso protocol which will continue until death, loss to follow up or withdrawal from ASSESS-meso.

## Feasibility assessments

The primary outcome for the trial is feasibility. This will be based the following feasibility targets:

- Recruitment rates to time & target >66% at end of the trial
- Attrition rate of <20% (nb this relates solely to attrition due to loss to follow up or participant withdrawal, it does not include attrition due to participant death)
- Data completeness rates >90%

In addition, data will be collected on specific elements of the TwiC methodology to explore the acceptability of the trial design to participants. This will include determining how many participants in the control arm were aware that the intervention arm existed, and that they had been randomised to be controls. Information will also be collected on the number of participants who were randomly selected to receive either OK432 or BCG, but declined to do so. These participants will continue to be followed up in accordance with the TILT protocol. In order to determine whether participants who decline bacterial immunotherapy differ in any way to other study participants, their baseline characteristics and clinical outcomes will be compared with the control arm and with participants who received immunotherapy, as described in Section 13.

Data from ASSESS-meso will also be used to explore the characteristics and outcomes of patients who consented to join ASSESS-meso but declined to be considered for future trials.

## Efficacy assessments

Exploratory efficacy data will be collected at trial assessment visits. This will include blood tests, including the biomarker mesothelin, tumour response rates based on CT appearances and assessed using the modified RECIST criteria,^42^ and effect on pleural fluid, based on pleural fluid drainage diaries and pleurodesis rates. Patient-reported outcome measures (PROMS) of breathlessness and chest pain, as well as quality of life questionnaires will be completed by the patients at each visit and daily for a period between Week 0 and Week 3. If logistically possible, participants in the BCG arm will undergo Mantoux testing before and after BCG delivery to assess for changes in immunological response. Samples of pleural fluid will be collected at each visit and stored for future testing for cytokine levels and mesothelin. Additional pleural fluid samples will be stored in a pleural fluid biobank for immunophenotyping of T cell populations and further cytokine analysis at a later date. Specific consent to store biological samples for future testing will be obtained at enrolment.

## Safety assessments

Details of adverse event and serious adverse event reporting are presented in section 12. Adverse events will be assessed on each assessment visit and reported on eCRFs and specific adverse event forms. There is a ‘free text’ section on the adverse event form to allow staff to document any specific adverse event features as reported by patients. As well as usual adverse event reporting, eCRFs will specifically record deaths within the study period and episodes of systemic sepsis or pleural infection.

## Source data

Electronic case report forms (eCRFs) will be used to collect data at each study visit. Paper check-lists will be provided to each study centre to use as a prompt for eCRFs if desired. Completed checklists are not classified trial documents and can be filed in the patients’ notes to act as source data or stored or destroyed at the respective study centre.

The primary data source will be the participant’s medical notes. The laboratory reports will form the primary data source for blood results. The CT scan report will form the primary data source for any scans. Assessment of response will be undertaken by an independent, blinded radiologist, and confirmed and the regional MPM MDT.

The screening logs, consent forms and eCRFs will be the source data for the research-specific data. Patient-reported outcome measures, such as symptoms, quality of life and pleural fluid drainage, will use the individual participants’ diaries as source data.

## End of trial (EoT)

Trial recruitment will cease in November 2019, regardless of the number of participants enrolled. EoT date, therefore, will be 12 weeks after enrolment of the 12^th^ trial patient or 12 weeks after the recruitment window closes, whichever happens first. At EoT, participants will return to clinical follow up within ASSESS-meso, which will continue until death, loss to follow up or withdrawal from the study. Data from ASSESS-meso will be used to evaluate overall survival at EoT.

|  |  | **Screening**  **Week 0*** | **Intervention**  **Day 0-14** | **3 days post-intervention**  **(Week 3)** | **Week 6** | **Week 12** |
| --- | --- | --- | --- | --- | --- | --- |
| **Clinical assessment** | Assessment of eligibility | X | **X** |  |  |  |
|  | History of pleural interventions | X |  | X | X | X |
|  | Previous oncological treatment | X |  |  |  |  |
|  | Medication history | X | **X** | X | X | X |
|  | WHO/ECOG performance status | X | **X** | X |  |  |
|  | Vital signs, pulse oximetry | X | **X** |  |  |  |
|  | Physical examination | X |  |  |  |  |
|  | Insertion of IPC (if not already in situ) | X |  |  |  |  |
|  | Pleural fluid drainage diary review | X |  | X | X | X |
|  | Documentation of AEs |  | **X** |  | X | X |
| **Blood tests** | Screening blood tests (HIV screen, hepatitis screen, immunoglobulins, random glucose, thyroid function tests) | X |  |  |  |  |
|  | Pregnancy test  (if female <55 yrs) | X |  |  |  |  |
|  | Coagulation panel | X |  |  |  |  |
|  | Trial blood tests (Full blood count, urea & electrolytes, liver function tests, C reactive protein) | X | **X** | X | X | X |
|  | Serum mesothelin | X |  | X | X | X |
|  | Pleural fluid sampling and storage | X | **X** | X | X | X |
|  | Mantoux test (if logistically possible, BCG arm only) | X |  |  |  | X |
| **Randomisation & intervention** | Random selection | X |  |  |  |  |
|  | Informed consent for TILT |  | **X** |  |  |  |
|  | Administration of OK432/BCG |  | **X** |  |  |  |
| **PROMS** | Breathlessness & chest pain VAS score | X |  | X | X | X |
|  | Daily VAS score completion |  |  |  |  |  |
|  | EQ-5D-5L QoL questionnaire | X |  | X | X | X |
| **Radiological imaging** | Chest x-ray | X | **X** | X | X | X |
|  | Thoracic ultrasound | X | **X** | X | X | X |
|  | CT chest (within preceding 2 weeks) | X |  |  |  | X |
| **Qualitative research** | Participant interviews |  |  |  |  | X |
|  | Focus groups with relatives/carers |  |  |  |  | X |

## Schedule of visits

* Screening visit is an ASSESS-meso –related activity, however it is included in the schedule of visits for clarity of the trial process

# QUALITATIVE RESEARCH

## Existing qualitative literature in MPM

MPM carries a significant physical, psychological and social burden, with specific issues arising as a result of the occupational nature of the disease.^85^ Physically many patients experience a rapid deterioration in physical health, which can be difficult for people who previously relied on their physical strength.^85^ Psychologically, some patients will have lived with anticipatory anxiety for years, and the diagnosis of mesothelioma is met with resignation and “stoical fatalism”.^86 87^ Depression is common, as is anger^88 89^. Patients may struggle with the burden of extensive medical interventions.^88 90^ Additionally the process of applying for compensation can be stressful, emotionally draining, and uncomfortable, particularly for those who consider themselves “self-reliant”.^88^

Although the experience of mesothelioma is well researched, it is not yet clear how patients view taking part in mesothelioma research. Considering the challenges of living with this condition, it would be understandable if people with mesothelioma were reluctant to participate in trials. Numerous studies acknowledge the difficulty of recruiting people with incurable or palliative conditions.^91-93^ However, evidence also suggests that many of these patients are interested in research, and may benefit from participation.^93^ Additionally, qualitative methods can be useful in clinical trial settings to help identify potential recruitment barriers and enhance participation.^94 95^

## Aim of qualitative research

The aim of the qualitative research is to explore the experiences of participants and their relatives/carers of taking part in TILT and to assess the acceptability of research processes to them. The qualitative research aims to answer the question “Was TILT acceptable to participants and their relatives?”

## Methodology for in-depth interviews

Following completion of the trial, a sample of participants will be invited to undertake an individual face-to-face interview with Dr Anna Bibby. The sample will be purposively sampled to include participants from both arms of the study and participants who declined any element of the research, including those who declined to receive bacterial immunotherapy and those who requested not to be considered for future trials when they initially joined ASSESS-meso.

Interviews will be held after the final trial visit in order to prevent the interviews having any influence on trial outcomes.

At Week 6, participants will be provided with a patient information sheet describing the qualitative interviews. They will be given sufficient time to read it, and have the opportunity to discuss the research with a member of the trial team. To minimise inconvenience to participants, interviews will be conducted on hospital premises in a quiet and private room, however participants will be offered the option of being interviewed at home if they would prefer. Participants will be able to choose whether to have the interview on the same day as their final trial visit, or whether to schedule it for another day at their convenience. Interviews must be conducted within 14 days of the final trial visit.

Immediately before the interview, participants will discuss the qualitative study with the researcher, before being asked to provide written informed consent to participate. This will include specific consent to be audio-recorded, for the audio-recording to be stored electronically and for anonymised quotes to be used in the final report. Should a participant decline audio-recording this will not preclude their participation in interview, and the interviewer will seek their agreement to take notes instead.

Ideally, interviews will be held with just the participant and the interviewer present. However, the study also seeks to invite relatives and carers to take part, and this work will take place as focus group research as described in section 10.4. It is anticipated that patients will have different experiences and perspectives to their relatives/carers, and this is the reason for interviewing them separately. However, if a patient is reluctant to participate in the qualitative interviews unless their relative/carer is in attendance, then it will be permitted.

The interview topic guide will be reviewed and refined in collaboration with a group of patient and public representatives, who will also review the patient information leaflets. Patients will be asked about participating in TILT, including any problems they encountered, and any areas where the reality of trial participation differed from their expectations. Participants who received OK-423 will be asked about their experience. Participants who declined any part of TILT will be asked to discuss their reasons.

In addition, the following topics will be covered during the interviews:

- The TwiC design will be explained. Do participants find this methodology acceptable?
- Were the study follow up requirements manageable?
- What are their feelings about future trials and what factors might affect their participation?

Interviews will last approximately 1 hour. At the end of the interview, participants will be given the contact details of the local mesothelioma specialist nurse and the lead trial nurse in case any further support is required. We do not anticipate that the interviews will cause particular distress, but we are aware that talking about personal experiences can be upsetting. Therefore, the study processes for provision of support to a participant who becomes distressed will be used. For interviews, this comprises the interviewer asking the patient whether they would like to stop the interview at that point and acting according to their wishes. If the patient wishes to continue then the interviewer will do so but check again later in the interview. All participants who express distress during interview will be offered referral to an NHS clinical psychologist and all participants in the study as a whole will be provided with a list of useful further contacts for support. The interviewer (Dr Bibby) is an experienced clinician who has worked with this patient group for 8 years.

Interviews will be digitally audio-recorded, transcribed, anonymised and uploaded to QSR NVivo qualitative analysis software. Data will be analysed using thematic analysis, adopting an inductive, semantic and realist approach.^96^ Audio-recordings will be listened to and transcripts read twice for familiarisation, then inductively coded to develop an initial code list. A subset of interviews will be independently double-coded by an independent qualitative researcher and code-lists compared, refined and applied to the dataset, with further refinements as required. Coded segments of transcripts will be grouped into categories, and data explored to identify connections between categories and to develop a descriptive account of the dataset as a whole.

## Methodology for focus group sessions

Because previous qualitative research has highlighted the stoical nature of people with MPM,^85^ and because it is anticipated that relatives/carers are likely to have had different experiences of MPM and TILT, we will also undertake focus group sessions with participants’ relatives/carers. It is hoped that this will provide additional insight into the overall experience of MPM and participation in a research trial.

When trial participants are invited to join the qualitative interviews, they will also be asked whether they agree to their relative/carer being invited to a focus group session about the research. The participant information sheet for the qualitative research will include information on both the individual participant interviews and the focus group sessions for relatives/carers. If participants agree for their relatives to be approached, they will be asked to provide the name of their relative/carer, and that person will be invited to participate in a forthcoming focus group session. The invitation will be delivered either in person, if the relative/carer has attended the appointment with the participant, or via an invitation letter given to the participant to take home. Participants who do not wish a relative/carer to be invited to a focus group will still be eligible to participate in the one-to-one qualitative interviews.

Focus groups will be separated based on the arm of the trial the participant associated with the relative/carer was in. This is because the experience of relatives/carers will be different depending on which arm of the trial the participant was in. Separating the focus groups will also reduce the risk of distress or conflict arising as result of relatives/carers discovering that different participants were offered different options (see Section 14.4 for further details). Where the number of relatives/carers associated with a particular trial experience is small, e.g. the relatives/carers of participants who were offered bacterial immunotherapy but declined it, individual qualitative interviews will be offered.

Focus groups will be held in a research building on hospital property, separate from clinical care facilities, or in a community building such as a village hall or private room in a pub or café. Because the relatives/carers of people with MPM may be anxious about leaving their relatives alone whilst they participate in the focus groups, every attempt will be made to hold the sessions at the same time and in the same location as an alternate activity for the patient e.g. patient support group or social activity.

Focus groups will be facilitated by Dr Bibby, with an administrator present to document the first comments made by each participant so they can be identified on the audio-recording later, and to take notes throughout the focus group session.

The topic guide for the focus groups will be designed by the research team, and reviewed and refined in collaboration with patient and public involvement. Attendees will initially be invited to share their experiences of having a relative participate in TILT. They will be encouraged to discuss any expectations or reservations they had prior to the research, and whether the reality of trial participation matched up to this. In addition, the following topics will be covered during the focus group sessions:

- Do they feel that trial participation was onerous for their relative?
- The TwiC design will be explained. Do they find it acceptable?
- How did they feel about their relative receiving OK432/BCG?
- Would they support their relative to participate in future research, and what factors might affect this?
- If their relative declined any aspect of the trial, how did they feel about it?

Sessions will last approximately 2 hours each. They will be audio-recorded, transcribed and analysed using the methodology outlined in section 10.3.

## Qualitative interviews with bereaved relatives

One of the eligibility criteria for participation in TILT is a predicted survival of greater than 3 months. This is to minimise the number of participants who die before completing trial follow up. However, previous clinical trials in this patient population have shown that the accuracy with which clinicians can predict survival is poor.^97^ Therefore it is possible that some participants may die before the qualitative research takes place.

We wish to give the relatives/carers of participants who died during the course of TILT the opportunity to discuss their experience and for their feelings to be heard. This group of bereaved relatives/carers are likely to have a unique experience of research participation at the end of life. For this reason we will seek ethical approval to write a letter of condolence to the bereaved relative/carer thanking them for their relative’s involvement in the research, and inviting them to take part in a one to one qualitative interview with Dr Anna Bibby. If they are interested in taking part in the qualitative interviews they will be asked to contact a member of the research team. If the research team does not hear from the relative/carer, there will be no further attempts to contact them.

If bereaved relatives wish to participate in qualitative interviews, they will be given the choice of where and when the interview is held. This is because they may prefer not to return to the hospital where their relative was treated. Additionally, if they wish to have a friend or family member present during the interview, this will be allowed. It is not anticipated that the interviews will be distressing in themselves, however it is appreciated that this will be a difficult and emotional time for relatives. Therefore, if a relative becomes distressed at any point the interviewer will ask them whether they would like to stop, and will terminate the interview if they say yes. If the relative wishes to continue, then the interviewer will do so but will check again later that they are OK to continue. All relatives who express significant distress during an interview will be offered referral to an NHS clinical psychologist and all participants in the study will be provided with a list of useful further contacts for support.

## Sample size for qualitative research

A sample of 20 TILT participants will be invited to undertake individual qualitative interviews. Participants will be purposively sampled from both arms of the trial, including those who completed follow-up, those who withdrew and those who declined any element of the study.

We anticipate at least 15 participants will accept the invitation to interview. Given the relatively homogeneous characteristics of people with MPM (85% men, 75% over the age of 65, industrial occupations), we anticipate this will be sufficient to achieve thematic saturation and the proposed sample size is in keeping with other successful qualitative studies.^2 98^

Four focus groups of approximately 6 relatives will be held (2 in Bristol and 2 in Oxford), yielding a sample size of at least 20. Given the similarity of experience that will be discussed in the focus groups (i.e. caring for a relative with MPM who has participated in a specific arm of TILT), it is anticipated that this will be sufficient to achieve thematic saturation.^98^

# PARTICIPANT WITHDRAWAL/NON-CONSENT

All participants will have provided written informed consent to trial follow-up, and to sample collection, storage and analysis where appropriate. Participants have the right to withdraw consent at any point. Withdrawal does not have to be justified and will not affect future or on-going care. In the event of withdrawal, any details available for the reason(s) should be recorded in the patient’s eCRF, and clarification on the nature of the withdrawal of consent, as outlined below, should be sought. Patients may still be classified as ‘alive’ or ‘dead’ at the end of their follow-up period, unless consent for clinical data use is withdrawn.

## Withdrawal of consent to all trial involvement

The participant withdraws consent for all trial involvement, including further data collection, sample storage and analysis, and the use of data already collected in the final trial analysis. Samples already taken and follow-up data should be destroyed as per local policy.

## Withdrawal of consent for further data collection

The participant withdraws consent for further follow-up visits and recording of clinical data. They maintain consent for blood and fluid samples already taken to be analysed, and for data already collected to be used in analyses.

## Withdrawal of consent for further data collection and use of existing data

The participant withdraws consent to further follow-up visits, recording of clinical data and the use of any clinical data already collected. They maintain consent for blood and fluid samples already taken to be analysed.

## Withdrawal of consent for sample analysis

The participant withdraws consent for existing blood and pleural fluid samples to be analysed, and for any data already obtained from these samples to be used in the final analysis. Samples and associated data should be destroyed in line with local policy. They maintain consent for ongoing trial follow-up, data collection and the use of this data in the final analysis.

## Loss to follow up

Loss to follow up will be minimised by diligent liaison with the patient, their oncology team and general practitioner (GP). Any loss to follow-up should be recorded on the participant withdrawal/loss to follow-up form. For participants moving from the area, every effort should be made for the participant to be followed up at another centre, or for follow up via their GP.

# ADVERSE EVENT MONITORING

## Definitions

*Adverse Event (AE)*

Adverse Events (AEs) are any untoward medical occurrence in a patient or clinical trial subject administered an investigational medicinal product (IMP), medical device or intervention and which does not necessarily have a causal relationship with this treatment. This includes any unfavourable and unintended signs, including abnormal laboratory results, symptoms or a disease temporarily associated with an investigational product.

AE must always be recorded on an eCRF and in the patient's medical notes.

*Adverse Reaction (AR)*

Adverse Reactions (ARs) are adverse events where there is evidence to suggest there is a causal relationship between the event and the IMP.

*Serious Adverse Event (SAE) or Serious Adverse Reaction (SAR)*

Serious Adverse Events (SAEs) are defined as any untoward medical occurrence(s) that results in:

- death
- real and immediate threat to life
- hospitalisation
- prolongation of existing hospitalisation
- persistent or significant disability or incapacity
- a congenital anomaly or birth defect
- other health event which in the opinion of the clinician is serious

*Serious Adverse Reaction (SAR)*

Any SAE that is classed as serious in nature and which is consistent with the information about the IMP set out in the summary of product characteristics (SmPC) for that product or listed as an expected reaction in the trial protocol.

*Suspected Unexpected Serious Adverse Reaction (SUSAR)*

Any SAE/SAR that is suspected to be caused by the IMP, but which is not consistent with the information available about the IMP set out in the SmPC for that product or listed as an expected reaction in the trial protocol. If the event is not listed as expected, or has occurred in a more serious form than anticipated, it should be considered a SUSAR.

## Expectedness

The population of patients involved in TILT is one in which a high number of adverse events are expected. Many of these will unrelated to IMP administration or trial-related procedures, but rather will be a direct consequence of the patient’s underlying malignancy. These expected AE do not need to be reported to the sponsor, even if they meet the criteria for an SAE.

Expected adverse events in these settings are:

- Death due to underlying malignancy
- Admission related to underlying malignancy
- Prolongation of inpatient hospital stay due to underlying malignancy

In addition to these expected AE, there are a number of AR that are well documented and regarded as normal reactions following intra-pleural OK432 or BCG administration. However, because neither IMP has been used in this context in this country before, and because TILT is a feasibility trial, all AR, including expected AR must be reported on eCRFs and, if they meet the criteria for SAR, should be reported to the sponsor in accordance with the guidance below.

## Causality

The relationship between the trial treatment and the adverse event will be graded as one of the following:

- Unrelated – Temporal relationship of the onset of the AE, relative to the administration of the product, is not reasonable or another cause can explain the occurrence.
- Unlikely – Temporal relationship of the onset of the AE, relative to the administration of the product, is likely to have another cause which can by itself explain the occurrence
- Possibly related – Temporal relationship of the onset of the event, relative to administration of the product, is reasonable but the even could have been due to another, equally likely cause.
- Probably related – Temporal relationship of the onset of the event, relative to administration of the product, is reasonable and the event is more likely to be explained by the product than any other cause.
- Definitely – Temporal relationship of the onset of the event, relative to administration of the product, is reasonable and there is no other cause to explain the event, or a re-challenge (if feasible) is positive.
- Not assessable - there is insufficient or incomplete evidence to make a clinical judgement of causality.

Any event that is assessed as possibly, probably or definitely related should be classified as an AR.

The assignment of causality to an adverse event or adverse reaction should be made by the investigator responsible for the care of the participant based on the definitions above. If any doubt about causality exists, the local investigator should inform the Trial Manager or Chief Investigator. The pharmaceutical company and/or other clinicians may be asked to advise in some cases. If there is disagreement regarding causality, the involved parties will discuss the case together. In the event that no agreement is reached, the MHRA will be informed of both points of view.

## Adverse events in TILT and ASSESS-meso participants

Adverse event data is collected at every visit, for all participants in ASSESS-meso and TILT. Whilst participants in ASSESS-meso will not experience any adverse reactions (AR) since they have not received an intervention, it is nonetheless possible that they may experience AEs or SAEs.

Although ASSESS-meso participants are not considered CTIMP participants, some will nonetheless be contributing ‘control’ data to the TILT trial. For clarity and transparency of reporting, all SAEs occurring in ‘control’ participants will be reported to the sponsor in accordance with the specifications below.

Furthermore, because TILT participants will return to follow up in ASSESS-meso following the end of trial, ongoing adverse events monitoring is required in case of any late adverse reactions that present after the 3 month trial period. Reporting of these adverse events will comply with the reporting procedure outlined below. In addition, trial-specific protocol 6b (TSP06b) will contain information on potential adverse reactions to OK432 and BCG, and advice on appropriate management. This TSP will continue to be available for TILT participants once they have returned to ASSESS-meso following the end of trial.

## Reporting procedures

All adverse events will be recorded in the patient’s medical notes and on eCRFs. Depending on the nature of the event the reporting procedures below should be followed. Any questions concerning adverse event reporting should be directed to the trial coordination centre in the first instance.

*Non-serious AE/AR*

All such toxicities, whether expected or not, should be recorded in the adverse events section of the relevant eCRF and on a separate adverse event log for each participant. Non-serious AE/AR do not need to be reported to the trial coordination centre.

*Expected AE*

Expected AE, as listed in section 12.2, should be recorded on the appropriate eCRF and on a separate adverse event log as above. These events do not need be reported as SAE (even if the criteria for such are met) unless the local Principal Investigator deems this necessary.

*Expected AR*

There are a number of expected AR related to both IMPs, including fever, tachycardia, tachypnoea and chest pain following administration. Non serious AR should be recorded on the appropriate eCRF and on a separate adverse event log as above. Serious AR, even if expected, should be reported to the trial coordinating centre and the sponsor in accordance with the specifications below.

*SAR/SAE*

In the case of SAE or SAR, an SAE form (Initial Safety Report Form, I/QMS/SOP/013a) should be completed and faxed/emailed to the trial coordination centre and to the sponsor within 24 hours. The SAE form asks for nature of event, date of onset, severity, corrective therapies given, outcome and causality (as detailed in sections 12.3 and 12.4).

The Principal Investigator should review all SAE forms, and state expectedness and relatedness to the trial. All forms must be signed by the PI (submission of the form by email from the PIs professional address will act as PI signature). Receipt of the form will be reviewed by a delegated member of North Bristol NHS Trust Research and Innovation Department (R&I) and logged on the R&I database. The report may be reviewed by a meeting of members of R&I and a clinical trial pharmacist may be consulting along with the CI, if deemed necessary.

Additional information should be sent within 48 hours if the reaction has not resolved at the time of reporting. Information not available at the time of reporting (such as test results) must be forwarded once available. If the event is ongoing, additional information should be submitted to R&I by fax using the SAE/SAR/SUSAR Follow Up Report Form for CTIMPs (RI/QMS/SOP/013b), available on the NBT website. There is no mandatory requirement regarding the frequency which follow-up reports should be submitted. As a minimum, a report should be submitted when the event resolves/ends.

Fatal or life threatening SAE/SAR should be reported on the day that the local centre is made aware of the event.

The exception is if the SAE/SAR is an expected event, as described in section 12.2, in which case an SAE form does not need to be completed. Expected SAE/SAR should be recorded on eCRFs and on adverse event forms, but do not need not to be reported unless the local Principal Investigator feels there is an important relationship to OK432/BCG.

*SUSAR*

SUSARs must be reported to R&I immediately using the Initial Report Form for CTIMPs (RI/QMS/SOP/013a). There are several reporting requirements for SUSARs. R&I will coordinate the submission of these reports to:

- The main REC (which granted approval for the trial to proceed)
- The MHRA via the eSUSAR electronic reporting system.

The following expedited reporting procedure will apply:

- Fatal or Life-threatening SUSARs - relevant bodies must be notified **as soon as possible** but no later than **7 calendar days** after the CI first has knowledge of the event. Any further information should be forwarded to these bodies within an additional **8 calendar days**.
- Non-fatal or non-life-threatening SUSARs - relevant bodies must be notified **as soon as possible** but no later than **15 calendar days** after the CI first has knowledge of the event. Any further information should be sought and a full report submitted **as soon as possible.**

If the event is ongoing, additional information should be submitted to R&I using the SAE/SAR/SUSAR Follow Up Report Form for CTIMPs (RI/QMS/SOP/013b), available on the NBT website. There is no mandatory requirement regarding the frequency which follow-up reports should be submitted. As a minimum, a report should be submitted when the event resolves/ends.

## Follow-up after adverse events

Participants experiencing SAEs or AEs will receive the appropriate medical treatment as per standard care. SAE/SAR/SUSAR should be followed up until resolution, including follow-up in ASSESS-meso if the event continues after the end of TILT. After recovery or once the event is considered stable, participants will be followed up in the normal trial or study schedule.

## Outcome grading of adverse events

Adverse events are graded as either: resolved, stabilised, on-going, resolved with sequelae or death.

## Contact details for adverse event reporting

Contact details for reporting SAEs and SUSARs are as follows:

NBT R&I office (within 24 hours)

Fax: 0117 414 9329

Email: [researchsponsor@nbt.nhs.uk](mailto:researchsponsor@nbt.nhs.uk)

A copy of all SAE forms must be sent to:

TILT Trial Administrator

Clinical Research Centre - Respiratory, Southmead Hospital, Bristol, BS10 5NB

Tel: 0117 414 8114 (Mon to Fri 08.00 – 16.00)

Fax: 0117 414 8149 (For the attention of TILT Trial administrator)

## Reference Safety Information

The following will be used as the Reference Safety Information (RSI) during the course of this trial:

- Summary of Product Characteristics for OK432(Piciban il)/
- Summary of Product Characteristics for BCG (OncoTice)/

If there are any updates made to the document described above, these will be reviewed by the Chief Investigator and Sponsor and a joint decision made whether the updated document will be submitted to the MHRA for use as the RSI in the trial

## Development Safety Update Report (DSUR)

In addition to the expedited reporting above, a Development Safety Update Report (DSUR) will be submitted once a year throughout the clinical trial or on request of a safety report to the sponsor, Competent Authority (MHRA), host NHS Trusts R+D departments and the REC via the Health Research Authority (HRA).

# STATISTICAL ANALYSIS

## Primary outcome measure

The primary outcome measure is feasibility, which will be assessed based on the following criteria:

- Recruitment rates to TILT
- Feasibility of identifying and randomising 12 eligible participants
- Acceptance rates for intra-pleural OK432/BCG following random selection
- Collection of data on participants who declined to receive OK432/BCG when offered it
- Collection of data on participants within ASSESS-meso who declined to be considered for future trials
- Number of participants in the control arm who were aware of the intervention arm
- Attrition rates and data completeness rates

In addition to feasibility data, qualitative interviews will be undertaken with participants and focus groups with their relatives/carers to explore the acceptability of trial processes. Analysis of the qualitative data is described in Section 10.3.

## Secondary outcome measures

Secondary outcome measures will collect exploratory data on the clinical efficacy of OK432 and BCG. This data will be used to decide the primary outcome for the subsequent full-scale trial, and to estimate the variance of the chosen outcome for a sample-size calculation. Potential outcomes include:

- Tumour response, based on CT appearances at baseline and 12 weeks ^42^
- Overall survival (OS), defined as time between date of diagnosis with MPM to date of death, censored 12 weeks after the final trial visit of the final participant
- Progression-free survival rates (PFSR) at 12 weeks
- Patient-reported chest pain and breathlessness, measured on visual analogue scales (VAS)
- Patient-reported quality of life, measured using the EQ-5D-5L health questionnaire
- Pleurodesis rates, defined as pleural fluid drainage of less than 50ml on 3 consecutive occasions, with <25% opacification on CXR or <250ml pleural fluid on thoracic ultrasound scanning (TUS)
- Time to pleurodesis, calculated from baseline assessment to date of pleurodesis
- Biomarker response assessed using serial serum and pleural mesothelin levels

Participants will be followed up until death, or end of trial (EoT) 12 weeks after the final trial visit of the final participant, whichever is sooner.

## Analysis plan

Basic descriptors will be used to summarise the characteristics of the overall trial population, each arm, and participants who decline any element of the trial. The characteristics of people who decline participation will be compared with the trial population using means, confidence intervals, chi-squared and unpaired T-tests. If possible, multivariable regression modelling will be undertaken to explore which factors were associated with non-acceptance of OK432/BCG.

Feasibility outcomes, including recruitment, randomisation and trial completion rates, will be described for the overall trial and for each arm. Secondary outcomes will be summarised for each arm based on randomisation, i.e. intention to treat. Per protocol and compliance averaged causal effects (CACE) analysis will be undertaken as secondary analyses for clinical efficacy outcomes.

Time to event data will be analysed using the Kaplan-Meier method. Cox proportional hazards modelling will be used to calculate hazard ratios for death and pleurodesis. Univariable modelling will be used, followed by multi-variable methods to control for potential confounders. The time-independent confounders to be considered are age, sex, performance status, tumour histology and whether chemotherapy was received. Time-dependent confounders that will be adjusted for are the time between diagnosis with MPM and baseline assessment, and length of time the IPC was in situ. The main analysis will include all patients. A priori sub-group analyses will stratify patients according to time since diagnosis (≥8 weeks versus <8 weeks) and whether chemotherapy was received.

Pleurodesis rates will be calculated for the whole population, and in the sub-group of patients who’s IPC had been in situ for less than 6 weeks at baseline assessment.

Outcomes with repeat measurements, e.g. PROMS and biomarker responses, will be analysed using regression modelling with adjustment for baseline values. Survival rates will be compared with national survival data, and survival rates from previous clinical trials of new treatments in MPM. Progression-free survival rates will be compared to validated values for acceptable clinical activity of new oncological agents in MPM.^99^

Statistical analysis will be undertaken using Stata (StataCorp LP) version 14, with support from YTU.

# ETHICAL CONSIDERATIONS

## Indwelling pleural catheters

The use of IPCs to manage malignant pleural effusions is standard practice in many centres in the UK and worldwide. Consequently there are no predicted ethical issues regarding their use in this study, especially since the majority of participants are expected to already have an IPC in situ. Participants without an existing IPC must be willing and able to have one inserted in order to be eligible for TILT.

## OK432

OK432, as used in the context proposed in this trial, is regarded as an IMP and the appropriate approval will be sought from the MHRA and HRA. .

Intra-pleural OK432 is an established treatment for malignant pleural effusions in Japan, South Korea and Taiwan. Since its launch in 1975 adverse event reporting has revealed no significant adverse reactions greater than grade 2 severity. Safety data of intra-pleural OK432 in people with MPM will be collected throughout TILT. Participants who are offered OK432 will be made aware of OK432’s unlicensed status in the UK, and will be asked to provide consent to receive an IMP.

## BCG

Intra-pleural BCG, as used in the context proposed in this trial, is regarded as an IMP and the appropriate approval will be sought from the MHRA and HRA

Intra-pleural BCG has previously been used in patients with lung cancer following thoracic surgery. As well as mild, self-limiting systemic inflammatory symptoms, such as fevers, malaise and fatigue, the most common complication following BCG administration was would infection, bacterial or BCG pleural infection and chronic chest pain. Many of these adverse events are likely to relate to the delivery of a live agent into an area that had recently undergone a major surgical intervention. For this reason, recent thoracic surgery or intervention is an exclusion criteria for TILT.

Disseminated BCG is a rare but important risk associated with both intra-pleural and intra-vesical BCG delivery. Participants will be monitored for signs of disseminated BCG at every trial visit. TSP06b will include guidance on the diagnosis and management of disseminated BCG infection in TILT participants. Participants who are offered BCG will be made aware of all the associated risks, and will be asked to provide consent to receive it.

## Qualitative interviews

There is a possibility that the qualitative interviews may raise sensitive or upsetting issues in this population of patients with an incurable disease. However, consultation with patients has identified enthusiasm to discuss their experiences with clinicians. Interview content will be reviewed by a specific mesothelioma patient and public engagement group to ensure it is acceptable. Additionally, all participants will be given the contact details of a mesothelioma specialist nurse in case they require further support following the qualitative interviews. Anyone who finds the interviews particularly distressing will be offered referral to an NHS clinical psychologist.

## Focus group sessions

Existing qualitative research has demonstrated that the responses of partners, carers and relatives of people with MPM can differ significantly from that of the patient. Previous qualitative work with relatives revealed intense passion and anger, and the feeling that their role was to “fight for their loved ones, [who] were too ill to fight for themselves”.^85^ It is anticipated, therefore, that the focus group sessions may be more animated and impassioned than the individual participant interviews. All participants in the focus groups will be given the contact details of a mesothelioma specialist nurse in case they require further support, and anyone who finds the interviews particularly distressing will be offered referral to an NHS clinical psychologist.

It is recognised that the experiences of relatives/carers will be different depending on which arm of TILT their relative participated in. Therefore, if the focus groups were made up of a mixed group of relatives/carers, there is a risk of potential conflict or distress. For example, the relative of a participant in the observational arm may resent the partner of a participant who was offered OK432 or BCG. Conversely, people whose partners were not offered bacterial immunotherapy may feel angry towards the partner of a participant who was offered OK432 or BCG but declined it. Finally, the wife of a patient who declined immunotherapy may find it upsetting to hear the experiences of people who received it, particularly if the overall experience was positive, and may end up become angry at their partner for declining treatment.

For these reasons, separate focus group sessions will be held based on which arm of the study the associated participant of the relative/carer participated in. Where the number of relatives/carers associated with a particular trial experience is small, e.g. the relatives/carers of participants who were offered OK432 or BCG but declined it, individual qualitative interviews will be offered.

## Qualitative interviews with bereaved relatives

Ethical approval will be sought to contact the primary relative/carer of participants who have died during the course of TILT. The research team will send a letter to the bereaved person, thanking them for their relative’s participation in the research and inviting them to take part in a one to one qualitative interview with Dr Anna Bibby.

It is recognised that for the bereaved person, this will be an emotionally difficult time, and they may not wish to have any further interactions with the research (or clinical) team. Relatives who do not respond to the letter will not be contacted again.

If bereaved relatives do wish to participate in qualitative interviews, they will be given the choice of where and when the interview is held. This is because they may prefer not to return to the hospital where their relative was treated. Additionally, if they wish to have a friend or family member present during the interview, this will be allowed. It is not anticipated that the interviews will be distressing in themselves, however it is appreciated that this will be a difficult and emotional time for relatives. Therefore, if a relative becomes distressed at any point the interviewer will ask them whether they would like to stop, and will terminate the interview if they say yes. If the relative wishes to continue, then the interviewer will do so but will check again later that they are OK to continue. All relatives who express significant distress during an interview will be offered referral to an NHS clinical psychologist and all participants in the study will be provided with a list of useful further contacts for support.

## Consent and withdrawal

Participants must have full capacity to provide consent for the trial, as defined by the Mental Health Capacity Act 2005. To be eligible for TILT, participants must have already provided written, informed consent to join ASSESS-meso. The ASSESS-meso consent form will specifically refer to the use of participants’ data to identify additional trials they may be eligible for, and that they may be randomly selected to be invited to join such trials in future. Participants who have not consented to this element will not be eligible for TILT.

In this way, random selection can be undertaken on all eligible participants without requiring further TILT-specific consent. Participants who are not randomly selected to receive bacterial immunotherapycontinue observational follow up in ASSESS-meso, without undergoing any TILT-related interventions. Consequently they are not required to provide further consent.

Participants who are randomly selected to receive OK432 or BCG will be given detailed information about TILT and about the investigational medicinal product. They will have the opportunity to discuss the trial further with a member of the trial team. Participants will generally have 24 hours ‘thinking time’ to consider enrolling in the trial, although it is recognised that clinical circumstances may sometimes make this impossible. Participants will be asked to provide written, informed consent to participate in TILT, and for the collection of information about their care and collection of subsequent data sheets. Participants will be assured that their data will be stored securely and anonymously (see sections 14.5 and 14.6).

The right of the participant to refuse to participate in the trial without giving a reason is respected. However, specific ethical approval will be obtained to ask patients who decline trial participation to briefly give their reasons. In addition, these participants will be invited to participate in qualitative interviews exploring the acceptability of the research to them and their family members/carers.

Similarly, participants remain free to withdraw at any time from trial follow-up without giving reasons and without prejudicing their further treatment. These participants will remain within the trial for the purpose of follow-up unless the participant has specifically withdrawn consent for such follow-up. See section 11 for further information about withdrawal of consent.

## Data Management

A data management plan for the TWIC will be developed. The data management plan will identify and clarify:

- What data will be collected
- Who will collect the data
- How the data will be collected, managed, retained and archived.
- Who would have access to data and database, in accordance with Data Protection Act 1998 and participants confidentiality
- How data will be clarified and validated
- Who can clarify data within databases
- Who can approve data collection form
- How databases will be validated and commissioned.
- The process by which the study design may be amended
- Who can approve amendments to the study design.

## Confidentiality

Trial staff will ensure that participants’ anonymity is upheld by secure handling and storage of patient information at trial centres. All trial documents will be stored securely, and will be accessible only to trial staff and authorised personnel. Data will be collected and retained in accordance with the Data Protection Act 1998.

Participants’ personal data will be treated as strictly confidential. To maintain anonymity, only participants’ trial number, initials and date of birth will be recorded on trial documentation. Participants’ data will be stored on an administrative Access database. The database will be stored on a secure University of Bristol server, and will be protected by a combination of file permissions and passwords. Only authorised trial team members will have access to participants’ personal data. The local researcher at each centre will be responsible for entering personal participant information into the database and allocating their trial number.

Qualitative interviews will be audio-recorded on an encrypted device. Files will be stored electronically, identified by trial number only, on a University of Bristol secure server, in an access-limited, password protected folder.

## Data security

Anonymised trial data will be stored using a bespoke, online, secure database created for the trial by YTU. Researchers at trial centres will be able to enter participant data onto the database via eCRFs. The database will include real-time queries to reduce missing or impossible data and optimise data quality.

Original paper forms (e.g. consent forms) will be retained at each trial centre, in secure storage, and will be made available on request to the sponsor for audit purposes. A copy of the consent form will be sent to the co-ordinating trial centre (North Bristol NHS Trust) on enrolment. Paper checklists are not classified trial documents and can be filed in the patients’ notes to act as source data or stored or destroyed at the respective study centre. At the end of the trial, all trial documents, will be sent to NBT for archiving. Archiving will take place in accordance with NBT’s archiving SOP.

Electronic records will be protected using a combination of passwords and file permissions. Data procedures will adhere to the Data Protection Act 2000. Electronic data will be retained and, at the end of the trial, archived in line with Trust policy. With participants’ consent, electronic research data will be stored indefinitely and made available for future analysis.

## Data sharing

In line with NIHR guidance which encourages the sharing of anonymised datasets, we will be seeking consent from participants for their data to be shared with other researchers. This is in anticipation that data sharing and access to anonymised datasets may become mandatory in the coming years.

Participants will be asked on the consent form if they are willing for their anonymised data to be shared with other researchers in the future to support further research. The data of participants who agree to this will be stored on the University of Bristol Research Data Storage Facility at the end of the trial. This data will then be shared, via the University of Bristol Research Data Repository, with other researchers once a Data Access Agreement has been signed by an institutional signatory. Participants who decline to have their data shared can still participate in the study; their data will be removed from the dataset prior to archiving for potential sharing.

## Ethical approval

The trial protocol will be submitted to the Health Research Authority (HRA) for Research Ethics Committee (REC) approval. The trial consent form, participant information sheets, qualitative interview topic guide, focus group invitations, list of contacts and letter to GP informing them of participation in the trial will also be submitted to the HRA for approval. Full HRA approval will be in place before the trial commences.

# REGULATORY APPROVAL

The study will be performed subject to favourable opinion/ authorisation/permission or equivalent from all necessary regulatory and other bodies. This includes but is not limited to REC, MHRA, HRA, NHS trusts.

## MHRA approval

Clinical trials authorisation (CTA) will be obtained before the trial commences.

## NIHR portfolio registration

The trial will be registered on the NIHR UK Central Portfolio Management System, previously known as the UK Clinical Research Network. The registration specialty will be Respiratory. The trial will be accessible to the public via the Clinical Trials Gateway.

## EudraCT registration

The trial will be registered on the EudraCT database and will be issued with a unique EudraCT identification number.

## Research Governance Statement

## This study will be conducted in accordance with:

## The Medicines for Human Use (Clinical Trials) Regulations 2004 and subsequent amendments

## International Conference for Harmonisation of Good Clinical Practice (ICH GCP) guidelines.

# TRIAL INFRASTRUCTURE

## Monitoring and Quality control

Where applicable, a random sample of 10% of CRFs will be checked, by the trial Research Team or R&I monitor, against entries within the database and with the source data for quality purposes. The percentage checked will be increased if a significant error rate is found. The data from the first patient recruited at a new site will be reviewed. This may include consent records, safety data and primary endpoint data.

The trial will be monitored in accordance with NBT Monitoring SOP. All trial-related documentation will be made available, on request, for monitoring and audit purposes to the sponsor, the Research Ethics Committee, MHRA or other licensed body. The monitoring plan will be developed and agreed by the sponsor.

## Trial management group

The trial management group is responsible for the day-to-day management of the trial. The team is responsible for all aspects of the project (such as recruitment rate, budget management, protocol adherence, etc.) and for ensuring appropriate action is taken to safeguard trial participants and the quality of the study.

The trial management group consists of:

- Prof. Nick Maskell, Chief Investigator & Principal Investigator for Bristol
- Dr Anna Bibby, Trial Manager
- Mrs Helen-Lewis White, Sponsor Representative
- Mrs Alia Ataya, R&I representative
- Mrs Natalie Zahan-Evans, lead trial nurse in Bristol
- Dr Emma Keenan, trial administrator

Identification of participants, delivery of OK432/BCG and trial assessment visits will be undertaken at the patient’s local trial centre by members of the local trial team. Drainage of participants’ IPCs will take place in the community and will be performed by community nurses, lung cancer specialist nurses, trial nurses or family members/carers, all of whom must be appropriately trained.

The Academic Respiratory Unit at NBT will have responsibility for trial authorisation, ensuring Good Clinical Practice (GCP) certification and overall trial conduct, data integrity, data checking and database integrity.

Randomisation will be undertaken using a centralised, concealed, computerised system, embedded within the trial database.

## Trial steering committee (TSC)

The TSC will consist of independent members and researchers working on the trial. The role of the TSC is to provide overall supervision and monitor the progress of the trial to ensure that it is being conducted in accordance with the protocol, relevant regulations and the principles of GCP. The Sponsor will be represented at TSC meetings but may choose to devolve this responsibility to one of the people named below.

The TSC will meet at before the start of the trial and regularly throughout the trial period, with 6 monthly meetings as a minimum. TSC meetings may be convened more frequently depending on recruitment, safety concerns or any other issues relating to trial management,

The TSC will comprise:

Independent chair Dr John Harvey

Chief Investigator Prof Nick Maskell

Trial Co-ordinator Dr Anna Bibby

Principal Investigator (Oxford) Dr Najib Rahman

Specialist Nurse Advisor Ms Deborah Walton

Independent member Dr Charlie Comins

Patient/carer representative Helen Day

## Data monitoring committee

The TSC will act as a data monitoring committee. All SAE, SAR and SUSAR will be reviewed by the TSC. If there are significant concerns about the safety of the trial, the TSC will have the capacity to stop the trial early. However, since this is a feasibility study, no interim analysis is planned and the TSC will not have the capacity to close the trial based on exploratory clinical outcomes.

# FUNDING & INDEMNITY

## Funding

This trial has been funded, in its entirety, by a National Institute of Health Research (NIHR) Doctoral Research Fellowship awarded to Dr Anna Bibby (reference DRF-2016-09-065).

## Indemnity

The sponsor for this trial is North Bristol NHS Trust, and insurance will be provided by the sponsor. For NHS sponsored research, such as this trial, HSG(96)48 reference number 2 applies. If there is negligent harm during the clinical trial when the NHS owes a duty of care to the person harmed, NHS indemnity covers NHS staff, medical academic staff with honorary contracts, and those conducting the trial. NHS indemnity does not offer no-fault compensation and is unable to agree in advance to pay compensation for non-negligent harm.

Out with the trial, any hospital or other healthcare organisation responsible for the clinical care of study participants will have an equivalent arrangement in place in respect to indemnity and/or compensation for negligent harm arising during the clinical treatment of patients to whom they owe a duty of care.

# REFERENCES

1. Peake MD BP, Woolhouse I, et al on behalf of the National Lung Cancer Audit Project Team. National Lung Cancer Audit Report 2014, mesothelioma. Report for the period 2008–2012 <http://www.hscic.gov.uk/media/15038/Mesothelioma-audit/pdf/EMBARGOEDTO120914_NLCA_Meso_Report_final.pdf>: Health & Social Care Information Centre; 2014 [accessed 21/1/15 2015.

2. Beckett P, Edwards J, Fennell D, et al. Demographics, management and survival of patients with malignant pleural mesothelioma in the National Lung Cancer Audit in England and Wales. *Lung Cancer* 2015;88(3):344-8.

3. Hodgson J, McElvenny D, Darnton A, et al. The expected burden of mesothelioma mortality in Great Britain from 2002 to 2050. *British Journal of Cancer* 2005;92(3):587.

4. Vogelzang NJ, Rusthoven JJ, Symanowski J, et al. Phase III study of pemetrexed in combination with cisplatin versus cisplatin alone in patients with malignant pleural mesothelioma. *J Clin Oncol* 2003;21(14):2636-44.

5. Yates D, Corrin B, Stidolph P, et al. Malignant mesothelioma in south east England: clinicopathological experience of 272 cases. *Thorax* 1997;52(6):507-12.

6. Herndon JE, Green MR, Chahinian AP, et al. Factors Predictive of Survival Among 337 Patients With Mesothelioma Treated Between 1984 and 1994 by the Cancer and Leukemia Group B. *Chest* 1998;113(3):723-31. doi: <http://dx.doi.org/10.1378/chest.113.3.723>

7. Ribak J, Selikoff IJ. Survival of asbestos insulation workers with mesothelioma. *British Journal of Industrial Medicine* 1992;49(10):732-35.

8. Boon T, van der Bruggen P. Human tumor antigens recognized by T lymphocytes. *The Journal of Experimental Medicine* 1996;183(3):725-29.

9. Dunn GP, Old LJ, Schreiber RD. The immunobiology of cancer immunosurveillance and immunoediting. *Immunity* 2004;21(2):137-48.

10. Lew F, Tsang P, Holland JF, et al. High frequency of immune dysfunctions in asbestos workers and in patients with malignant mesothelioma. *J Clin Immunol* 1986;6(3):225-33.

11. Hegmans JP, Hemmes A, Hammad H, et al. Mesothelioma environment comprises cytokines and T-regulatory cells that suppress immune responses. *European Respiratory Journal* 2006;27(6):1086-95.

12. Leigh RA, Webster I. Lymphocytic infiltration of pleural mesothelioma and its significance for survival. *SAMJ, S Afr med j* 1982;61(26):1007-9.

13. Suzuki K, Kadota K, Sima CS, et al. Chronic inflammation in tumor stroma is an independent predictor of prolonged survival in epithelioid malignant pleural mesothelioma patients. *Cancer Immunol Immunother* 2011;60(12):1721-8.

14. Anraku M, Cunningham KS, Yun Z, et al. Impact of tumor-infiltrating T cells on survival in patients with malignant pleural mesothelioma. *J Thorac Cardiovasc Surg* 2008;135(4):823-9.

15. Yamada N, Oizumi S, Kikuchi E, et al. CD8+ tumor-infiltrating lymphocytes predict favorable prognosis in malignant pleural mesothelioma after resection. *Cancer Immunol Immunother* 2010;59(10):1543-9.

16. Calabro L, Morra A, Fonsatti E, et al. Tremelimumab for patients with chemotherapy-resistant advanced malignant mesothelioma: an open-label, single-arm, phase 2 trial. *Lancet Oncology* 2013;14(11):1104-11.

17. Calabrò L, Morra A, Fonsatti E, et al. Efficacy and safety of an intensified schedule of tremelimumab for chemotherapy-resistant malignant mesothelioma: an open-label, single-arm, phase 2 study. *Lancet Respiratory Medicine* 2015;3(4):301-09. doi: <http://dx.doi.org/10.1016/S2213-2600(15)00092-2>

18. P2.08-010 Phase II Study of the Anti-PD-1 Antibody Pembrolizumab in Patients with Malignant Mesothelioma. 16th World Conference on Lung Cancer; 2015; Denver, USA. Journal of Thoracic Oncology.

19. Sensenig D, Rossi N, Ehrenhaft J. Results of the surgical treatment of bronchogenic carcinoma. *Surgery, Gynecology & Obstetrics* 1963;116:279-84.

20. Takita H. Effect of postoperative empyema on survival of patients with bronchogenic carcinoma. *J Thorac Cardiovasc Surg* 1970;59(5):642.

21. Ruckdeschel JC, Codish SD, Stranahan A, et al. Postoperative empyema improves survival in lung cancer: documentation and analysis of a natural experiment. *New England Journal of Medicine* 1972;287(20):1013-17.

22. Virkkula L, Kostiainen S. Postpneumonectomy empyema in pulmonary carcinoma patients. *Scandinavian Journal of Thoracic and Cardiovascular Surgery* 1970;4(3):267-70.

23. Bibby AC, Clive AO, Slade GC, et al. Survival in Patients With Malignant Pleural Effusions Who Developed Pleural Infection: A Retrospective Case Review From Six UK Centers. *Chest* 2015;148(1):235-41. doi: 10.1378/chest.14-2199

24. McKneally M, Maver C, Kausel H, et al. Regional immunotherapy with intrapleural BCG for lung cancer. *J Thorac Cardiovasc Surg* 1976;72(3):333-38.

25. McKneally M, Maver C, Lininger L, et al. Four-year follow-up on the Albany experience with intrapleural BCG in lung cancer. *J Thorac Cardiovasc Surg* 1981;81(4):485-92.

26. Ruckdeschel JC, McKneally MF, Baxter DH, et al. Regional immunotherapy has a detrimental effect on the response to combined irradiation and chemotherapy in locally advanced non-small cell bronchogenic carcinoma. *Cancer Immunol Immunother* 1981;11(4):277-82.

27. Bakker W, Nijhuis-Heddes J, van der Velde E. Post-operative intrapleural BCG in lung cancer: a 5-year follow-up report. *Cancer Immunol Immunother* 1986;22(2):155-59.

28. Bakker W, Nijhuis-Heddes JM, de la Rivière AB, et al. Complications of postoperative intrapleural BCG in lung cancer. *Annals of Thoracic Surgery* 1982;33(3):267-72.

29. Ostrowski M, Priestman T, Houston R, et al. A randomized trial of intracavitary bleomycin and Corynebacterium parvum in the control of malignant pleural effusions. *Radiotherapy and Oncology* 1989;14(1):19-26.

30. Millar J, Hunter A, Horne N. Intrapleural immunotherapy with Corynebacterium parvum in recurrent malignant pleural effusions. *Thorax* 1980;35(11):856-58.

31. Webb H, Oaten S, Pike C. Treatment of malignant ascitic and pleural effusion with Corynebacterium parvum. *British Medical Journal* 1978;1(6109):338-40.

32. Casali A, Gionfra T, Rinaldi M, et al. Treatment of malignant pleural effusions with intracavitary Corynebacterium parvum. *Cancer* 1988;62(4):806-11.

33. McLeod D, Calverley P, Millar J, et al. Further experience of Corynebacterium parvum in malignant pleural effusion. *Thorax* 1985;40(7):515-18.

34. Rossi GA, Felletti R, Balbi B, et al. Symptomatic Treatment of Recurrent Malignant Pleural Effusions with Intrapleurally Administered Corynebacterium parvum: Clinical Response Is Not Associated with Evidence of Enhancement of Local Cellular-Mediated Immunity 1–3. *American Review of Respiratory Disease* 1987;135(4):885-90.

35. Uchida A, Micksche M. Lysis of fresh human tumor cells by autologous peripheral blood lymphocytes and pleural effusion lymphocytes activated by OK432. *Journal of the National Cancer Institute* 1983;71(4):673-80.

36. Luhr KT, Yang PC, Kuo SH, et al. Comparison of OK‐432 and mitomycin C pleurodesis for malignant pleural effusion caused by lung cancer. A randomized trial. *Cancer* 1992;69(3):674-79.

37. Sakamoto J, Teramukai S, Watanabe Y, et al. Meta-analysis of adjuvant immunochemotherapy using OK-432 in patients with resected non-small-cell lung cancer. *Journal of Immunotherapy* 2001;24(3):250-56.

38. Masuno T, Kishimoto S, Ogura T, et al. A comparative trial of LC9018 plus doxorubicin and doxorubicin alone for the treatment of malignant pleural effusion secondary to lung cancer. *Cancer* 1991;68(7):1495-500.

39. Ren S, Terman DS, Bohach G, et al. Intrapleural staphylococcal superantigen induces resolution of malignant pleural effusions and a survival benefit in non-small cell lung cancer. *CHEST Journal* 2004;126(5):1529-39.

40. Lansley SM, della Vergiliana V, Julius F, et al. A commercially available preparation of Staphylococcus aureus bio‐products potently inhibits tumour growth in a murine model of mesothelioma. *Respirology* 2014;19(7):1025-33.

41. Relton C, Torgerson D, O’Cathain A, et al. Rethinking pragmatic randomised controlled trials: introducing the “cohort multiple randomised controlled trial” design. *British Medical Journal* 2010;340:c1066.

42. Byrne MJ, Nowak AK. Modified RECIST criteria for assessment of response in malignant pleural mesothelioma. *Annals of Oncology* 2004;15(2):257-60.

43. Zalcman G, Mazieres J, Margery J, et al. Bevacizumab for newly diagnosed pleural mesothelioma in the Mesothelioma Avastin Cisplatin Pemetrexed Study (MAPS): a randomised, controlled, open-label, phase 3 trial. *The Lancet*;387(10026):1405-14. doi: <http://dx.doi.org/10.1016/S0140-6736(15)01238-6>

44. Cornelissen R, Scherpereel A, Pietro AD, et al. Randomised, Double-blind, Placebo-controlled Study of Tremelimumab for Second- and Third-line Treatment of Unresectable Pleural or Peritoneal Mesothelioma. American Society of Clinical Oncology. Chicago, USA, 2016.

45. Clive AO, Jones HE, Bhatnagar R, et al. Interventions for the management of malignant pleural effusions: a network meta‐analysis. *The Cochrane Library* 2016

46. RYOMA Y, MORIYA Y, OKAMOTO M, et al. Biological Effect of OK-432 (Picibanil) and Possible Application to Dendritic Cell Therapy. *Anticancer Research* 2004;24(5C):3295-302.

47. Adams S. Toll-like receptor agonists in cancer therapy. *Immunotherapy* 2009;1(6):949-64. doi: 10.2217/imt.09.70

48. Fuge O, Vasdev N, Allchorne P, et al. Immunotherapy for bladder cancer. *Research and Reports in Urology* 2015;7:65-79. doi: 10.2147/RRU.S63447

49. Sokal JE, Aungst CW, Han T. Effect of BCG on delayed hypersensitivity responses of patients with neoplastic disease. *International Journal of Cancer* 1973;12(1):242-49. doi: 10.1002/ijc.2910120125

50. Ratliff TL, Ritchey JK, Yuan JJJ, et al. T-Cell Subsets Required for Intravesical BCG Immunotherapy for Bladder Cancer. *The Journal of Urology* 1993;150(3):1018-23. doi: <https://doi.org/10.1016/S0022-5347(17)35678-1>

51. Brandau S, Riemensberger J, Jacobsen M, et al. NK cells are essential for effective BCG immunotherapy. *International Journal of Cancer* 2001;92(5):697-702. doi: 10.1002/1097-0215(20010601)92:5<697::AID-IJC1245>3.0.CO;2-Z

52. Morales A, Eidinger D, Bruce AW. Intracavitary Bacillus Calmette-guerin in the Treatment of Superficial Bladder Tumors. *The Journal of Urology* 1976;116(2):180-82. doi: <https://doi.org/10.1016/S0022-5347(17)58737-6>

53. Sylvester RJ, van der Meijden APM, Lamm DL. Intravesical Bacillus Calmette-Guerin Reduces the Risk of Progression in Patients with Superficial Bladder Cancer: A Meta-analysis of the Published Results of Randomized Clinical Trials. *The Journal of Urology* 2002;168(5):1964-70. doi: <https://doi.org/10.1016/S0022-5347(05)64273-5>

54. Sylvester RJ, van der Meijden APM, Witjes JA, et al. BACILLUS CALMETTE-GUERIN VERSUS CHEMOTHERAPY FOR THE INTRAVESICAL TREATMENT OF PATIENTS WITH CARCINOMA IN SITU OF THE BLADDER: A META-ANALYSIS OF THE PUBLISHED RESULTS OF RANDOMIZED CLINICAL TRIALS. *The Journal of Urology* 2005;174(1):86-91. doi: <https://doi.org/10.1097/01.ju.0000162059.64886.1c>

55. Shelley M, Kynaston H, Court J, et al. A systematic review of intravesical bacillus Calmette‐Guérin plus transurethral resection vs transurethral resection alone in Ta and T1 bladder cancer. *BJU international* 2001;88(3):209-16.

56. Han RF, Pan JG. Can intravesical bacillus Calmette-Guérin reduce recurrence in patients with superficial bladder cancer? A meta-analysis of randomized trials. *Urology* 2006;67(6):1216-23.

57. Pan J, Liu M, Zhou X. Can intravesical bacillus Calmette-Guérin reduce recurrence in patients with non-muscle invasive bladder cancer? An update and cumulative meta-analysis. *Frontiers of medicine* 2014;8(2):241-49.

58. Prescott S, James K, Hargreave TB, et al. Intravesical Evans Strain BCG Therapy: Quantitative Immunohistochemical Analysis of the Immune Response within the Bladder Wall. *The Journal of Urology* 1992;147(6):1636-42. doi: <https://doi.org/10.1016/S0022-5347(17)37668-1>

59. Morton DL, Eilber FR, Holmes EC, et al. BCG Immunotherapy of Malignant Melanoma: Summary of a Seven-year Experience. *Annals of Surgery* 1974;180(4):635-41.

60. Blank C, Lorigan PC, Schachter J, et al. Pembrolizumab versus ipilimumab for advanced melanoma: final overall survival analysis of KEYNOTE-006. *Melanoma Research* 2016

61. Hodi FS, Chesney J, Pavlick AC, et al. Combined nivolumab and ipilimumab versus ipilimumab alone in patients with advanced melanoma: 2-year overall survival outcomes in a multicentre, randomised, controlled, phase 2 trial. *Lancet Oncology* 2016;17(11):1558-68.

62. Postow MA, Chesney J, Pavlick AC, et al. Nivolumab and ipilimumab versus ipilimumab in untreated melanoma. *New England Journal of Medicine* 2015;2015(372):2006-17.

63. Alley EW, Molife LR, Santoro A, et al. Abstract CT103: Clinical safety and efficacy of pembrolizumab (MK-3475) in patients with malignant pleural mesothelioma: Preliminary results from KEYNOTE-028. *Cancer Res* 2015;75(15 Supplement):CT103-CT03. doi: 10.1158/1538-7445.am2015-ct103

64. Iles P, Shore D, Langman M, et al. Intrapleural BCG in operable lung cancer. Adjuvant Therapies and Markers of Post-Surgical Minimal Residual Disease II: Springer 1979:292-96.

65. Jansen HM, The TH, Orie NG. Adjuvant immunotherapy with BCG in squamous cell bronchial carcinoma. *Thorax* 1980;35(10):781-7.

66. Maver C, Kausel H, Lininger L, et al. Intrapleural BCG immunotherapy of lung cancer patients. *Recent Results Cancer Res* 1982;80:227-31.

67. Yasumoto K, Manabe H, Yanagawa E, et al. Nonspecific adjuvant immunotherapy of lung cancer with cell wall skeleton of Mycobacterium bovis Bacillus Calmette-Guerin. *Cancer Res* 1979;39(8):3262-7.

68. Yamamura Y, Sakatani M, Ogura T, et al. Adjuvant immunotherapy of lung cancer with BCG cell wall skeleton (BCG-CWS). *Cancer* 1979;43(4):1314-9.

69. Gail MH. A placebo-controlled randomized double-blind study of adjuvant intrapleural BCG in patients with resected T1N0, T1N1, or T2N0 squamous cell carcinoma, adenocarcinoma, or large cell carcinoma of the lung. LCSG Protocol 771. *Chest* 1994;106(6 Suppl):287S-92S.

70. Law M, Lam W, Studdy P, et al. Complications of intrapleural BCG in the treatment of operable non-small-cell bronchial carcinoma. *British journal of diseases of the chest* 1982;76:151-56.

71. Lowe J, Shore D, Iles P, et al. Intrapleural BCG in operable lung cancer. *The Lancet* 1980;315(8158):11-14.

72. STANLEY K. IMMUNOSTIMULATION WITH INTRAPLEURAL BCG AS ADJUVANT THERAPY IN RESECTED NON-SMALL CELL LUNG-CANCER. *Cancer* 1986;58(11):2411-16.

73. Holmes EC, Hill LD, Gail M. A randomized comparison of the effects of adjuvant therapy on resected stages II and III non-small cell carcinoma of the lung. The Lung Cancel Study Group. *Annals of Surgery* 1985;202(3):335-41.

74. Bartlett GL, Kreider JW, Purnell DM, et al. Augmentation of immunity to line 10 hepatoma by BCG. Comparison of different BCG preparations. *Cancer* 1980;46(3):488-96. doi: 10.1002/1097-0142(19800801)46:3<488::AID-CNCR2820460312>3.0.CO;2-M

75. Willmott N, Pimm M, Baldwin R. Quantitative Comparison of BCG Strains and Preparations in Immunotherapy of a Rat Sarcoma 2. *Journal of the National Cancer Institute* 1979;63(3):787-95.

76. Cheung M, Talarchek J, Schindeler K, et al. Further evidence for germline BAP1 mutations predisposing to melanoma and malignant mesothelioma. *Cancer genetics* 2013;206(5):206-10.

77. Husain AN, Colby T, Ordonez N, et al. Guidelines for pathologic diagnosis of malignant mesothelioma: 2012 update of the consensus statement from the International Mesothelioma Interest Group. *Archives of Pathology and Laboratory Medicine* 2012;137(5):647-67.

78. BastJr R, Zbar B, Borsos T. BCG and cancer (first of two parts). *N Engl J Med* 1974;290(25):1413-20.

79. Burbach JM, Verkooijen HM, Intven M, et al. RandomizEd controlled trial for pre-operAtive dose-escaLation BOOST in locally advanced rectal cancer (RECTAL BOOST study): study protocol for a randomized controlled trial. *Trials* 2015;16(1):1.

80. O'Brien ME, Watkins D, Ryan C, et al. A randomised trial in malignant mesothelioma (M) of early (E) versus delayed (D) chemotherapy in symptomatically stable patients: the MED trial. *Annals of Oncology* 2006;17(2):270-5. doi: 10.1093/annonc/mdj073

81. Kishi K, Homma S, Sakamoto S, et al. [High efficacy of pleurodesis using OK-432 for controlling intractable pneumothorax associated with pulmonary lymphangioleiomyomatosis]. *Nihon Kokyuki Gakkai zasshi= the journal of the Japanese Respiratory Society* 2003;41(10):704-07.

82. Kitahara S, Ikeda M, Inouye T, et al. Inhibition of head and neck metastatic and/or recurrent cancer by local administration of multi-cytokine inducer OK-432. *The Journal of Laryngology & Otology* 1996;110(05):449-53.

83. Light R, Vargas F. Pleural sclerosis for the treatment of pneumothorax and pleural effusion. *Lung* 1997;175(4):213-23.

84. Ogita S, Tsuto T, Nakamura K, et al. OK-432 therapy for lymphangioma in children: why and how does it work? *Journal of Pediatric Surgery* 1996;31(4):477-80.

85. Clayson H. Suffering in mesothelioma: concepts and contexts. *Progress in Palliative Care* 2003;11(5):251-55.

86. Lebovits A, Chahinian A, Gorzynski J, et al. Psychological aspects of asbestos-related mesothelioma and knowledge of high risk for cancer. *Cancer Detection and Prevention* 1980;4(1-4):181-84.

87. Barak Y, Achiron A, Rotstein Z, et al. Stress associated with asbestosis: the trauma of waiting for death. *Psycho-Oncology* 1997;7(2):126-28.

88. Clayson H. The experience of mesothelioma in Northern England. University of Sheffield, 2007.

89. Lebovits AH, Chahinian AP, Holland JC. Exposure to asbestos: psychological responses of mesothelioma patients. *American Journal of Industrial Medicine* 1983;4(3):459-66.

90. Moore S, Darlison L, Tod A. Living with mesothelioma. A literature review. *European Journal of Cancer Care* 2010;19(4):458-68.

91. Hudson P, Aranda S, McMurray N. Randomized controlled trials in palliative care: overcoming the obstacles. *International Journal of Palliative Nursing* 2001;7(9):427-34.

92. LeBlanc TW, Lodato JE, Currow DC, et al. Overcoming recruitment challenges in palliative care clinical trials. *Journal of Oncology Practice* 2013;9(6):277-82.

93. White C, Hardy J. What do palliative care patients and their relatives think about research in palliative care?-a systematic review. *Supportive care in cancer : official journal of the Multinational Association of Supportive Care in Cancer* 2010;18(8):905-11. doi: 10.1007/s00520-009-0724-1

94. Donovan J, Mills N, Smith M, et al. Quality improvement report: Improving design and conduct of randomised trials by embedding them in qualitative research: ProtecT (prostate testing for cancer and treatment) study. *British Medical Journal* 2002:766-69.

95. Audrey S. Qualitative research in evidence-based medicine: Improving decision-making and participation in randomized controlled trials of cancer treatments. *Palliative Medicine* 2011;25(8):758-65.

96. Clarke V, Braun V. Thematic analysis. Encyclopedia of Critical Psychology: Springer 2014:1947-52.

97. Davies HE, Mishra EK, Kahan BC, et al. Effect of an indwelling pleural catheter vs chest tube and talc pleurodesis for relieving dyspnea in patients with malignant pleural effusion: the TIME2 randomized controlled trial. *JAMA* 2012;307(22):2383-9.

98. Guest G, Bunce A, Johnson L. How many interviews are enough? An experiment with data saturation and variability. *Field Methods* 2006;18(1):59-82.

99. Francart J, Legrand C, Sylvester R, et al. Progression-Free Survival Rate As Primary End Point for Phase II Cancer Clinical Trials: Application to Mesothelioma—The EORTC Lung Cancer Group. *J Clin Oncol* 2006;24(19):3007-12. doi: 10.1200/jco.2005.05.1359
