# Supplementary material for: A trial of intra-pleural bacterial immunotherapy in malignant pleural mesothelioma (TILT) — a randomised feasibility study using the trial within a cohort (TwiC) methodology
Source: Pilot Feasibility Stud. 2022 Sep 3;8:196. doi: 10.1186/s40814-022-01156-3 (PMC9440504; doi:10.1186/s40814-022-01156-3)
Supplement: Supplementary file 2 — Additional file 2: Appendix B. Trial Specific Procedure for IMP administration. [file 40814_2022_1156_MOESM2_ESM.docx]

**Appendix B - Trial Specific Procedure for IMP administration**

This TSP is intended for use only for TILT trial participants who have been randomly allocated to receive OK432 or BCG. They are both unlicensed drugs in the UK and should not be administered to patients outside of the TILT trial.

OK432/BCG should be administered between days 0 and 14.

PLEASE NOTE, WOMEN WHO ARE PREGNANT, BREAST-FEEDING, OR PLANNING TO BECOME SO SHOULD NOT HANDLE BCG.

**Preparation**

1. A full set of bloods should be taken, and results checked prior to OK432/BCG administration. Blood test results that are acceptable for OK432/BCG administration are provided below.
2. Undertake a full drainage of participant’s IPC (see TSP 01).
3. Perform a chest x-ray
4. Perform a thoracic USS
5. Take a set of observations
6. Complete ‘Intervention visit’ eCRF
7. If participant remains eligible to receive OK432/BCG, please complete the TILT consent form, including the time and date.
8. For NBT, please now sign the pre-prepared prescription and send it to pharmacy.
9. Check patient identification & allergies
10. Give the participant 1g paracetamol orally
11. Consider giving the participant prophylactic analgesia e.g. codeine/oramorph
12. Administer OK432/BCG

**Administration**

***Equipment***

Dressing pack Sterile gloves

Disposable apron Skin cleaning solution e.g. Chlorhexadine in 70% alcohol

1 x 20ml syringe 3 x 50ml 0.9% saline

3 x 50ml syringe White/red drawing-up needle

2 x 3- way taps IPC drainage line

2 x Gauze IPC drainage bottle

Prescription chart IPC dressings

Trial IMP 2 x Adhesive dressings

Bungs/syringe caps 3mg/kg of 1% lignocaine (to a max dose of 200mg)

***Additional equipment for BCG administration***

1 ml syringe

Personal protective equipment – face mask, protective sleeves, apron, gloves

Closed system transfer device

Spillage kit

Cytotoxic sharps bin & cytotoxic bin bag

***Set up***

1. Prepare a sterile field, including a pot with skin cleaning solution, and all the equipment necessary for the procedure.
2. Remove the dressing from the IPC.
3. Wash hands and put on apron & sterile gloves.
4. Draw up 50ml of normal saline and place on sterile field.
5. Draw up 3mg/kg 1% lignocaine (max dose 200mg) in a 20ml syringe and place on sterile field.

**For OK432**

- 1. Add 2ml normal saline diluent to each vial **(nb one dose consists of 10 vials of 1KE, or 5 vials of 1KE if reduced dose of 5KE is being used)**. Allow to stand for a few minutes then gently swirl until a homogenous suspension is obtained. Forceful agitation should be avoided.
  2. Draw up 30mls of normal saline into the second 50ml syringe
  3. Aspirate the reconstituted OK432 into the syringe of saline from the vials. Rinse each vial by transferring 1ml back into the vial and then aspirating back into the syringe. Mix the suspension gently.
  4. Place syringe on sterile field

**For BCG**

1. BCG should be reconstituted at the patient bedside to limit the risk of accidental contamination of other clinical areas. It should not be prepared in areas where other parenteral drugs are prepared – e.g. treatment rooms.
2. Put on mask and sleeves
3. Draw up 50ml normal saline in the second syringe **(nb final dose consists of 1ml of reconstituted BCG, or 0.5ml if reduced dose is being used)**.
4. Using the closed system transfer device, add 1 ml of normal saline to the vial of BCG. Gently aspirate and inject 1ml saline to and from the vial until a homogenous suspension is obtained. Forceful agitation should be avoided.
5. Once the BCG is fully reconstituted, detach from closed transfer system & attach to 3-way tap with 1ml syringe attached to other port.
6. If full dose is being used, draw up 1 ml of BCG solution into 1 ml syringe. **1ml is the dose to be delivered.**
7. If half dose is being used, draw up 0.5 ml of BCG solution into 1 ml syringe. **0.5ml is the dose to be delivered.**
8. Place 1ml syringe containing BCG dose on sterile field & discard syringe containing remaining BCG into cytotoxic sharps bin.
9. Clean all external elements of IPC with gauze and cleaning solution (maintain sterility).
10. Remove the cap from the end of the IPC and leave to soak in the skin cleaning solution (unless planning to use a new cap, in which case the old cap can be disposed of).

***Administration***

1. Clean the end of the IPC with gauze and cleaning solution.
2. Attach a 3 way tap to the luer-lock end of the IPC drainage line, and ensure the drainage line is clamped.
3. Securely attach the saline of syringe to one of the unoccupied ports of the three-way tap and flush some saline through the drainage line to ensure there is no air left in it.
4. Insert the drainage adaptor into the IPC until a click is heard.
5. Release the clamp and flush 10mls of saline into drain to remove any blockage.
6. Clamp the line, attach the syringe of local anaesthetic to the unoccupied port of the 3 way tap.
7. Turn the 3 way tap so it is ‘on’ to the local anaesthetic and IPC, but ‘off’ to the saline.
8. Unclamp the line and flush local anaesthetic into the drain, followed by 10 mls of saline.
9. Clamp line, remove the adaptor from the drain and place it on the sterile field.
10. Leave anaesthetic in chest for 5-10 minutes.
11. Attach the syringe of OK432/BCG to the 3-way tap.
12. After 5 minutes, re-attach the drainage line to the IPC and slowly flush the OK423/BCG into the IPC.
13. Once all the OK432/BCG has been administered, over 5 minutes flush the remaining 40ml of saline into the IPC, to ensure none of the drug remains in the IPC tubing.
14. Remove the adaptor and clean the access port. Replace the cap securely.
15. Replace the dressing.

***Observation***

1. Allow the OK432/BCG to remain in the thoracic cavity for 1 hour.
2. Undertake clinical observations every 15 minutes while the drug is in situ.
3. Towards the end of the hour, draw up the final 50ml syringe of saline.
4. After 1 hour, drain the IPC (see TSP 01) and flush with 50ml saline.
5. Replace IPC cap and dressing.
6. Observe participant for another hour, undertaking observations every 30 minutes.
7. If the participant is well after 1 hour, they may go home.
8. Please ensure they have an appointment for their Week 3 follow up visit.
9. Please ensure they have a VAS booklet, and know to complete it daily until their next visit.
10. Advise patient to take regular paracetamol (1g QDS) and ibuprofen (400mg TDS), prophylactically for the next 3 days (unless contra-indicated).
11. Ensure patient has trial team and out of hours contact numbers in case of adverse events.

**Disposal – OK432**

1. Empty OK432 vials and needles should be disposed of into a standard sharps bin.
2. Equipment used for OK432 administration, including syringes, drainage lines, gauze, dressings, gloves & gown should be disposed of into a clinical waste bin.
3. The drainage bottle containing fluid drained at the end of the procedure should be disposed of in a clinical waste bin.
4. Unused vials of OK432 (i.e. OK432 that has been dispensed by pharmacy but not opened or administered) should be returned to pharmacy.

**Disposal - BCG**

1. Empty BCG vials, needles used for BCG administration and syringe of unused BCG solution should be disposed of into a cytotoxic sharps bin.
2. Equipment used for BCG administration, including syringes, drainage lines, paper/gauze contaminated with BCG, gloves, gowns, masks & eyewear should be double-bagged in cytotoxic bags and sent for incineration.
3. The drainage bottle containing fluid drained at the end of the procedure should be disposed of in a cytotoxic waste bin.
4. Unused vials of BCG (i.e. BCG that has been dispensed by pharmacy but not opened or administered) should be returned to pharmacy.

**Accidental spillages & contamination – OK432**

OK432 is a not a biohazard, therefore any spillages or contamination should be managed in line with usual trust policy. Risk of infection after accidental inoculation is minimal, however the trust policy on accidental inoculation should be followed and Occupational Health should be informed.

**Accidental spillages & contamination – BCG**

Spillage kits must be available in any area where BCG is being administered.

BCG exposure should not produce significant adverse health outcomes in healthy individuals. However, in case of accidental self-inoculation, PPD/Tuberculin skin testing is advised at the time of the accident and six weeks later to detect skin test conversion. Occupational Health must be informed of any spillages/ inoculation in which a staff member has come into contact with BCG.

***Work surfaces***: Apply disposable towels soaked with tuberculocidal disinfectant (Hyperchloride solution/bleach) to affected area for at least 10 minutes. All waste materials should be disposed of as biohazard material.

***Clothing contamination***: If any clothing has been contaminated, it should be removed as soon as possible, placed into two yellow bags and sealed with cytotoxic tape and sent for incineration.

***Skin contact:*** Wash thoroughly with soap and water. Clean any open sores or lacerations with alcohol.

***Eyes:*** Flush with copious amounts of water for at least 15 minutes. Rinse eyes using eye wash provided in spillage kit. Inform medical staff and occupational health.

***Inhalation:*** Leave area of contamination, seek fresh air if possible. Seek medical evaluation. Inform occupational health.

**Troubleshooting/potential problems**

**Abnormal observations at baseline**

- The participant’s observations must be within the following parameters at baseline:
  - HR 40 - 110 bpm
  - RR 10 - 24
  - Systolic BP 100 - 160 mmHg
  - Saturations > 92% on air (or on stable long term oxygen therapy)
  - Temperature < 38 ⁰C
- If any observations are outside these ranges, participant is not eligible for OK432 administration. Please document this on “Intervention visit” eCRF & complete a trial deviation form.
- Participant should continue follow up at days week 3, 6 and 12 (unless they withdraw from the study).

**Abnormal blood test results**

- The following blood tests must be within the stated ranges for OK432/BCG to be delivered:

WBC ≤ 12.5 x 10^9^/L and ≥ 3.5 x 10^9^/L

eGFR ≥ 40mls/min

CRP ≤ 100

ALT ≤ 90 U/L

- OK432/BCG can be delivered in the presence of abnormal blood tests (with the exception of those listed above) at the discretion of the treating clinician, if they are felt to be non-clinically significant or related to the underlying disease process.
- Clinicians are welcome to discuss any queries about whether OK432/BCG can be delivered in the presence of deranged blood tests with Dr Anna Bibby.

**Intercurrent infection**

- If the participant has signs or symptoms suggestive of intercurrent infection (pleural or elsewhere), they are not eligible for OK432/BCG administration. Please document this on “Intervention visit” eCRF.
- For some participants it may be appropriate to treat the infection, and arrange for OK432/BCG to be administered after a sufficient recovery period (e.g. 2 weeks after completion of antibiotics). This will mean OK432/BCG cannot be given within 7 days of randomisation as specified in the protocol. If this occurs, please administer OK432/BCG as soon as deemed safe by the PI, and complete a trial deviation form.
- Participant should continue follow up at Week 3, 6 and 12.

**Contra-indicated medication**

Medications that are contra-indicated in conjunction with OK432 and BCG include:

- Chemotherapy or immunotherapy
- Systemically active steroids for ≥3 days
- Granulocyte colony stimulating factor (GCSF) or Granulocyte macrophage colony-stimulating factor
- Other known immunosuppressive or immunomodulatory medication

If a participant is currently taking any of these medications, or has taken them in the preceding 4 weeks, they are not eligible to receive OK432 or BCG. Please document this on “Intervention visit” eCRF. Participant should continue follow up at Week 3, 6 and 12.

**Blocked/non-draining IPC**

- If the IPC is blocked, or not draining, please attempt to flush it with normal saline (see TSP 02).
- If it is possible to flush the IPC, you may proceed with OK432/BCG administration.
- If the IPC cannot be flushed, please attempt to unblock it with urokinase (see TSP 02).
- If unblockage is successful, OK432/BCG can be administered.
- It is recognised that the process of unblocking an IPC will introduce delay, and may mean OK432 cannot be given within 7 days of randomisation as specified in the protocol. If this occurs, please administer OK432/BCG as soon as possible after IPC unblockage, and complete a trial deviation form.
- If IPC cannot be unblocked, OK432/BCG cannot be administered. Please document this on “Intervention visit” eCRF. Participant should continue follow up at Week 3, 6 and 12.

**Resolution of pleural effusion**

- If a participant’s pleural effusion has resolved or if there is no fluid present on the day of the intervention visit, please attempt to flush the IPC with normal saline (see TSP 02).
- If it is possible to flush the IPC, you may proceed with OK432/BCG administration.
- If IPC cannot be flushed, OK432/BCG cannot be administered. Please document this on “Intervention visit” eCRF. Participant should continue follow up at Week 3, 6 and 12.

**Trapped lung on CXR**

- If CXR shows trapped lung, with less than 50% pleural apposition, the participant is not eligible to receive OK432/BCG. Please document this on “Intervention visit” eCRF. Participant should continue follow up at Week 3, 6 and 12.
- If CXR shows trapped lung, but more than 50% of the pleural surface is in contact, the participant is eligible to receive OK432/BCG. Please document this on the “Intervention” eCRF and proceed with OK432/BCG administration.

**Moderate or heavily loculated effusion on US**

If the participant has a moderate or heavily loculated effusion on US, they are not eligible to receive OK432/BCG. Please document this on the “Intervention” eCRF. Participant must continue follow up at days 35 and 84.

**Adverse reactions**

- Anaphylaxis and severe adverse reactions should be managed in accordance with local and national policy, see guidelines from the UK Resuscitation Council (available at <https://www.resus.org.uk/anaphylaxis/emergency-treatment-of-anaphylactic-reactions/>).
- If a severe adverse reaction occurs while OK432/BCG is in situ in the chest cavity, please drain the IPC immediately.
- The following adverse reactions are expected following OK432/BCG administration, and should be managed with appropriate analgesia, antipyretics, antihistamines and fluids as required.
  - Fever (≤39°C)
  - Mild tachycardia (≤20 beats per minute over baseline)
  - Chest pain requiring analgesia
  - Tachypnoea (increase in respiratory rate of ≥4 breaths per minute over baseline)
  - Hypoxia (to saturation of ≥92% on air, or to a level requiring supplemental oxygen)
- If a non-severe adverse reaction occurs whilst OK432/BCG is in situ in the chest cavity, the IPC does not need to be drained, unless the participant deteriorates, or the treating clinician is concerned about potential deterioration.
- Please document all AR on “Intervention visit” eCRF, in the participant’s medical notes and on the adverse event log.
- If the reaction meets the criteria for SAE, SAR or SUSAR, please complete the relevant form (see TSP 06) and return to the Sponsor within 24 hours, with a copy to the TILT administrator at the Academic Respiratory Unit, North Bristol.
