# Supplementary material for: A trial of intra-pleural bacterial immunotherapy in malignant pleural mesothelioma (TILT) — a randomised feasibility study using the trial within a cohort (TwiC) methodology
Source: Pilot Feasibility Stud. 2022 Sep 3;8:196. doi: 10.1186/s40814-022-01156-3 (PMC9440504; doi:10.1186/s40814-022-01156-3)
Supplement: Supplementary file 3 — Additional file 3: Appendix C. Average IPC drainage volumes (mean & 95% confidence intervals) at each trial visit for participants randomised to receive OK432 or BCG (IMP group) and controls. [file 40814_2022_1156_MOESM3_ESM.docx]

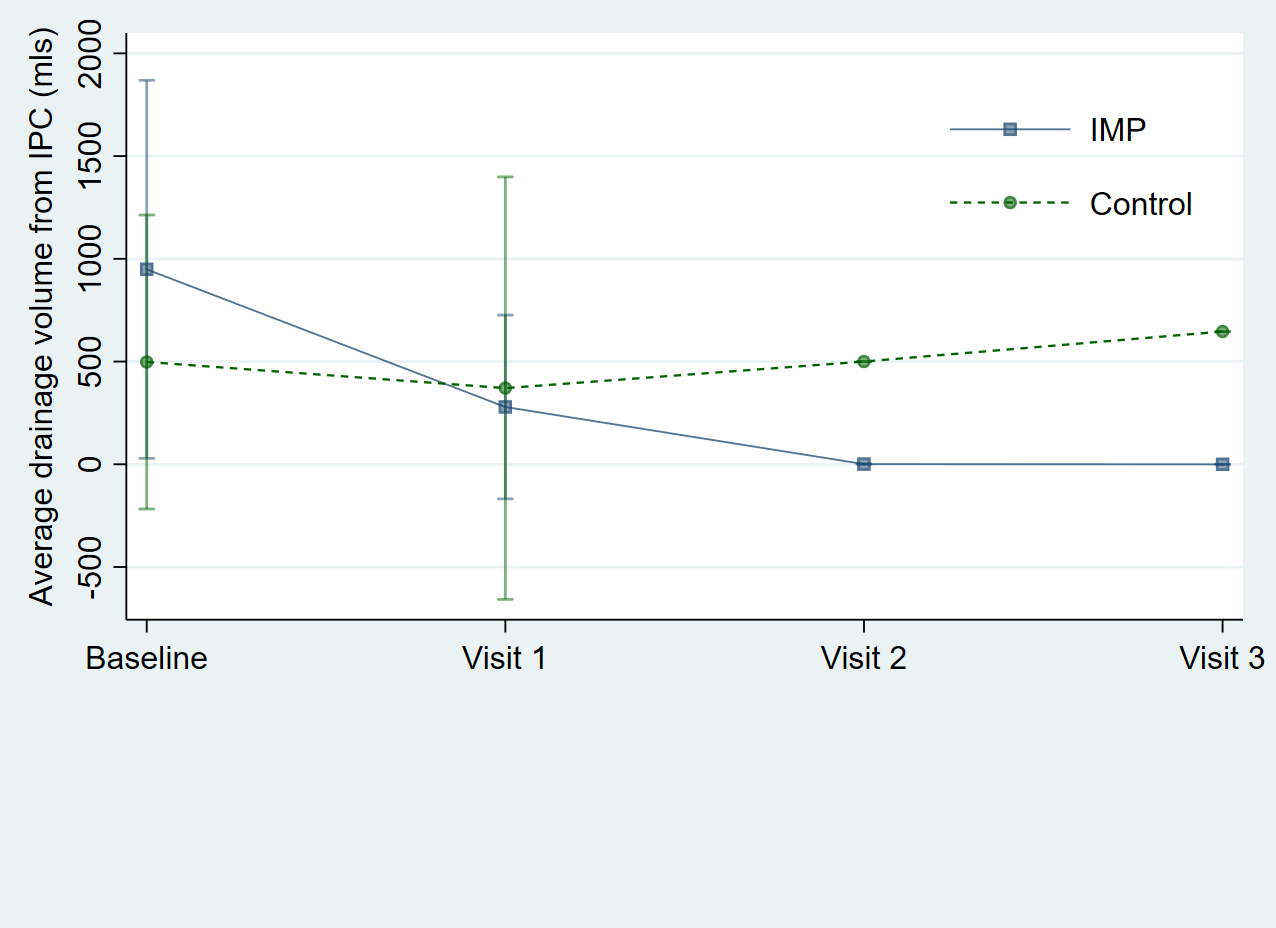


Appendix C - Average IPC drainage volumes (mean & 95% confidence intervals) at each trial visit for participants randomised to receive OK432 or BCG (IMP group) and controls.
