# Supplementary material for: A trial of intra-pleural bacterial immunotherapy in malignant pleural mesothelioma (TILT) — a randomised feasibility study using the trial within a cohort (TwiC) methodology
Source: Pilot Feasibility Stud. 2022 Sep 3;8:196. doi: 10.1186/s40814-022-01156-3 (PMC9440504; doi:10.1186/s40814-022-01156-3)
Supplement: Supplementary file 4 — Additional file 4: Appendix D. Results for patient-reported outcome measures. Figure C1. Patient reported symptom scores (mean and 95% confidence intervals) for breathlessness in people randomised to receive an IMP compared with controls at each study visit. Figure C2. Patient reported symptom scores (mean and 95% confidence intervals) for chest pain for people randomised to receive an IMP compared with controls at each study visit. Figure C3. Patient reported symptom scores (mean and 95% confidence intervals) for sweats in people randomised to receive an IMP compared with controls at each study visit. Figure C4. 1 Patient reported symptom scores (mean and 95% confidence intervals) for quality of life in people randomised to receive OK432 or BCG compared with controls at each study visit. [file 40814_2022_1156_MOESM4_ESM.docx]

**Appendix C – Results for patient-reported outcome measures**

|  | | **Trial visit** | | | |
| --- | --- | --- | --- | --- | --- |
|  |  | **1** | **2** | **3** | **4** |
| **Breathlessness** | IMP | 36  (-28.4 to 100.4) | 29.8  (-31.5 to 91.0) | 13.6  (-9.5 to 36.8) | 20.3  (-4.5 to 45.1) |
|  | Control | 17.3  (-30.5 to 65.1) | 3.0  (-14.6 to 20.6) | 0  (-17.6 to 17.6) | 19.3  (-27.4 to 66.1) |
| **Chest pain** | IMP | 16.6  (-46.6 to 79.8) | 10.5  (-29.4 to 50.4) | 2  (-3.3 to 7.3) | 11.8  (-23.4 to 47.0) |
|  | Control | 5.7  (-3.4 to 14.8) | 0  (n/a) | 1  (n/a) | 3.7  (-7.1 to 14.5) |
| **Sweating** | IMP | 0.6  (-1.2 to 2.5) | 14.5  (-30.2 to 59.2) | 10  (-19.2 to 39.2) | 1.3  (-0.9 to 3.6) |
|  | Control | 5.8  (-4.9 to 16.6) | 0  (n/a) | 0  (n/a) | 3.0  (-7.2 to 13.2) |
| **Quality of life** | IMP | 80  (64.0 to 96.0) | 77.5  (37.9 to 117.1) | 76.3  (35.9 to 116.5) | 83.3  (58.7 to 108.0) |
|  | Control | 80  (37.3 to 122.7) | 90  (76.1 to 103.9) | 80  (52.3 to 107.7) | 66.7  (25.8 to 107.5) |


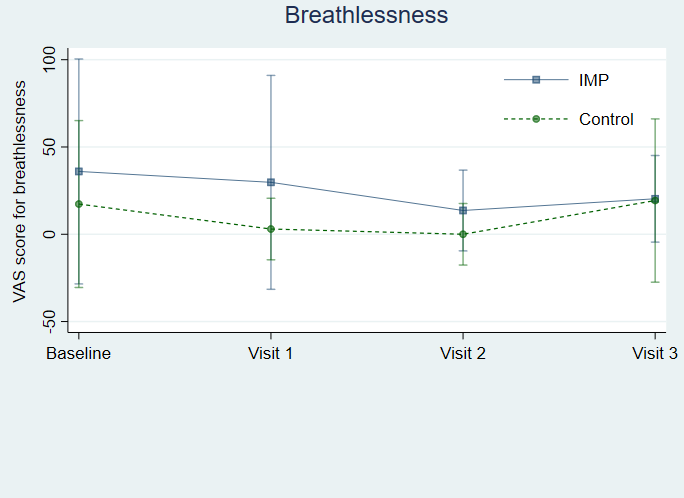


Figure C1 - Patient reported symptom scores (mean and 95% confidence intervals) for breathlessness in people randomised to receive an IMP compared with controls at each study visit


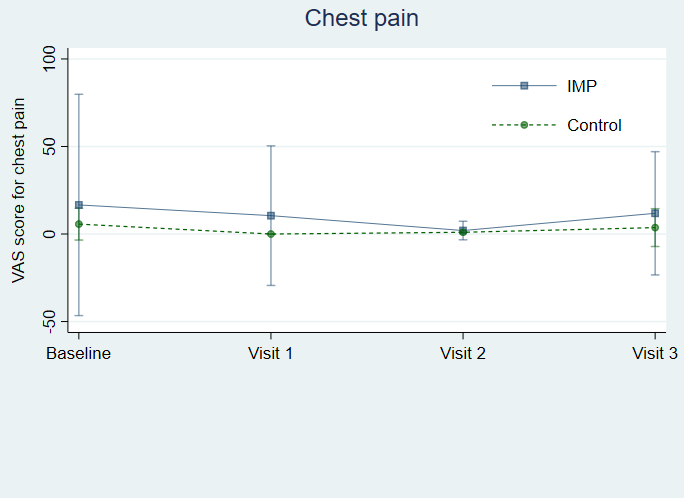


Figure C2 - Patient reported symptom scores (mean and 95% confidence intervals) for chest pain for people randomised to receive an IMP compared with controls at each study visit


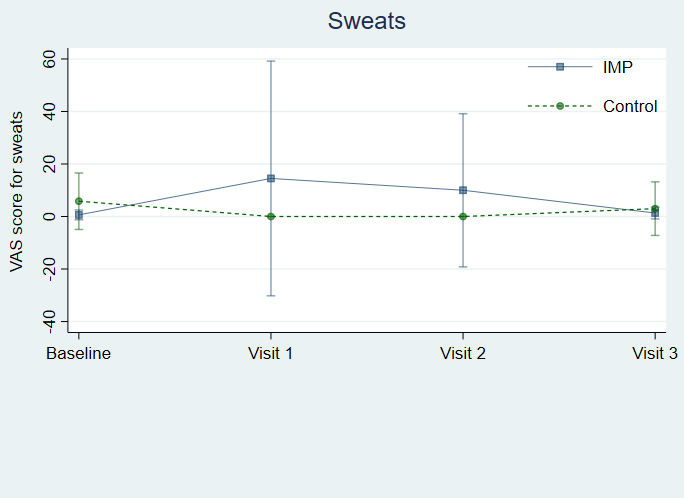


Figure C3 - Patient reported symptom scores (mean and 95% confidence intervals) for sweats in people randomised to receive an IMP compared with controls at each study visit


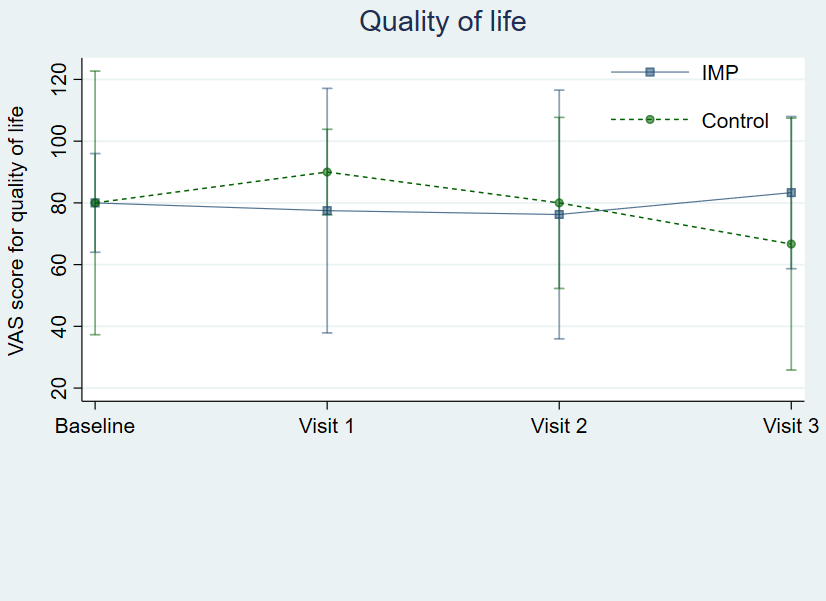


Figure C4 - Patient reported symptom scores (mean and 95% confidence intervals) for quality of life in people randomised to receive OK432 or BCG compared with controls at each study visit
