# Supplementary material for: A trial of intra-pleural bacterial immunotherapy in malignant pleural mesothelioma (TILT) — a randomised feasibility study using the trial within a cohort (TwiC) methodology
Source: Pilot Feasibility Stud. 2022 Sep 3;8:196. doi: 10.1186/s40814-022-01156-3 (PMC9440504; doi:10.1186/s40814-022-01156-3)
Supplement: Supplementary file 5 — Additional file 5. PPI statement. [file 40814_2022_1156_MOESM5_ESM.docx]

When and how were patients/public first involved in the research?

● How were the research question(s) developed and informed by their priorities,

experience, and preferences?

● How were patients/public involved in

○ (a) the design and conduct of the study?

○ (b) choice of outcome measures?

○ (c) recruitment to the study?

○

● How were (or will) patients/ public be involved in choosing the methods and agreeing

plans for dissemination of the study results to participants and linked communities?

You may find this link helpful.
